# ^19^F NMR‐Based Fragment Screening for 14 Different Biologically Active RNAs and 10 DNA and Protein Counter‐Screens

**DOI:** 10.1002/cbic.202000476

**Published:** 2020-09-25

**Authors:** Oliver Binas, Vanessa de Jesus, Tom Landgraf, Albrecht Eduard Völklein, Jason Martins, Daniel Hymon, Jasleen Kaur Bains, Hannes Berg, Thomas Biedenbänder, Boris Fürtig, Santosh Lakshmi Gande, Anna Niesteruk, Andreas Oxenfarth, Nusrat Shahin Qureshi, Tatjana Schamber, Robbin Schnieders, Alix Tröster, Anna Wacker, Julia Wirmer‐Bartoschek, Maria Alexandra Wirtz Martin, Elke Stirnal, Kamal Azzaoui, Christian Richter, Sridhar Sreeramulu, Marcel Jules José Blommers, Harald Schwalbe

**Affiliations:** ^1^ Institute for Organic Chemistry and Chemical Biology Center for Biomolecular Magnetic Resonance (BMRZ) Johann Wolfgang Goethe-University Frankfurt Max-von-Laue Strasse 7 60438 Frankfurt am Main Germany; ^2^ Saverna Therapeutics Gewerbestrasse 24 4123 Allschwil Switzerland

**Keywords:** DNA, ^19^F, FBS, fluorine, fragment-based screening, proteins, RNA

## Abstract

We report here the nuclear magnetic resonance ^19^F screening of 14 RNA targets with different secondary and tertiary structure to systematically assess the druggability of RNAs. Our RNA targets include representative bacterial riboswitches that naturally bind with nanomolar affinity and high specificity to cellular metabolites of low molecular weight. Based on counter‐screens against five DNAs and five proteins, we can show that RNA can be specifically targeted. To demonstrate the quality of the initial fragment library that has been designed for easy follow‐up chemistry, we further show how to increase binding affinity from an initial fragment hit by chemistry that links the identified fragment to the intercalator acridine. Thus, we achieve low‐micromolar binding affinity without losing binding specificity between two different terminator structures.

## Graphical abstract

**Checking out RNA druggability by NMR**: Fourteen RNAs have been screened and ten DNA and proteins were counter‐screened to show that fragment libraries can differentiate between different RNA targets with up to 15‐fold selectivity.

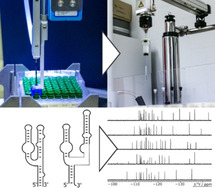

## Introduction

Proteins constitute the vast majority of validated drug targets. The ribosome, a large RNA‐protein complex, is the most prominent RNA drug target. Most antibiotics inhibit protein synthesis by targeting the interface of RNA and proteins in the ribosome.^[1]^ Beyond being target for antibiotics, RNA has for long been considered undruggable. Recently, however, this view has changed and RNA emerged as a potential target for drug discovery as well.[^2^, ^3^, ^4^, ^5^, ^6^] Clinical success of compounds initially identified as RNA binders^[7]^ inspires thorough exploration of the noncoding RNA target space. Here, potential targets range from RNA involved in oncology and inflammation to RNA involved in bacterial and viral infections, to mention a few areas with unmet medical need. Concurrently, the continuous identification of new regulatory RNAs, including riboswitches or sRNAs further increases potential applications.^[8]^ To combat multidrug‐resistant (MDR) bacteria that pose a major health threat for modern human society,^[9]^ Riboswitches in particular have come into focus, which can only be tackled by new antibiotics. The development of drugs targeting riboswitches is therefore an important research focus.^[10]^

Structure‐based drug discovery is a key methodology for rational drug discovery. Here, X‐ray crystallography^[11]^ and nuclear magnetic resonance (NMR) spectroscopy^[12]^ provide the essential structural information. Insight from target structures is often supported by computational methods to aid in library design. Virtual screening by *in silico* docking of a compound library against available target model structures guides medicinal chemistry in the development from initial hits towards the generation of lead compounds.^[13]^

High‐throughput screening in drug discovery requires robust detection of binding of a large number of test compounds, a library, to a biological target. Such screening can involve up to 1–2 million compounds from which routinely a very small number of potential lead compounds are identified. Screening large libraries thus requires high preparative and infrastructural effort.

An alternative to this classic approach is fragment‐based screening. Fragments are often weak binders and their binding specificity can be lower than expected from a lead compound.^[14]^ Thus, fragment‐based drug discovery requires that the initial hits are further processed into lead compounds by chemical modification such as growing the compound to fit the desired number of binding interactions with the target or linkage of two fragments which bind to binding sites in spatial proximity. Fragment‐based drug discovery is nowadays the basis of many hit‐to‐lead research programs.^[15]^ In fact, it has been shown that FDA‐approved drugs targeting proteins can be developed starting from fragment screens.[^15^, ^16^, ^17^, ^18^, ^19^, ^20^, ^21^]

**Figure 1:**
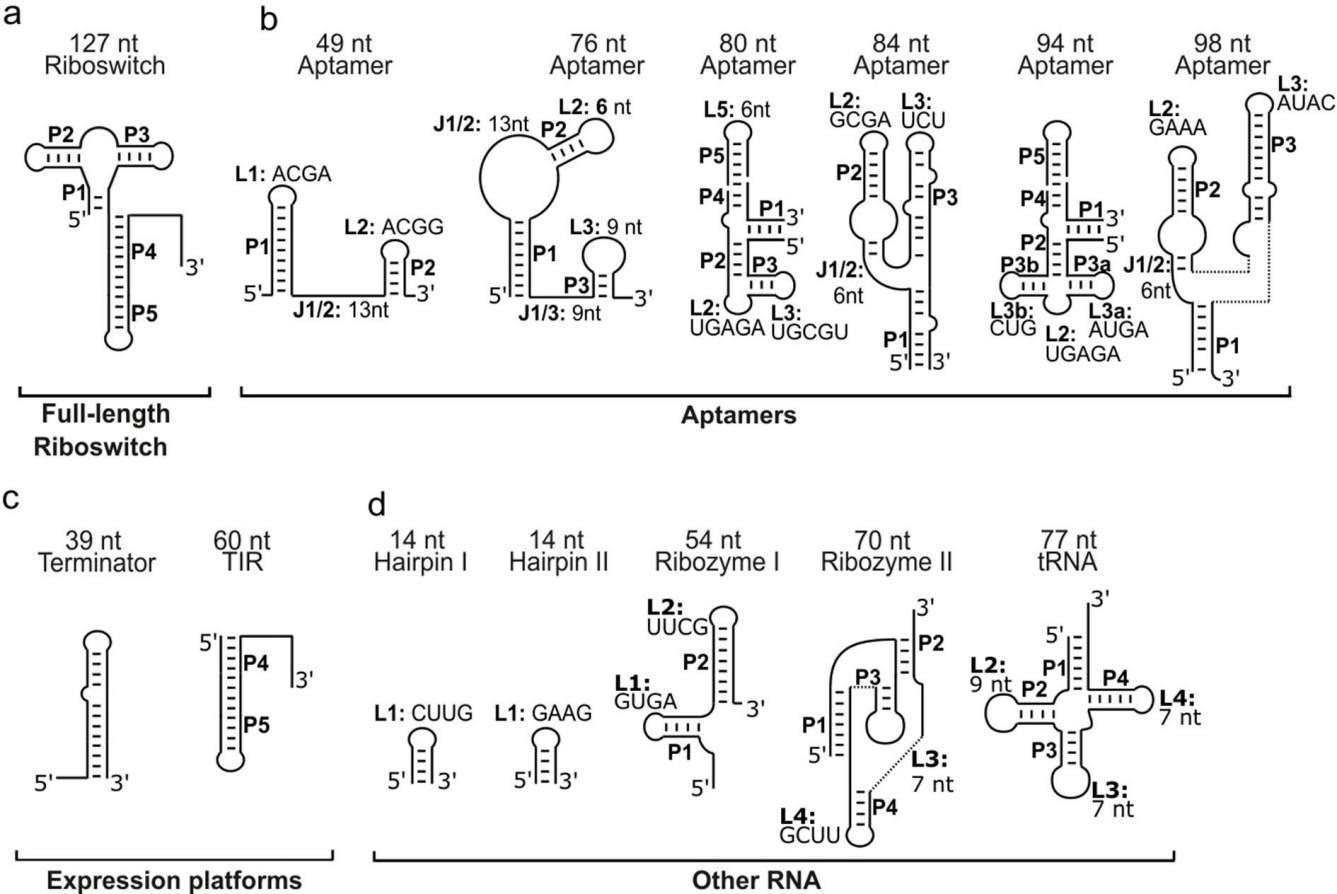
Overview of RNA targets. Schematic secondary structures of the RNA targets investigated by ^19^F fragment binding studies (FBS). Stems (P), loops (L) and junctions (J) are annotated. Tri‐, tetra‐ and pentaloop sequences are listed explicitly. Rational ligand design led to the development of compounds that specifically bind to an RNA loop region.^[37]^ In our study, we included two 14‐nt stem‐loop structures exhibiting a GAAG and a CUUG tetraloop, respectively in order to detect fragments binding to this abundant secondary structure motif. Also, we included the guanidine‐sensing riboswitch as an example of a functional RNA with hairpin structures. Loop–loop interactions^[38]^ are part of the stabilizing function with purine‐sensing riboswitches that are part of the RNA targets in this study (Figure S3).

Methods used to screen RNA include fluorescence‐based assays,[^22^, ^23^] mass spectrometry,[^24^, ^25^] small‐molecule microarrays (SMM),[^26^, ^27^] microscale thermophoresis (MST)[^28^, ^29^] and NMR spectroscopy.^[30]^ However, most of these studies focus only on a single target RNA.[^31^, ^32^] In contrast, we report herein the screening of a library of 102 fragments against 14 different RNAs of different sizes and different architectures by using ^19^F NMR as the main method for hit identification. The targeted RNAs include small stem loop structures, aptamer domains of riboswitches, full‐length riboswitches, terminators and antiterminators of riboswitches, ribozymes as well as tRNAs, traditionally serving as control RNAs in screening (Figure 1). Thus, our results allow us to delineate specificities of fragments towards different RNAs. We further counter‐screened against five DNAs and five proteins to test whether the fragment library can target for these different classes of biomacromolecules or whether the library is biased towards binding a subset of biomacromolecules. The DNA targets include regular double‐stranded DNA as well as G‐quadruplex structures of different morphology, and the proteins include RNA‐binding proteins as well as the important enzyme classes kinases and phosphatases that bind to phosphorylated moieties as part of the enzymatic function. With our multi‐target approach, we show that selective fragments for an RNA target can be found but often a good hit is broadly binding to RNAs of the same size and structural complexity. Also we show that the selective targeting of RNA over other classes of biomacromolecules is possible with this library.

By way of example, we further show that by fast follow‐up chemistry, that involves the linkage of an RNA‐binding fragment with the intercalator acridine, we obtain low micromolar RNA binders with more than tenfold specificity towards different RNAs.

## Results and Discussion

Target choice is an important step for any screening, especially in a multi‐target approach in which a broad spectrum of biologically relevant target molecules is crucial to success. Therefore, we showcase our measures of target choice in the following section. All RNA constructs screened are summarized in Table 1.

**Table 1:** List of all biomolecules used in the study listed with their biological host organism (if applicable), PDB accession codes of X‐ray structures and primary publication. *only homologue structures available. #only aptamer structures or single domains available.

	Organism	X‐ray	NMR
Riboswitches and Aptamers			
Guanidine (Gdn‐II)‐sensing riboswitch (49 nt)	*Escherichia coli*	5NDI^[73]^	
ZMP‐sensing riboswitch (76 nt)	*Thermosinus carboxydivorans*	4ZNP^[74]^	
thiM TPP‐sensing riboswitch (80 nt)	*E. coli*	2GDI^[75]^	
pilM 3′, 3′‐cGAMP‐sensing riboswitch (84 nt)	*Geobacter metallireducens*	4YAZ*^[76]^	
TenA TTP‐ sensing riboswitch (94 nt)	*Staphylococcus aureus*		
cyclic di‐GMP‐1 riboswitch (98 nt)	*Clostridium difficile*	3MXH*^[77]^ 3IRW*^[78]^ 3IWN*^[79]^	
Adenine‐sensing riboswitch (127 nt)	*Vibrio vulnificus*	1y26#^[80]^ 5E54#^[81]^ 4TZX#^[82]^	^[49]^ ^[69]^

Riboswitch Elements			
2′‐deoxyguanosine‐sensing‐riboswitch terminator (39 nt)	*Mesoplasma florum*		^[83]^
SAM‐sensing riboswitch anti‐terminator (38 nt)	*Bacillus subtilis*		
Adenine‐sensing riboswitch terminator (51 nt)	*B. subtilis*		^[84]^
Adenine‐sensing riboswitch expression platform (60 nt)	*V. vulnificus*		^[69]^ ^[85]^

Other RNAs			
RNA with GAAG tetraloop (14 nt)	*artificial*		2F87*^[86]^
RNA CUUG tetraloop (14 nt)	*artificial*		1RNG*^[87]^
Hammerhead ribozyme (54 nt)		1MME^[88]^	
Hepatitis delta virus ribozyme (70 nt)	*Hepatitis delta virus*	1DRZ^[89]^	
tRNAfMet (77 nt)	*E. coli*	^[90]^

DNA			
cMyc G‐quadruplex (22 nt)	*Homo sapiens*		1XAV^[91]^ 2L7V^[92]^
cKit G‐quadruplex (24 nt)	*H. sapiens*	3QXR*^[93]^ 2WO2	2O3M^[94]^
DNA duplex (24 nt)	*artificial*	1BNA^[95]^	
Tel26 G‐quadruplex (26 nt)	*H. sapiens*		2HY9^[96]^ 5Z80^[97]^
wtTel26 G‐quadruplex (26 nt)	*H. sapiens*		5MVB^[98]^ 2JPZ^[99]^

Proteins			
Mycobacterium tuberculosis protein tyrosine phosphatase A (MptpA, 18 kDa)	*Mycobacterium tuberculosis*	1U2P^[100]^	2LUO^[101]^
Protein tyrosine kinase A (PtkA, 30 kDa)	*M. tuberculosis*		6F2X^[102]^
Receptor tyrosine kinase EphA2 (34 kDa)	*H. sapiens*	5I9U^[103]^	
Ribosomal protein S1 (61 kDa)	*V. vulnificus*		2MFI*#^[104]^ 2KHI*#^[105]^
T7 RNA polymerase (100 kDa)	*Escherichia phage T7*	1MSW^[106]^

### RNA hairpin structures

Stem‐loop/hairpin structures represent the most common small secondary structure motifs in RNA.^[33]^ Common loop lengths range from three to seven nucleotides, but more than 50 % of all loops are tetraloops.^[34]^ Tetraloops are not only very abundant, they also exhibit a high thermodynamic stability as they are usually stabilized by hydrogen bonding and stacking interactions. When drug‐screening approaches are employed on biologically relevant stem‐loops, further characterization of the binding mode is needed to distinguish stem‐binding^[35]^ from loop‐binding ligands,^[27]^ to understand the structural basis of the ligand‐induced change in biological function.^[36]^

### RNA bulges, internal loops and pseudoknots

Helix‐junction‐helix (HJH) structure elements occur between two helices or three‐ and four‐way junctions. They can be divided into bulges and internal loops, where the first is characterized by short single‐stranded intersections on one side of an RNA stem and the latter features unpaired regions on both sides of the stem.[^39^, ^40^] Bulges and internal loops constitute conformational hinges, allowing helices to adopt different conformations with respect to each other. Formation of these structures allows for inter helix motions such as dynamic nucleobase stacking or rotation in and out of a junction.^[39]^ They can often be targeted by low‐molecular‐weight ligands as previously shown for the Tat‐TAR interaction, which was mimicked by arginamide.[^41^, ^42^] In drug screening approaches, internal loops and bulges can be valuable targets for ligand design since their potential for ligand recognition is relatively high. Examples of virtual screenings directed on HIV‐1 transactivation response element (TAR) show that sampling of the entirety of the allowed topological space leads to an ensemble of discrete conformations that can bind different ligands.[^36^, ^43^, ^44^]

In our screening pool, several examples of small and large bulge regions, internal loops and pseudoknots are present in RNAs (Figure 1).

### Riboswitches

Riboswitches are structured RNA elements which regulate gene expression by allosteric structural re‐arrangements of an expression platform element in response to sensing environmental changes by an aptamer element. Most riboswitches respond to changes in concentration of small molecules, mostly metabolites, which they bind with remarkable specificity.[^45^, ^46^] Examples showing the observation of binding via homonuclear and heteronuclear 2D NMR spectroscopy are displayed in Figures S2 and S3 in the Supporting Information. Intricate tertiary structures are formed by most aptamers to achieve high‐affinity binding required for optimal sensitivity, alongside with sufficient discrimination against noncognate ligands. These binding pockets feature a closely defined chemical space, which is usually thoroughly described by structural data (Figure S1). The complex and specific chemical environments aid development of specific ligands and therefore especially fragment‐based drug discovery approaches, which rely on chemical adaption in particular. Thus, it is not surprising that riboswitch aptamer domains have been identified as excellent drug targets very early on.[^47^, ^48^] Of the 14 RNAs screened, eight are derived from riboswitches including natural ligand binding aptamer domains (*vide infra*).

In this study, we screened the aptamer domains of riboswitches from the second‐messenger‐sensing class, the guanidinium‐sensing class, the purine‐sensing class and the thiamin‐pyrophosphate‐(TPP)‐sensing riboswitch. The TPP‐sensing riboswitch represents the most abundant riboswitch found in different prokaryotes and even eukaryotes (Table 1). For purine‐sensing riboswitches, operating either on the transcriptional or the translational level, we have previously reported on the mechanism of full‐length riboswitch function.[^49^, ^50^]

### Counter screens

To maximize the coverage of conformational space and to rule out unspecific binding, we added five other RNAs ranging from 14 to 77 nt in length to the pool of target RNAs. We screened tRNA^fMet^ produced in house. tRNAs, being ubiquitously present in all kingdoms of life, are used as counter screen RNA in many applications and especially in high‐throughput screens of RNA molecules, such as an RNA G‐quadruplex^[51]^ and the TAR RNA.^[52]^ To rule out binders not specific to RNA, we additionally screened five DNAs (Figures S24–S31), including four G‐quadruplexes and five proteins with molecular weights ranging from 18 to 100 kDa (Figure S32–S39).

### ^19^F‐CPMG based screening by NMR spectroscopy

NMR spectroscopy is well suited for the identification of initial fragment hits as it is fast and reliable and offers the possibility to detect weak binding in solution. There are a large number of NMR experiments to detect binding, including detection of NOEs, chemical shift perturbation, saturation transfer difference,^[53]^ WaterLOGSY^[54]^ or T_2_‐relaxation spectroscopy.^[55]^ By these methods, interactions characterized by dissociation constants in the range of 10 mM down to low‐nanomolar can be detected, depending on experimental setup. Further improvements are currently developed with more sophisticated methods of dynamic nuclear polarization (DNP) or hyperpolarization,^[56]^ decreasing the lower detection limit. Additionally, NMR offers the possibility to observe the interaction of the target with fragments in a mix of several fragments at the same time, greatly reducing operational effort. Often, NMR screenings use ^1^H detection. The high number of hydrogen atoms in fragment compounds, however, leads to severe overlap of NMR signals, reducing the number of fragments within one mixture that can be screened in a single experiment.

**Figure 2:**
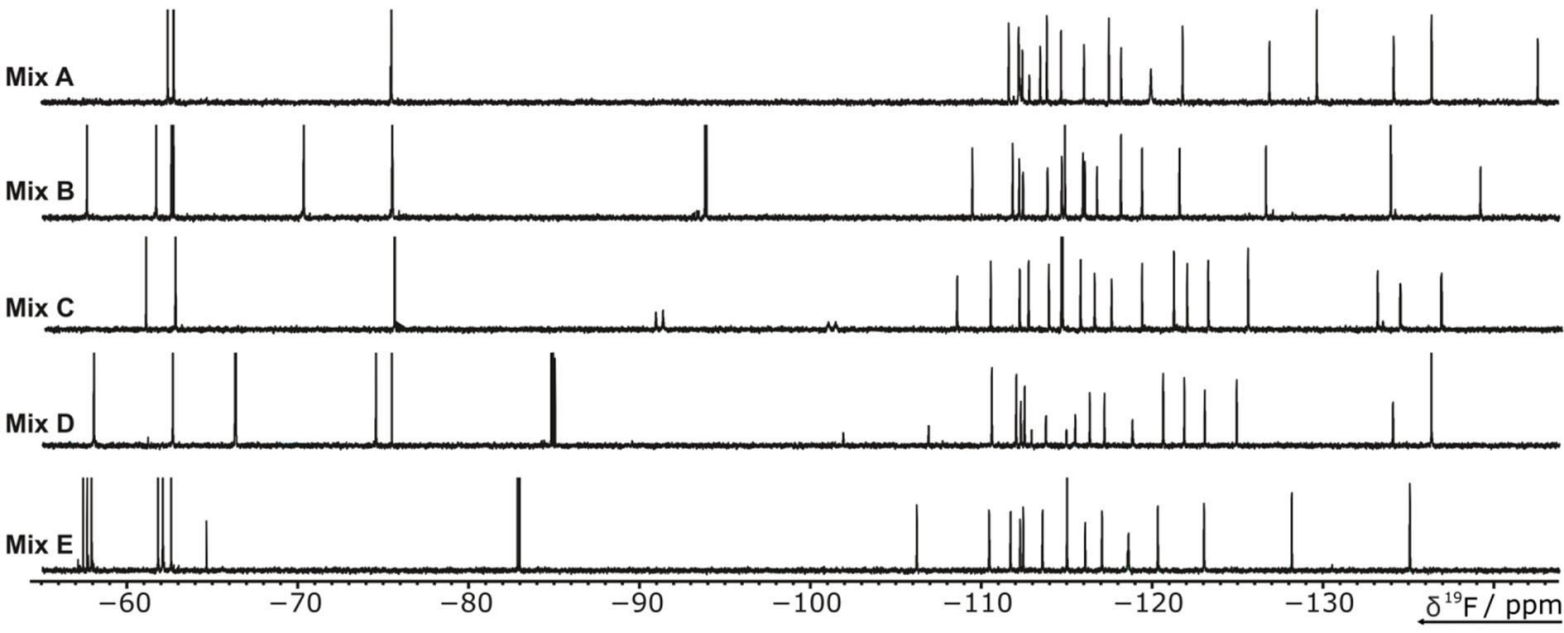
^19^F 1D NMR‐spectra of the ^19^F library fragment mixtures. The ^19^F library contains 101 compounds (Table S1). Five mixtures of either 20 or 21 ligands were generated to avoid signal overlap. The spectra of the mixtures (A–E) in the screening buffer are displayed.

^19^F detection is an attractive alternative to ^1^H‐detection.^[57]^ NMR signals in ^19^F spectra show a much higher chemical shift dispersion covering a range of around 50 kHz (83 ppm) compared to around 6 kHz (10 ppm) for protons. Furthermore, if ^1^H decoupling can be performed, which depends on the spectrometer configurations and NMR probe head used, each ^19^F resonates at a single resonance frequency, allowing the observation of several fragments in a single mixture. Figure 2 shows ^19^F‐1D spectra demonstrating the design of the fragment mixtures containing 20 or 21 different ligands per mixture. An overview of all 101 fragments screened is available in Table S1. In this study, we measured ^19^F transverse relaxation experiments which apply CPMG pulse trains[^55^, ^58^] for varying relaxation delays.

CPMG *T*_2_ measurements exploit the different relaxation properties of unbound fragments in comparison to (transiently) bound fragments to biological targets. Low‐molecular‐weight fragments with short rotational correlation times (*τ*_c_) in solution will show changes in CPMG *T*_2_ values upon (transient) binding to a high‐molecular weight macromolecule (4–100 kDa), which exhibits much slower *τ*_c_ and consequently faster *T*_2_ relaxation. This relaxation effect is observed even if the population of bound fragment is 1 % or lower. We chose this method since it is very sensitive to even low‐affinity interactions and is therefore beneficial to fragment‐based approaches.

### Validation of hits from ^19^F screens

After this initial broad screening, follow‐up screens integrate cheminformatic‐based searches for similar ligands that are commercially available but also cross‐validation of binding to other targets. In fact, some of the fluorine containing ligands show binding to almost all RNAs (e.g. fragment 57). Screening of fragments against RNA targets yielded several hits with a hit rate of up to 26 %. These hits were roughly estimated to bind with an at least low‐millimolar *K*_D_ or tighter to the target RNA. This estimation stems from the observation that most RNA signals did not show large chemical shifts changes in the follow up 2D ^1^H,^15^N‐correlation experiments, upon addition of fragments. This assumption is made, although it is not clear whether changes in chemical shifts are strictly correlated with changes in the chemical environment. After the initial ^19^F screening the hits can be confirmed, *K*_D_ values determined and information on the fragment binding site obtained.

Mapping binding to a specific site in RNAs is usually performed by analysis of chemical shift perturbations (CSPs) that fragment binding causes on the RNA resonances in proximity to the binding epitope. In this approach, care has to be taken to distinguish direct binding induced CSPs from remote effects whose origins are sometimes difficult to assess. Most of the RNAs studied are riboswitches, which bind to metabolites of low molecular weight with an affinity several orders of magnitude higher than the expected affinity of the fluorinated fragments. Thus, orthosterically binding fragments can be detected in a competition experiment. By adding the natural ligand to an RNA sample containing the hit fragment, the natural ligand will compete for the RNA binding site and eventually displace the lower‐affinity binding fragment. Accordingly, if a CPMG‐experiment with long mixing time is recorded, fragment signals will be recovered upon addition of the natural ligand. Although these experiments can provide evidence for ligands that interact with the natural ligand binding site, this might not always be the case, since structural rearrangement upon binding of the natural ligand can also obscure other binding sites, which were formerly accessible to fragments. To evaluate the *T*_2_‐modulated 1D NMR spectra, we measured the fragment signal integrals and calculated the ratios between 200 ms CPMG and 0 ms CPMG applied to ^19^F NMR spectra.

**Figure 3:**
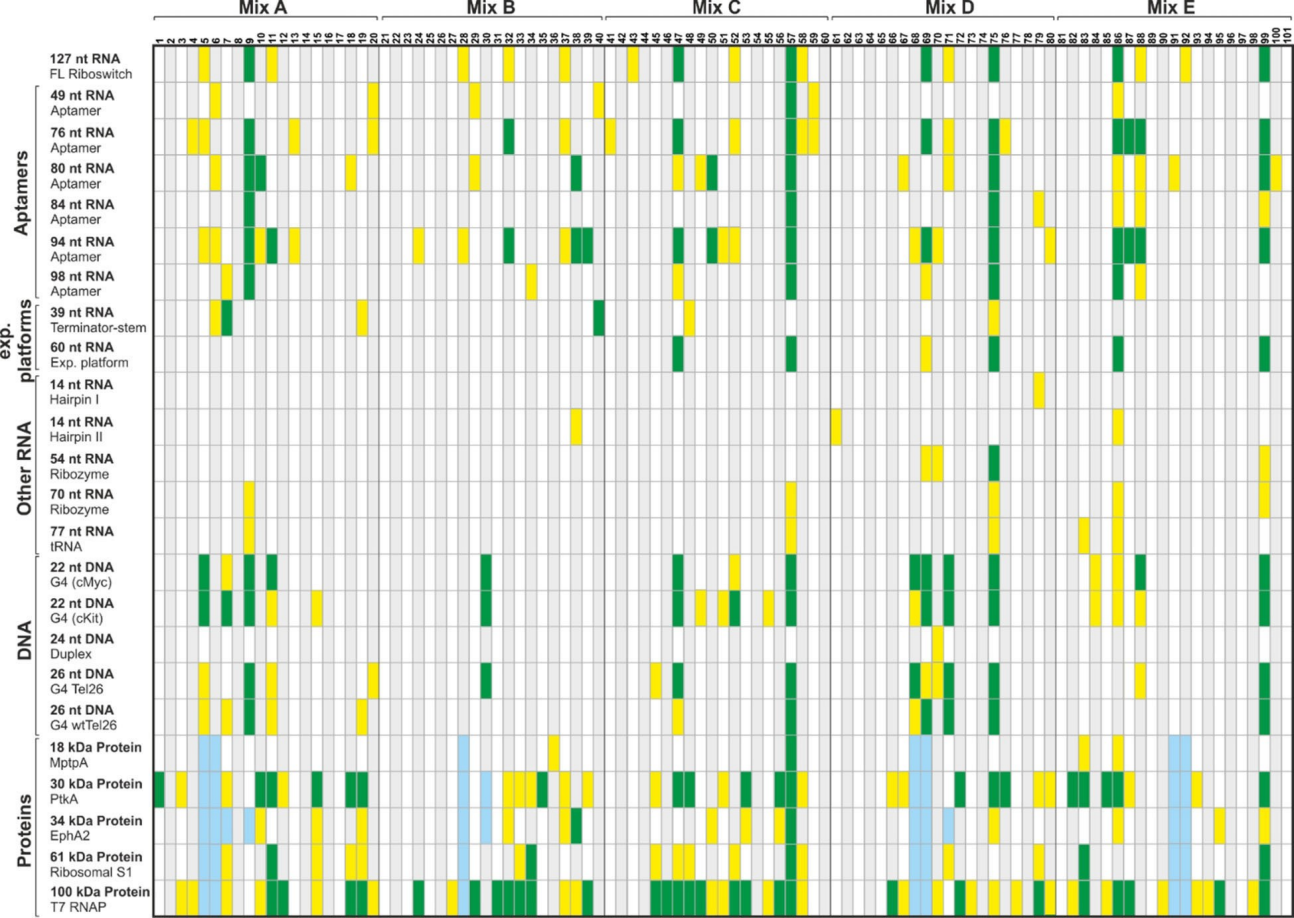
Interaction table of all fragments and biological targets screened. Hits were classified into no binding (*Q* ^bind^>0.67, alternating gray and white), weak (*Q* ^bind^=0.66–0.33, yellow) or strong binding (*Q* ^bind^<0.32, green) in ^19^F CPMG experiments. For protein screens, hits for ∼5 % of the ligands could not unambiguously be assigned (light blue).

**Figure 4:**
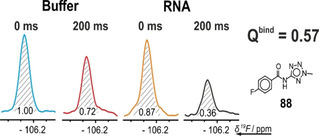
Determination of *Q* ^bind^. Four ^19^F CPMG experiments were recorded to determine the binding factor *Q* ^bind^ from peak integrals as discussed in the main text. The relaxation loss at 200 ms relaxation dephasing time relative to 0 ms dephasing for the ^19^F signal of the ligand was recorded in the presence and absence of biomolecular target.

The quotient *Q* ^bind^ of the integral ratios

$$Q^{bind}=\frac{{Intensity\;Ratio}^{\;+Target}}{{Intensity\;Ratio}^{\;-Target}}$$

with

$$Intensity\;Ratio=\frac{{Peak\;Integral}^{\;CPMG\left(200\;ms\right)}}{{Peak\;Integral\;}^{CPMG\left(0\;ms\right)}}$$

defines a quantifiable factor to classify the ligand‐target interaction into no binding, strong or weak binding Figure 3. The exact effect, however, depends on the overall rotational tumbling time *τ*_c_ that increases with increasing molecular weight. *Q* ^bind^ is a rough criterion, as we did not differentiate between aromatic and aliphatic bound flourines, which we however deem sufficient for the task. The quotient (Peak Integral^+Target^/Peak Integral^−Target^) was automatically calculated; all potential hits were then manually checked. The results are summarized in Figure 4 and spectra regions of all hits are displayed in Figures S5–S39. We obtained hits for all targets. The results show a clear trend that riboswitch RNAs show a high hit rate, ranging from 7 to 26 hits per riboswitch. Riboswitches contain aptamer domains that bind metabolites in the same size range as the fragments or even lower (e. g., F^−^‐sensing^[59]^ or Mg^2+^‐sensing^[60]^ riboswitches). Only one to six hits could be determined for all other RNAs. Although CPMG measurements as relaxation‐based experiments are biased towards increased sensitivity for larger constructs, increased affinities of riboswitch targets are still striking in comparison to the 77 nt tRNA.

DNA structures which were included to sample other nucleic acid structures, namely duplex and G‐quadruplex, showed very different behavior. For the duplex, only a single fragment showed binding. For G‐quadruplexes, we observed between 12 and 20 hits with some overlap to hits that also bind to riboswitch RNAs. Of the five proteins investigated, four showed a large number of hits, ranging from 16 up to 55 (Table 1).

The 18 kDa phosphatase MptpA showed only four hits, in line with the difficult druggability of phosphatases. In general, for 101 fragments screened across 24 different biomolecular targets involving either DNA/RNA/proteins, approximately 5 % of the data were not analyzable. This is a result of the necessity to optimize buffer conditions in particular for proteins. The different buffer conditions can lead to solubility issues and chemical stability issues for this subset of ligands. The remaining fragments show a broad variety of target selectivity from fragments binding exclusively a single target (fragment 100) to highly promiscuous binding behavior (fragment 57).

**Figure 5:**
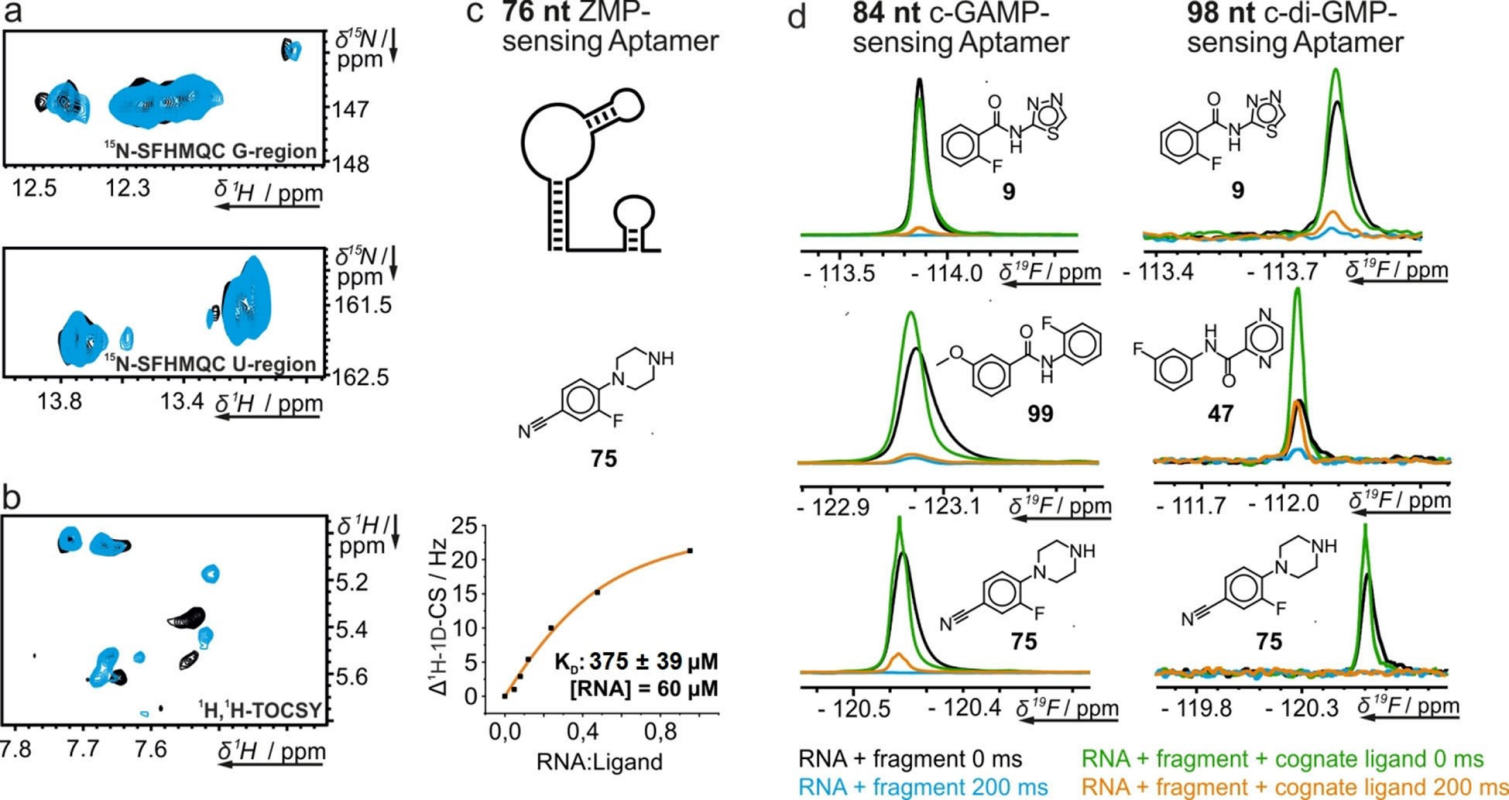
Hit validation and competition experiments. Validation of ^19^F CPMG screening hits for the aptamer domains of the three secondary‐messenger‐sensing riboswitches. a) Spectral regions with signals from guanosine (top) and uridine (bottom) residues of the ^1^H,^15^N correlation experiment of the 76‐nt riboswitch with (blue) and without (black) 75 shown under c. b) ^1^H,^1^H TOCSY spectrum with (blue) and without 75 (black). c) ^19^F 1D NMR titration of 75 with the RNA. *K*_D_ was determined according to Williamson.^[61]^ d) (Partially) competitive binding of fragments to the 84‐ and 98‐nt riboswitch observed in *T*_2_‐modulated 1D ^1^H experiments.

From the pool of the screened biological targets, we chose the aptamer domains containing 76, 84 and 98 nt of the secondary‐messenger‐sensing riboswitches for follow‐up investigation. Weak hits were omitted. To verify hits and rule out effects of fragment mixing we confirmed binding of hits for single fragments. For all investigated fragment and RNA combinations, we were able to observe the same strong effects as in the mixtures. To validate the observed effects and to characterize the binding epitope, we analyzed the effect of fragment 75 addition to the 76 nt riboswitch (Figure 5). We used ^15^N‐isotopically labeled RNA to conduct ^15^N‐correlated 2D spectroscopy on imino hydrogens (Figure 5a) to detect possible chemical shift perturbation of RNA signals introduced by the addition of the screening hit. In these spectra, only helical imino hydrogen signals are visible, which shift distinctly in case of helix groove binding fragments. In our case, we could only observe very small shifts and sometimes additional small signals in the spectra. More pronounced effects could be observed on the signals of aromatic hydrogens in ^1^H,^1^H TOCSY spectra (Figure 5b). Here, clear signal shifts over 10 Hz on H5‐H6 cross peaks of pyrimidine residues were detected.

At concentrations appropriate for screening (in this case 50 μM), and for large RNAs, such as the riboswitches investigated, only the strongest H5‐H6 peaks are visible. In the example shown for compound 75 (Figure 5b), dose‐dependent chemical shift perturbation was detected for three signals. Combined with the data obtained from ^15^N‐correlation spectroscopy, which showed only minor alteration, we can conclude that the respective binding site is located in a flexible region of the RNA.

Additional information on the binding site was obtained from a competitive binding assay. We added the fragment under investigation to the RNA target and characterized the effect of addition of native ligand on the *T*_2_‐modulated signal. As observed earlier, the signal is completely suppressed upon addition of the RNA, but addition of native ligand leads to signal recovery by approximately 15 %. The incomplete recovery of fragment signals points to the possibility that orthosteric binding of the fragment hit to the binding site of the cognate ligand is accompanied by additional nonspecific binding. The major population binds allosterically, and a smaller population binds orthosterically to the same binding site as cognate ligand. We found that signals of other compounds could be recovered by 83 % (compound 47, Figure 5d) after addition of native ligand, pointing to a larger influence on the binding site. The most convenient way to obtain affinity data by NMR is the observation of the ^19^F fragment signal, since it does not require isotopic enrichment and there is only one signal. ^19^F signals in general are very sensitive to changes in their chemical environment and thus, presumably, show also a substantial CSP for the ^19^F upon addition of ligand. In contrast, the chemical shift dispersion of the aromatic hydrogens is smaller and the largest CSP in ^1^H RNA signals was only around 5–8 Hz. From ^19^F CSP data we could obtain affinity constants in the high‐micromolar range, such as 400 μM for fragment 75.^[62]^

The screening of a large number of biomolecular targets demonstrates that the fragment library exhibits the highest hit rate to proteins, followed by RNA and then DNA. RNA hits are observed predominantly for RNAs containing loop regions, bulges, and internal loops. Some of the fragments in the library are promiscuously binding to all three different classes of biomolecular targets. The overlap for hits binding to proteins and RNA is also around 20 %. The most promising result, however, is the observation that each biomolecular target class can be specifically targeted (Figure S4).

### Cheminformatic analysis of hit data

The 69 hit compounds were chemically clustered using Hierarchical Clustering (DistMatrix, Morgan fingerprint, distance threshold 0.6, using Knime software 4.0.2) resulting in 38 singletons and 4 chemical families sharing a closely related scaffold. The largest cluster contains 5 members that were binders for proteins, DNA/RNA, and DNA/RNA/proteins targets. No cluster seems to be specific to any of the target families screened.

In order to check if there are any correlations between chemical structures and the number of targets that bind to it, we generated a number of molecular descriptors linked to the shape, electrostatic and hydrophobic interactions. The correlation matrix of all data shows no significant correlations between the number of target hits and molecular descriptors. The lowest and the highest correlations found are respectively with the number of aromatic atoms (*R*=+0.27) and SP3 descriptor (sp^3^ carbon atom count/total carbon atom count reflecting the flatness of the molecules; *R*=−0.23).

**Figure 6:**
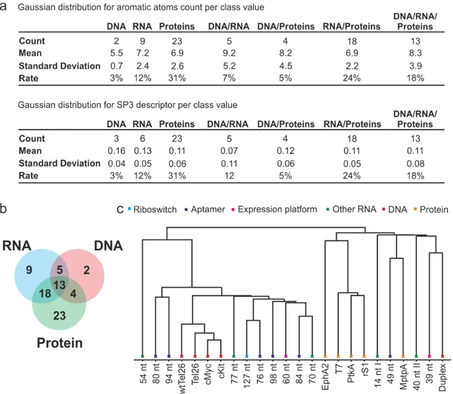
Cheminformatic analysis of hit data for all RNA, DNA and protein biomolecules. a) Gaussian distributions for aromatic atoms and SP3 descriptor over categories of biomolecules. SP3 descriptor (sp^3^ carbon atom count/total carbon atom count) reflects the flatness of the fragment molecules. b) Visualization of categories in a Venn diagram. c) Euclidian distribution of hits to the target biomolecules.

The statistical analysis of the number of aromatic atoms for each category of binders shows higher average value of this descriptor for compounds hitting DNA/RNA/proteins (9.2) after the DNA/RNA binders (10.4) but in this last case the number of hits is too small to draw a conclusion. The SP3, like the other molecular descriptors, shows no significant difference between the category for hits (Figure 6). The substructure counting of popular and frequent motifs in organic molecules reported in the Table 6a, also shows no significant enrichment in different categories of binders. Because of the small size of the fragments in the current ^19^F‐library, there is no privileged class of compounds or relevant physicochemical properties that can be specific to a family of biological targets (RNA/DNA/proteins). The ^19^F‐fragment library is therefore suitable for RNA, DNA, and proteins and can be used to generate starting points for follow‐up screens and to examine the presence of potential binding pockets for ligands in the individual targets. Moreover, correlation analysis (Figure 6c and Figure S4) shows striking clustering of hits between riboswitches and aptamers, DNA and proteins, respectively. Currently research focuses on the development of libraries solely suited for RNA, taking the repetitive nature of the RNA backbone, the higher charges and RNA dynamics into account.[^63^, ^64^] While the here used library of ^19^F‐fragments was shown to be suited for proteins and RNA likewise, these recent developments could be taken into account in order to enrich current fragment libraries into a more RNA‐focused library.

### Follow‐up chemistry

After hit identification, the next major aspect in fragment‐based drug discovery is to link initial hits to increase binding affinity. Such an improvement can be achieved by linkage of the fragment to an RNA binder, as shown for a neomycin‐acridine conjugate.^[65]^ To generate affinity we used an acridine moiety, which is a well‐known intercalator that also allows fast follow‐up chemistry.[^66^, ^67^] Herein we describe a three step synthesis to generate an acridine moiety followed by linking it to a fragment. We chose a fragment from an additional ^1^H screening for the 39‐nt terminator stem (P2D11) with a similar structure to the hits from ^19^F screening because of better availability and facilitated synthesis. On linking this fragment to the acridine, a binding in low‐micromolar range was observed as described in the Supporting Information.

## Conclusion

In summary, we outline the screening of ^19^F‐containing libraries to 14 different RNA targets. Commercially available fragment library can be used to identify low‐molecular‐weight fragments. We also show the general versatility of the used poised library, allowing straight‐forward follow‐up chemistry to increase binding affinity to as low as 1 μM, while observing a 15‐fold selectivity between different RNA targets. Identified hits report on the general druggability of RNA targets.^[68]^ Cheminformatics allow delineation of features within the fragments that are specific for each class of biomolecular target. Our study will aid our current effort to identify new ligands with antiviral activity in the context of combatting the COVID‐19‐pandemic (Covid19‐nmr.de).

## Experimental Section

**Initial checks**: All fragments in the library utilized for screening were checked for inconsistencies by running 1D ^1^H and ^19^F spectra of each compound. Spectra were analyzed by hand, and compounds showing wrong or additional signals were ruled out.

**RNA preparation**: All RNAs were prepared in house through in vitro transcription with T7 RNAP.^[69]^ DNA templates included the necessary T7 promotor and were either obtained from linearizing plasmid containing the sequence of interest or PCR run‐off. Transcription conditions were optimized for yield and sample purity and in vitro transcription was performed in 10 to 20 mL scale dependent on the expected yield. Purification was performed either by HPLC, preparative PAGE or buffer exchange to NMR buffer if necessary.^[70]^ Concentration and purity of the samples were analyzed by UV/Vis spectroscopy and analytical PAGE respectively. For some of the follow‐up experiments uniformly ^15^N‐labeled RNA was prepared with the same procedure using isotopically ^15^N‐labeled rNTPs.

**Sample generation**: Each fragment mixture contained 20 or 21 fragments at 2.5 mM concentration in 90 % [D_6_]DMSO with 10 % D_2_O. Mixtures were designed (minimize signal overlap) in an excel sheet, using the chemical shift obtained from individual compound measurement. SamplePro Tube robot was used for automated pipetting of the samples into the NMR tubes. The final sample volume was 170 μL with 5 % D_2_O as locking solvent. For each target, two samples (with and without target) were prepared per mixture. ^19^F screening was performed at a ratio of 1 : 1 with respect to RNA and fragments. The final [RNA]([protein])‐ligand concentration was around 50 μM. The screening buffer was 25 mM KPi, pH 6.2, 50 mM KCl, 5 mM MgCl_2_ in 94 % H_2_O/5 % D_2_O/1 % [D_6_]DMSO in a 3 mm tube. For DNA, buffer conditions were 25 mM KPi, pH 7.0, 70 mM KCl, in 94 % H H_2_O/5 % D_2_O/1 % [D_6_]DMSO. For proteins MptpA and PtkA buffer conditions were 25 mM HEPES/NaOH, pH 7.0, 150 mM NaCl, 10 mM DTT and 50 mM HEPES/NaOH, pH 7.5, 300 mM NaCl, 10 mM DTT, 10 mM MgCl_2_ respectively. For EphA2 buffer conditions were 20 mM Tris pH 8, 200 mM NaCl, 5 mM MgCl_2_, 3 mM TCEP. For T7 and rS1 buffer conditions were 25 mM KPi (pH 7.2), 150 mM KCl, 5 mM DTT. NMR screening data of ^19^F 1D and ^19^F CPMG *T*_2_ measurements were recorded with mixing times of 0, 200, and 400 ms for the CPMG experiments. Strong hits from screening of three investigated RNAs were chosen for follow‐up experiments. Samples of these fragments with the respective RNA were prepared in the same way omitting mixture generation, to confirm binding of the single fragment. In the same way, samples for the competition experiments were generated. Native ligand was added at an equimolar concentration to the RNA. Additionally, samples of ^15^N‐labeled RNA were prepared with an RNA concentration between 50 and 100 μM and fragment concentration of 1.25 mM and were used in follow‐up experiments. Reference samples contained no fragment.

**NMR spectroscopy**: Spectra acquisition was carried out on a Bruker AVIIIHD‐600 NMR spectrometer equipped with a five mm ^1^H/^19^F [^13^C,^15^N]‐TCI prodigy cryo‐probe and high throughput sample changer for 579 samples with temperature option for sample storage. For the screening process the following spectra were acquired: ^19^F 1D, water‐suppressed proton 1D and ^19^F 1D with CPMG spinlock (0, 200 and 400 ms). All ^19^F spectra were recorded at room temperature, without ^1^H decoupling and processed with line broadening function of 10 Hz. The CPMG spin lock was applied using an adiabatic WURST pulse with a bandwidth of 120 ppm and a length of 2 ms. The pulse is calculated on‐the‐fly by wavemaker software in Topspin. The interpulse delay of the CPMG spin lock was set to 9 ms. Screening data were analyzed by integration with Topspin 4.0 (Bruker Biospin) and manually checked using the integrated fragment‐based screening software tool. Strong hits for three RNAs investigated were chosen for follow‐up experiments. ^19^F 1D CPMG spectra were run on single fragment samples with the same parameters as in screening. The following spectra were acquired: ^15^N SFHMQC, proton 1Ds with excitation sculpting^[71]^ or jump‐and‐return echo^[72]^ scheme for water suppression, and ^1^H,^1^H TOCSY with excitation sculpting.

**Synthesis**: All experimental details for the synthesis and characterization of compound **1** are described in the Supporting Information.

## Conflict of interest

The authors declare no conflict of interest.

## Supporting information

As a service to our authors and readers, this journal provides supporting information supplied by the authors. Such materials are peer reviewed and may be re‐organized for online delivery, but are not copy‐edited or typeset. Technical support issues arising from supporting information (other than missing files) should be addressed to the authors.

Supplementary

Supplementary
