# Supplementary material for: 19F NMR‐Based Fragment Screening for 14 Different Biologically Active RNAs and 10 DNA and Protein Counter‐Screens
Source: Chembiochem. 2020 Sep 25;22(2):423–33. doi: 10.1002/cbic.202000476 (PMC7436455; doi:10.1002/cbic.202000476)
Supplement: Supplementary file 2 — Supplementary [file CBIC-22-423-s002.pdf]

ChemBioChem

Supporting Information

^{19}F NMR-Based Fragment Screening for 14 Different Biologically Active RNAs and 10 DNA and Protein Counter-Screens

Oliver Binas[+], Vanessa de Jesus[+], Tom Landgraf[+], Albrecht Eduard Völklein[+], Jason Martins, Daniel Hymon, Jasleen Kaur Bains, Hannes Berg, Thomas Biedenbänder, Boris Fürtig, Santosh Lakshmi Gande, Anna Niesteruk, Andreas Oxenfarth, Nusrat Shahin Qureshi, Tatjana Schamber, Robbin Schnieders, Alix Tröster, Anna Wacker, Julia Wirmer-Bartoschek, Maria Alexandra Wirtz Martin, Elke Stirnal, Kamal Azzaoui, Christian Richter,* Sridhar Sreeramulu,* Marcel Jules José Blommers,* and Harald Schwalbe*

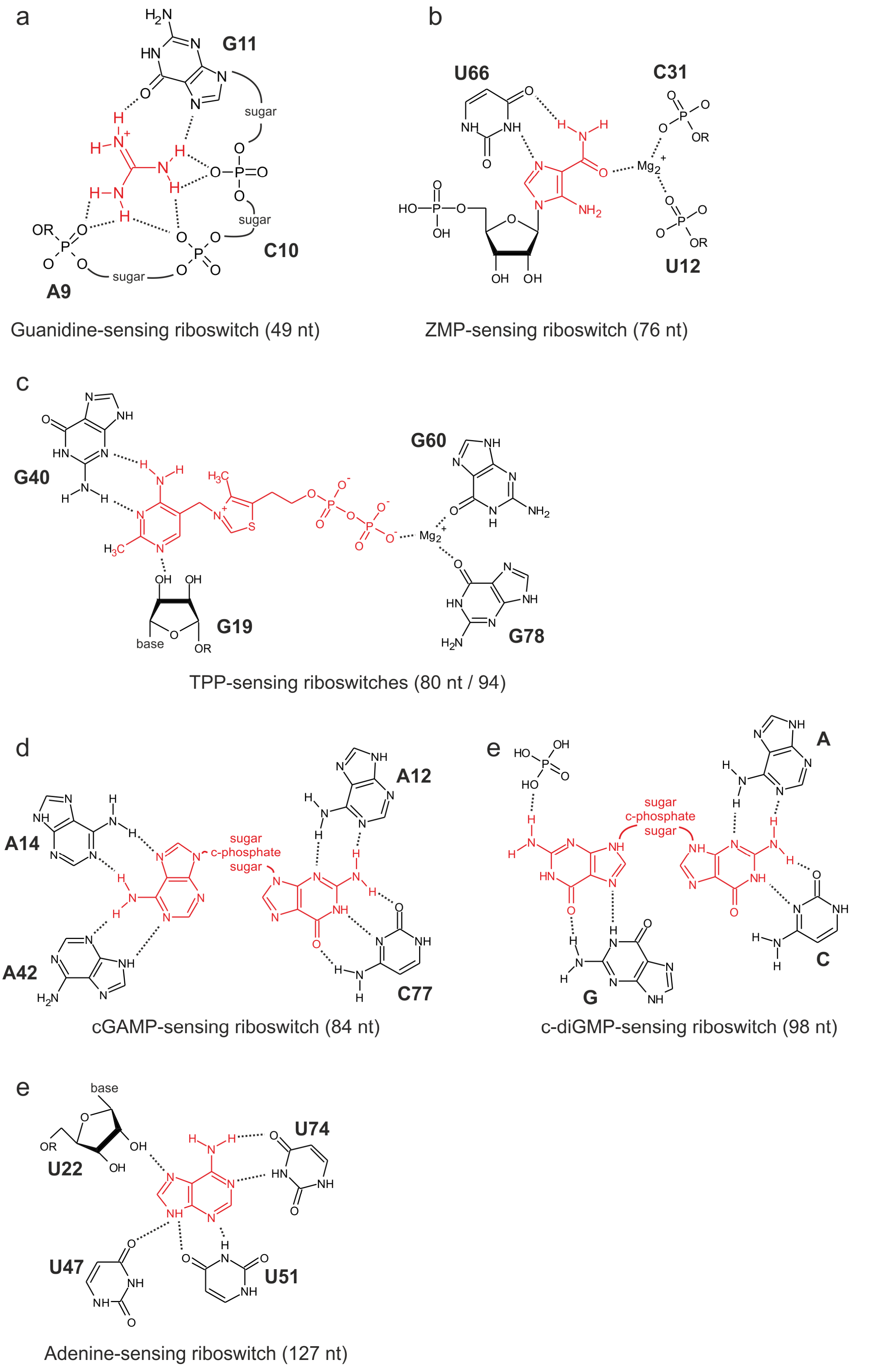

Supplementary Figure 1: Molecular recognition of ligands (red) by riboswitches used in ^{19}F-screening.(A) Guanidine-sensing riboswitch, (B) ZMP-sensing riboswitch ,(C) TPP-sensing riboswitches, (D) cGAMP-sensing riboswitch, (E) c-diGMP-sensing riboswitch,(F) Adenine-sensing riboswitch.

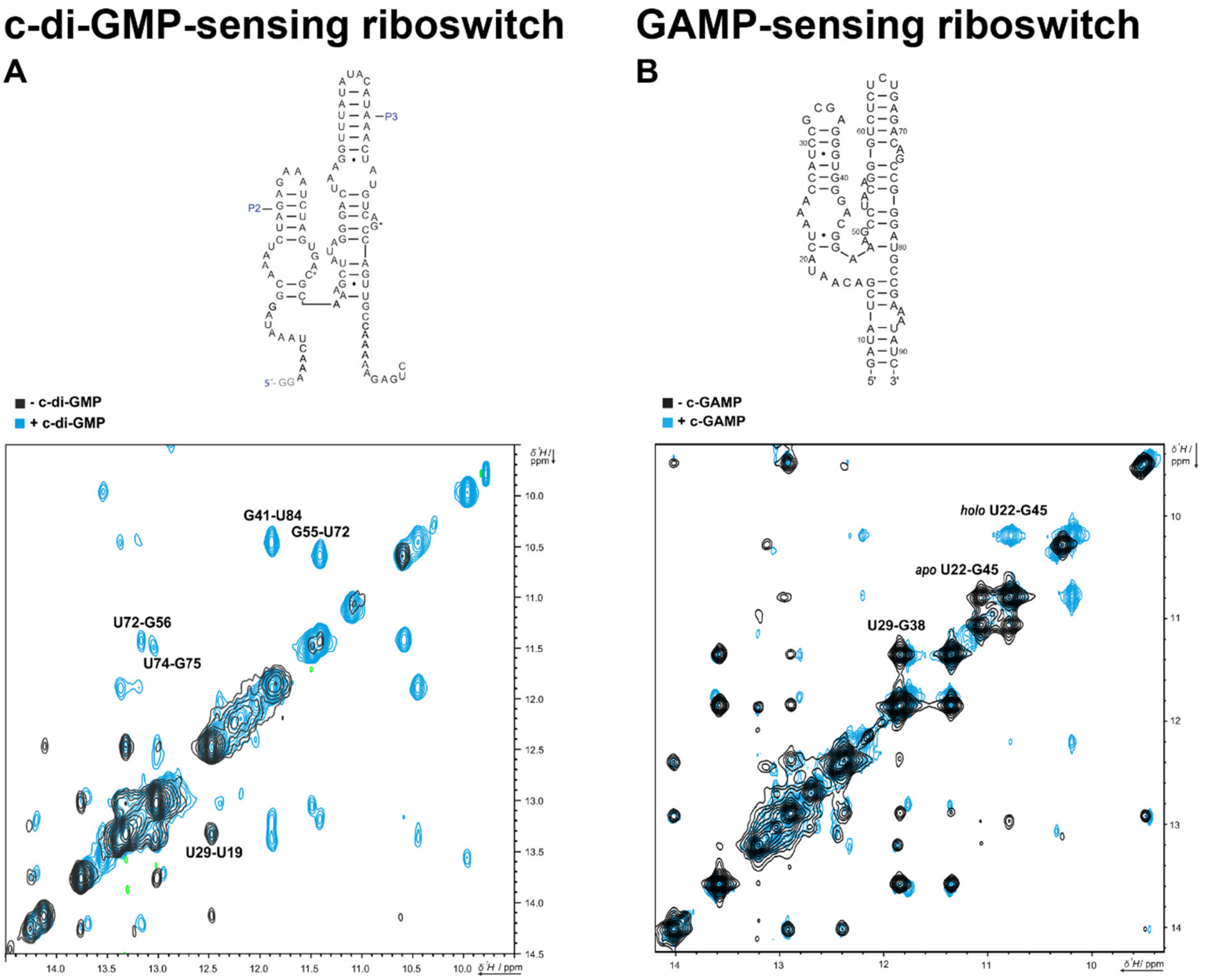

Supplementary Figure 2: (A) [^{1}H, ^{1}H]-NOESY spectra of the 98 nt c-di-GMP-sensing Cd1 riboswitch from Clostridium difficile in the apo and holo conformation. The secondary structure of the apo conformation is shown. Spectra were recorded in 50 mM Bis-Tris buffer with 120 mM NaCl and 20 mM $MgCl_2$ at 298 K, 600 MHz and pH 6.1. Spectra were recorded with a spectral width of 24 ppm and 16 ppm in the indirect dimension. Spectra were recorded with 2048 points in the direct and 380 points in the indirect dimension and an acquisition time of 71 ms. The spectrum without c-di-GMP was recorded with 384 scans and the spectrum with c-di-GMP with 364. The sample concentration was 500 µM. (B) [^{1}H, ^{1}H]-NOESY spectra of the pilM 3',3'-cGAMP-sensing riboswitch (84 nt) from Geobacter metallireducens in the apo and holo conformation. The secondary structure of the apo conformation is shown. The spectra were recorded in 25 mM potassium phosphate buffer (pH 6.2), 50 mM KCl, 5 mM $MgCl_2$ and 10% D_2O. DSS was used as reference. The mixing time was 100 ms. The spectra were recorded at 308 K. The apo spectrum was recorded at an RNA concentration of 1.6 mM at 700 MHz with 256 scans. The spectrum was recorded with a spectral width of 24 ppm in the direct and 15 ppm in the indirect dimension and an acquisition time of 63 ms. The apo spectrum was recorded with 3172 points in the direct and 488 points in the indirect dimension. The holo spectrum was recorded at an RNA concentration of 1.1 mM with 1.6 mM cGAMP at 950 MHz with 312 scans. The spectrum was recorded with a spectral width of 24 ppm in the direct and 15.5 ppm in the indirect dimension and an acquisition time of 64 ms. Spectrum was recorded with 2914 points in the direct and 384 points in the indirect dimension.

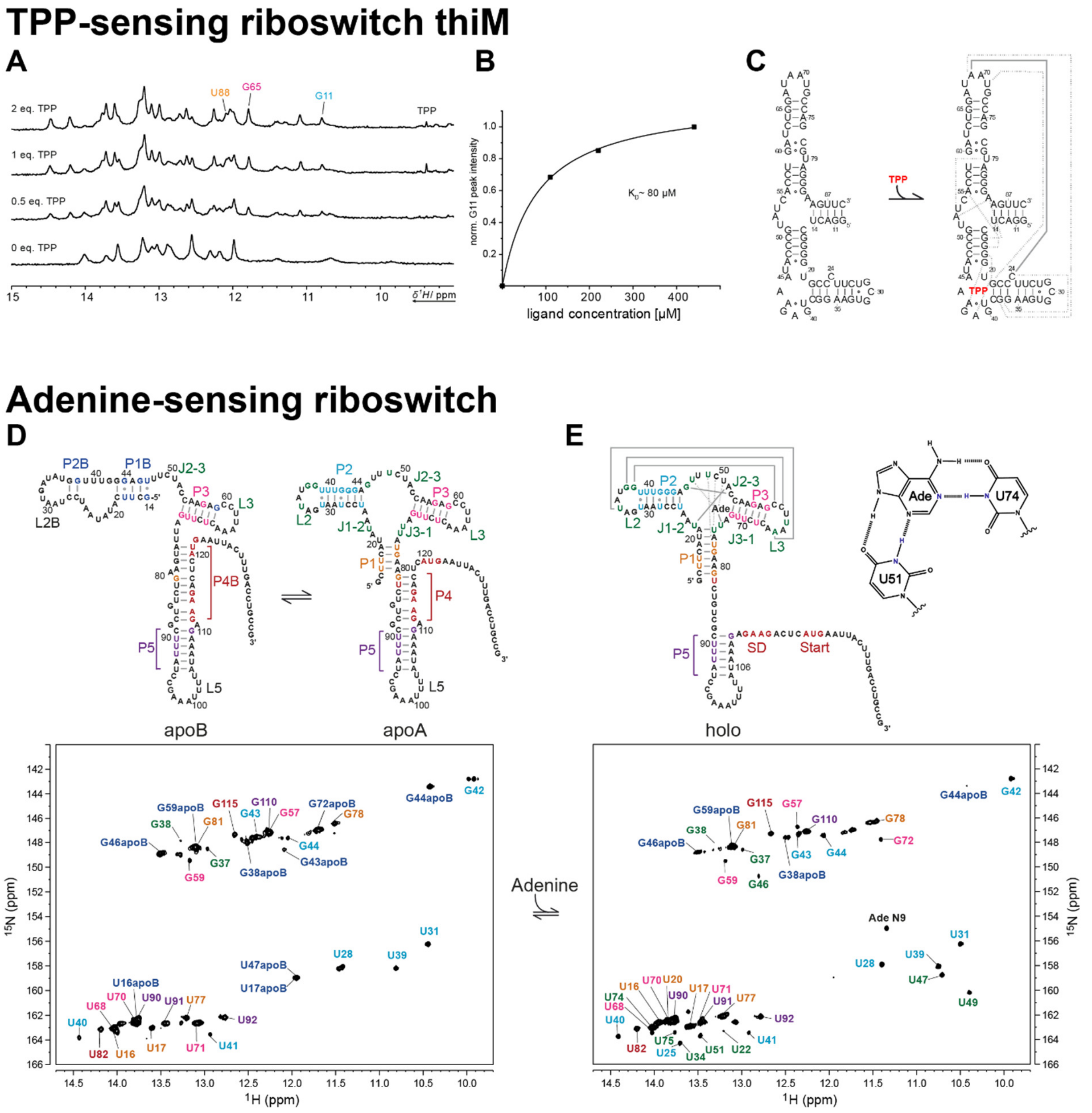

Supplementary Figure 3: (A) Imino proton region of 1D ^{1}H spectra of u-[^{13}C, ^{15}N] labeled thiM RNA (80 nt) with 5 mM Mg^{2+} at increasing TPP concentrations. Spectra were recorded at a 700 HD MHz Bruker spectrometer at 298 K. All spectra were recorded with 8 scans. The spectral width was 24 ppm. Sample had an RNA concentration of 220 µM in NMR buffer (25 mM potassium phosphate buffer, pH 6.2, 10% D_2O, 100 µM DSS) (B) K_D value determination of TPP ligand binding to thiM RNA. The intensities of the G11 peak in the titration shown in (C) were normalized to the highest value and fitted using a hyperbolic one-site binding curve. (C) Secondary structure of thiM RNA (80 nt). (D) ^{1}H-^{15}N-BEST TROSY spectra of 127 nt adenine-sensing riboswitch from Vibrio vulnificus in apo and holo conformation. Spectra were recorded in potassium phosphate buffer (25 mM KPi, 150 mM KCl and 5 mM $MgCl_2$, pH 7.2) at 800 MHz. The proton carrier frequency was set to the resonance frequency of the solvent (4.7 ppm). The interscan delay was set to 0.4 seconds. The spectra were recorded with a spectral width of 24 ppm in the direct dimension and 30 ppm in the indirect dimension. The spectra were recorded with an acquisition time of 103 ms representing 3998 points in the ^{1}H dimension. In the indirect dimension, 256 points were recorded with an acquisition time of 52 ms. (A) The apo spectrum was recorded with 4 scans at an RNA concentration of 465 µM. (E) The holo spectrum was recorded with 8 scans at an RNA concentration of 229 µM.

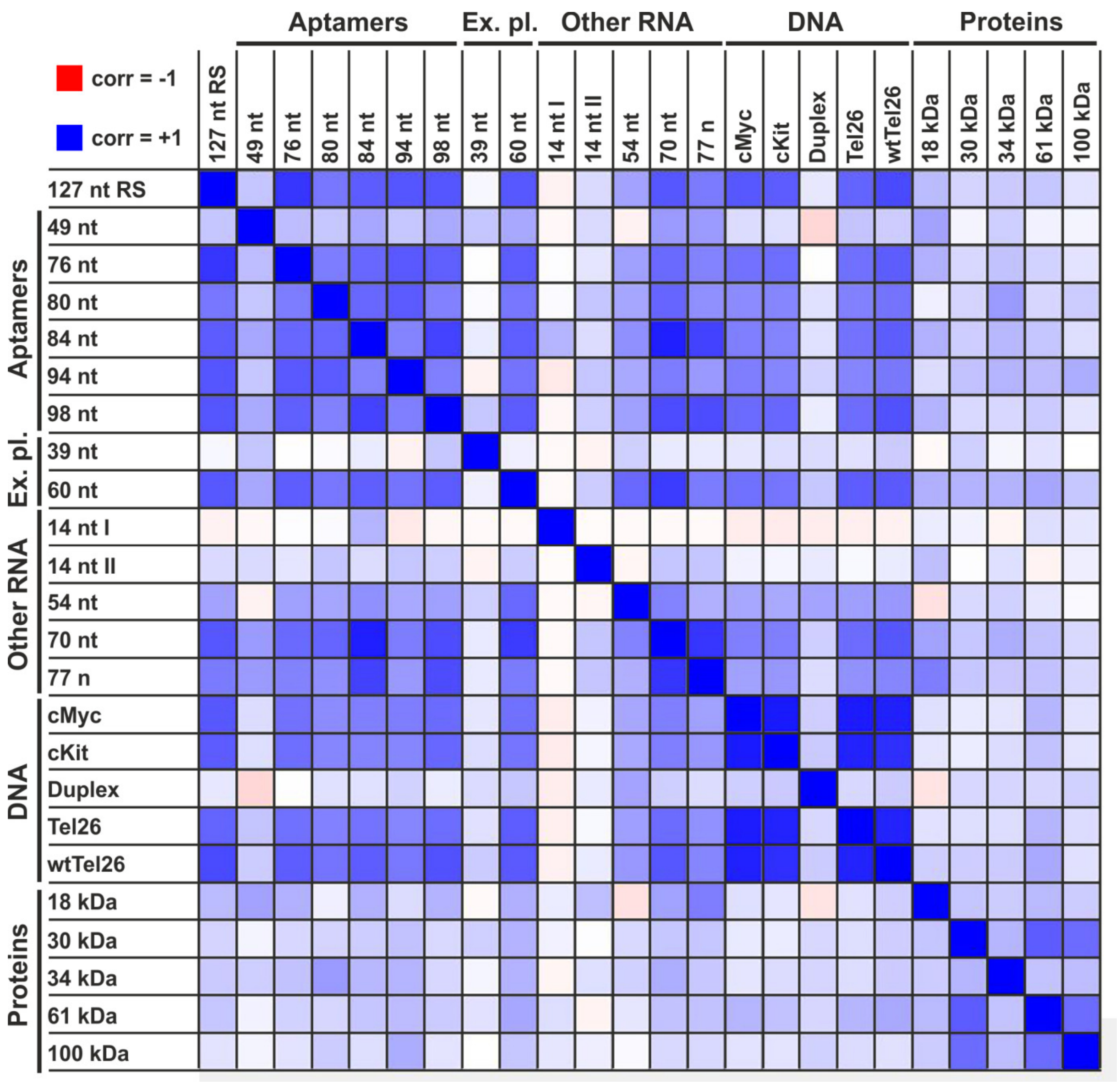

Supplementary Figure 4: Correlation matrix of hit clusters, displaying hit correlation between different targets screened by ^{19}F-FBS.

^{19}F Library

Supplementary Table 1: ^{19}F library for the ^{19}F-NMR-based fragment screening.

	SMILES code	Formula	Chemical structure
**1.**	FC(F)(F)c1ccc(cc1)CN2CCOCC2	C12H14F3NO	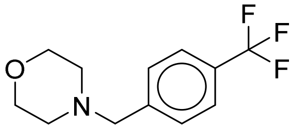
**2.**	Cl.O=C(CC(F)(F)F)N1CCNCC1	C7H12ClF3N2O	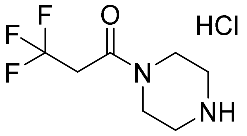
**3.**	Clc1cc(NC(=O)C(F)(F)F)ccc1	C8H5ClF3NO	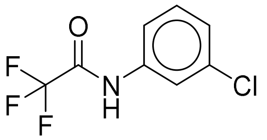
**4.**	Fc1cc(C)cc(c1)S(=O)(N)=O	C7H8FNO2S	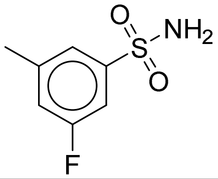
**5.**	Fc1cccc(c1)NC(=O)COc2ccccc2	C14H12FNO2	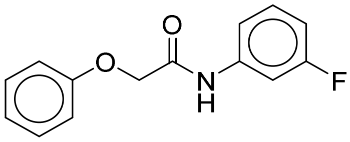
**6.**	Fc2cccc(CNCc1ccco1)c2	C12H12FNO	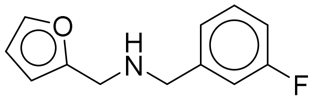
**7.**	Fc2cc(CN1CCOCC1)ccc2	C11H14FNO	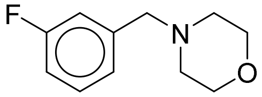
**8.**	O=S2(=O)CCCN2Cc1cccc(F)c1	C10H12FNO2S	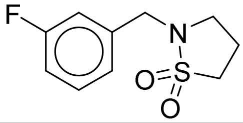
**9.**	O=C(Nc1nncs1)c2ccccc2F	C9H6FN3OS	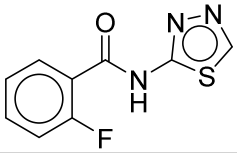
**10.**	Clc1cccc(F)c1CNC(=O)NC	C9H10ClFN2O	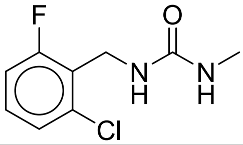
**11.**	COc2ccc(NCc1ccc(F)cc1)cc2	C14H14FNO	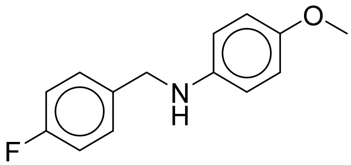
**12.**	Cc1ccc(CS(N)(=O)=O)cc1F	C8H10FNO2S	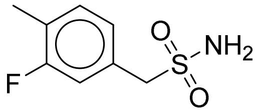

**13.**	O=C(Nc1ccc(F)cc1)N2CCN(C)CC2	C12H16FN3O	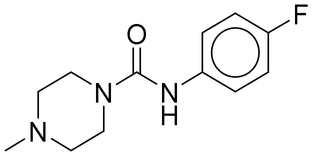
**14.**	O=C(Nc1ccc(F)cc1F)Nn2cnnc2	C9H7F2N5O	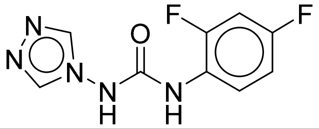
**15.**	COC(=O)N1CCN(CC1)c2ccc(F)cc2	C12H15FN2O2	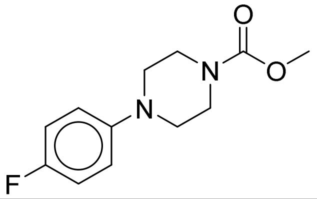
**16.**	Fc1cccnc1N(C)[C@@H](C)CO	C9H13FN2O	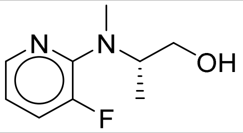
**17.**	CCOc1c(cccc1F)C(=O)N(C)C	C11H14FNO2	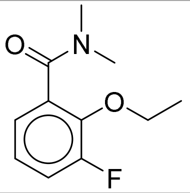
**18.**	Fc2ccccc2NCc1ccnn1	C10H10FN3	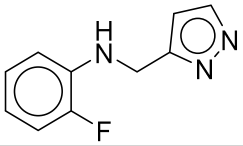
**19.**	CC(=O)N2CC(C)Oc1c(F)cccc12	C11H12FNO2	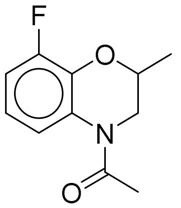
**20.**	Fc1ccc(nc1)NC2CCOCC2	C10H13FN2O	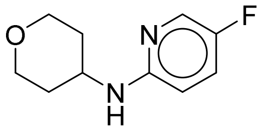
**21.**	FC(F)(F)Oc1ccccc1C(=O)O	C8H5F3O3	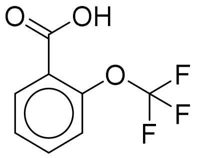
**22.**	O=C(Cn1ccc(n1)C(F)(F)F)NC(C)(C)C	C10H14F3N3O	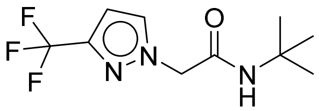
**23.**	CC1(C)CN(CCO1)C(=O)CC(F)(F)F	C9H14F3NO2	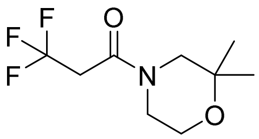

**24.**	FC(F)(F)c1ncc(cn1)C(N)=O	C6H4F3N3O	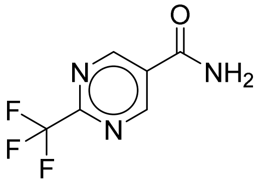
**25.**	O=C(Nc1cc(ccc1)C(C)=O)C(F)(F)F	C10H8F3NO2	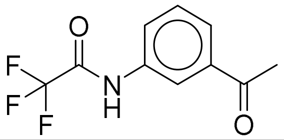
**26.**	Cl.Cl.Cl.FC(F)n2ccnc2CN1CCNCC1	C9H17Cl3F2N4	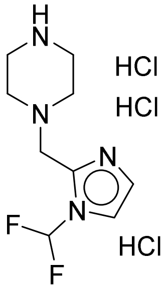
**27.**	O=C(c1ccc(F)cc1Br)N(C)C	C9H9BrFNO	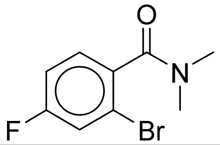
**28.**	Fc2ccc(CNCc1ccc(C#N)cc1)cc2	C15H13FN2	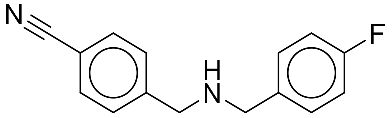
**29.**	FC=1CN(CCC=1)Cc2nnc(C)s2	C9H12FN3S	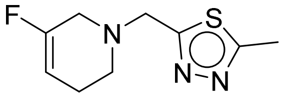
**30.**	O=C(c1c(F)cccc1F)N2CCCCCC2	C13H15F2NO	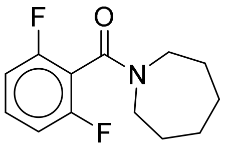
**31.**	FC=1CN(CCC=1)S(=O)(=O)C2(C)CC2	C9H14FNO2S	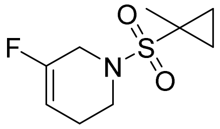
**32.**	O=C(N)c1ccc(C)cc1F	C8H8FNO	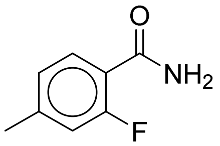
**33.**	O=C(C)Nc1cccc(F)c1C(=O)O	C9H8FNO3	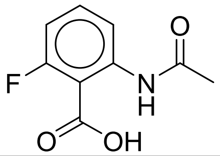
**34.**	O=C(Nc1ccc(F)cc1)Cc2ccc(F)cc2	C14H11F2NO	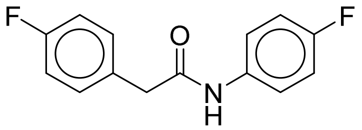
**35.**	O=C(Nc1ccc(F)cc1F)CC(C)(C)C	C12H15F2NO	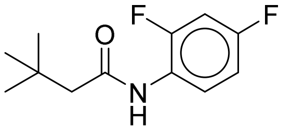

**36.**	Fc2ccccc2CNc1cnnc1	$C_{10}H_{10}FN_3$	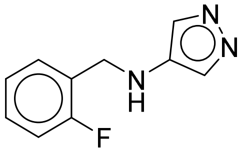
**37.**	Fc1cc(ccc1N(C)C)C(=O)NCC	$C_{11}H_{15}FN_2O$	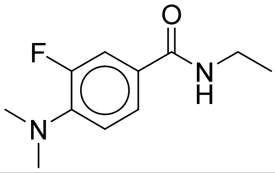
**38.**	Cc2ncc(CNc1cc(F)cnc1)s2	$C_{10}H_{10}FN_3S$	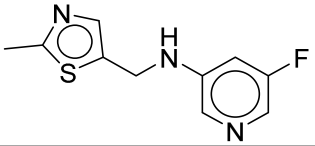
**39.**	Fc1cc(ccc1NCC)S(=O)(C)=O	$C_9H_{12}FNO_2S$	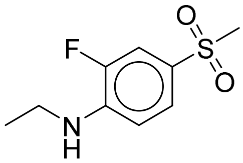
**40.**	Fc2cccnc2NCC1CCOCC1	$C_{11}H_{15}FN_2O$	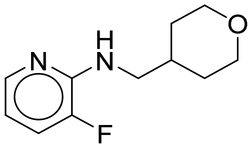
**41.**	Clc1cc(cnc1NC)C(F)(F)F	$C_7H_6ClF_3N_2$	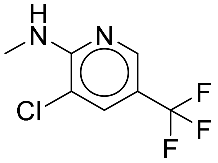
**42.**	CC1CCN(CC1)C(=O)CC(F)(F)F	$C_9H_{14}F_3NO$	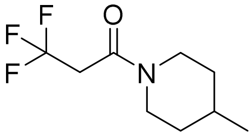
**43.**	O=C(Nc1ccc(cc1)C(=O)OC)C(F)(F)F	$C_{10}H_8F_3NO_3$	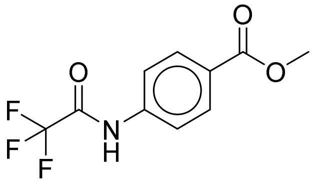
**44.**	FC1(F)CCC(CC1)C(N)=O	$C_7H_{11}F_2NO$	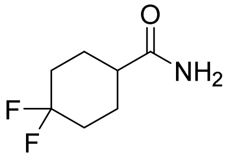
**45.**	Fc1ccc(cc1)Cn3cnc2ccccc23	$C_{14}H_{11}FN_2$	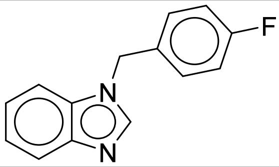
**46.**	CNC(=O)c2c(C)onc2c1ccc(F)cc1	$C_{12}H_{11}FN_2O_2$	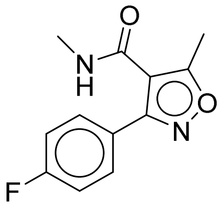

**47.**	O=C(Nc1cccc(F)c1)c2cnccn2	C11H8FN3O	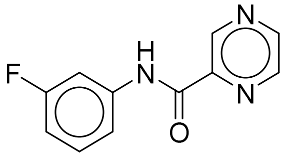
**48.**	O=C(NCc1ccccc1)c2cc(F)ccc2	C14H12FNO	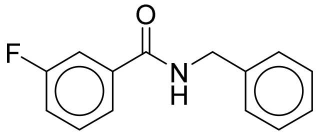
**49.**	Fc1cc(C)c(CS(N)(=O)=O)cc1	C8H10FNO2S	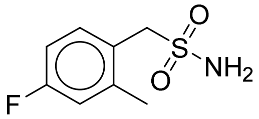
**50.**	FC(F)c1nn(C)cc1C(N)=O	C6H7F2N3O	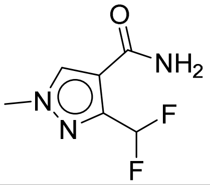
**51.**	Fc1ccccc1C(=O)NCCc2ccncc2	C14H13FN2O	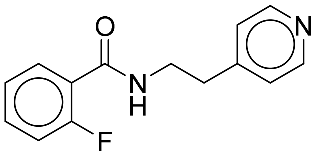
**52.**	O=C(Nc1ccc(F)cc1)CN2CCCC2	C12H15FN2O	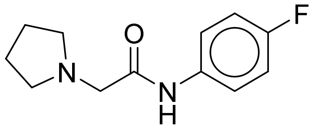
**53.**	O=C(NCCc1ccc(F)cc1)c2ccco2	C13H12FNO2	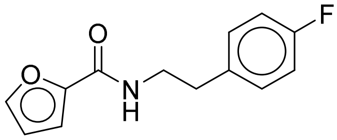
**54.**	O=C(C)Nc1cc(c(F)cc1)C(=O)O	C9H8FNO3	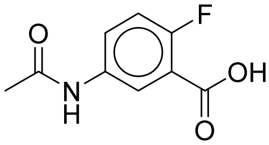
**55.**	Cl.Fc1ccccc1N2CCC(NC)C2=O	C11H14ClFN2O	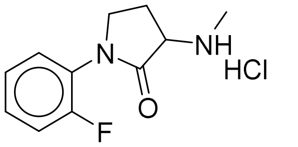
**56.**	O=C(C1CC1)N2CCN(CC2)c3ccc(F)cc3	C14H17FN2O	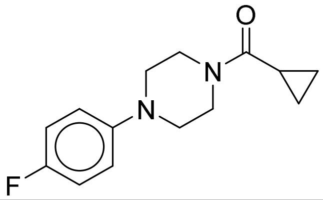
**57.**	COc1ccc(cc1)C(=O)Nc2ccccc2F	C14H12FNO2	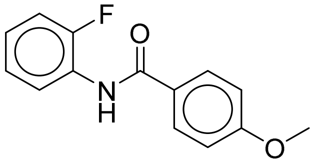

**58.**	CC(=O)NCCc2cnc1ccc(F)cc12	C12H13FN2O	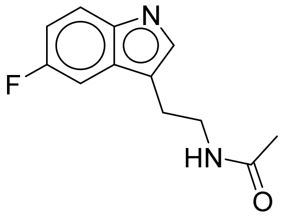
**59.**	CCN2CCC(Nc1ccccc1F)CC2	C13H19FN2	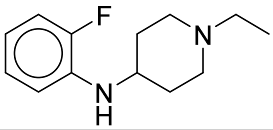
**60.**	O=C(N1CCN(C)CC1)c2ccc(F)c(F)c2	C12H14F2N2O	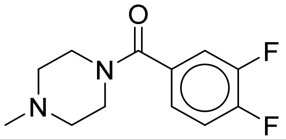
**61.**	FC(F)(F)Oc1ccccc1NC(N)=S	C8H7F3N2OS	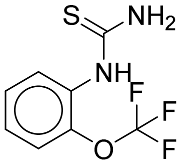
**62.**	FC(F)(F)C=1CC(=O)N(CC)N=1	C6H7F3N2O	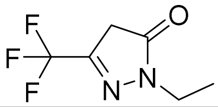
**63.**	Fc2ccc(CNCc1ccccc1)cc2	C14H14FN	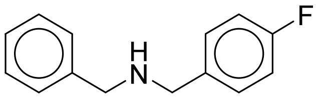
**64.**	O=C(Nc1nnc(CC)s1)C(F)(F)F	C6H6F3N3OS	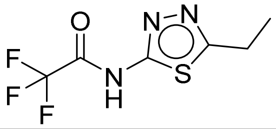
**65.**	FC(F)OCC(=O)N2CCCC1(CC1)C2	C10H15F2NO2	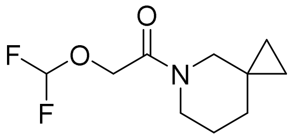
**66.**	Fc1c(C)cccc1S(=O)(=O)NC	C8H10FNO2S	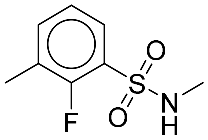
**67.**	CC(O)COc1cccc(F)c1	C9H11FO2	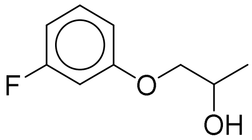
**68.**	Cl.N[C@@H]1CCCN(C1)C(=O)CCC(F)(F)F	C9H16ClF3N2O	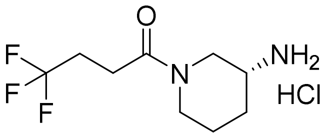
**69.**	Cc2ccc(NC(=O)c1cccc(F)c1)cc2O	C14H12FNO2	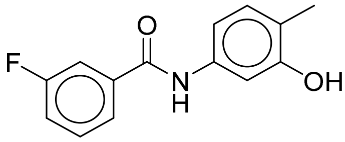
**70.**	O=C(N1CC(F)=CCC1)[C@H]2CC[C@@H](C)O2	C11H16FNO2	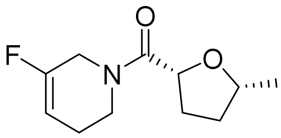

**71.**	Fc2ccccc2CN1CCOCC1C	C12H16FNO	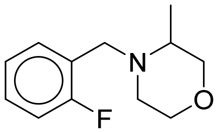
**72.**	O=C(NC1CCCCC1)Cc2ccc(F)cc2	C14H18FNO	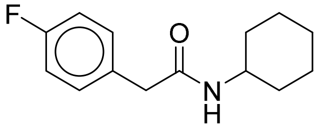
**73.**	Fc1ccc(CCNS(C)(=O)=O)cc1	C9H12FNO2S	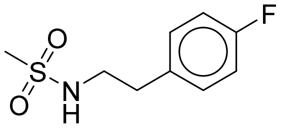
**74.**	O=C(NCc1ccco1)Nc2ccc(F)cc2	C12H11FN2O2	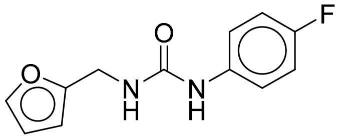
**75.**	N#Cc1cc(F)c(cc1)N2CCNCC2	C11H12FN3	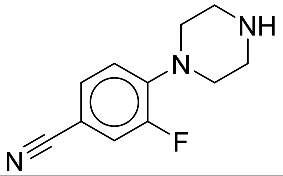
**76.**	O=C(N)N1CCN(CC1)c2ccc(F)cc2	C11H14FN3O	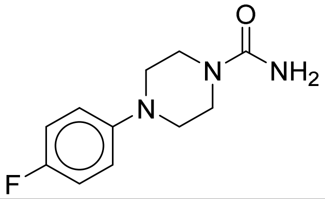
**77.**	O=C(COc1ccc(F)cc1)N2CCCC2	C12H14FNO2	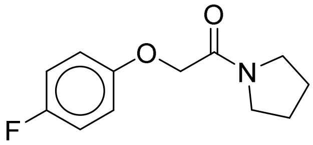
**78.**	O=S(=O)(Nc1ccccc1F)CC	C8H10FNO2S	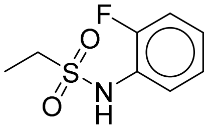
**79.**	Fc1cccnc1N2CC(C)(C)C(O)C2	C11H15FN2O	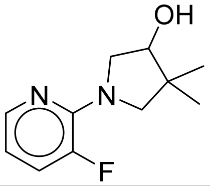
**80.**	C[C@@H](CO)Nc1ncc(Cl)cc1F	C8H10ClFN2O	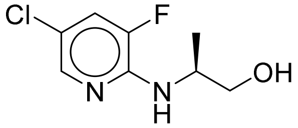
**81.**	FC(F)(F)Oc1ccccc1CN	C8H8F3NO	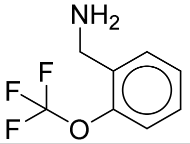
**82.**	FC(F)(F)Oc1ccc(cc1)NC(N)=S	C8H7F3N2OS	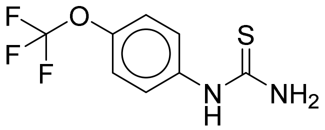

**83.**	FC(F)(F)c1cc(N)c(cc1)N2CCCCC2	C12H15F3N2	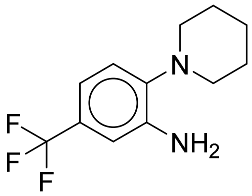
**84.**	Cl.FC(F)(F)c1cc(NC(=O)C(C)N)nn1	C7H10ClF3N4O	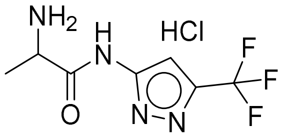
**85.**	CNCc1ccc(cc1)C(F)(F)F	C9H10F3N	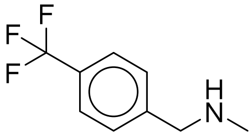
**86.**	O=C(Nc1ccc(F)cc1)Nc2ccc(F)cc2	C13H10F2N2O	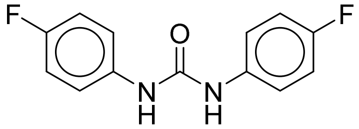
**87.**	FC(F)Oc1ccc(cc1)C(C)=O	C9H8F2O2	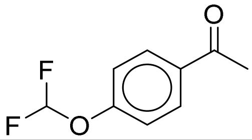
**88.**	O=C(Nc1nn(C)nn1)c2ccc(F)cc2	C9H8FN5O	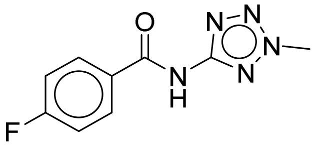
**89.**	O=C(c1ccc(F)cc1)N(C)CC(=O)O	C10H10FNO3	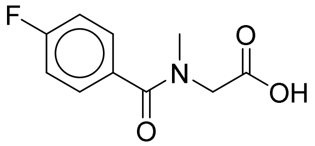
**90.**	CN2CCC(Oc1cc(F)ccc1)C2=O	C11H12FNO2	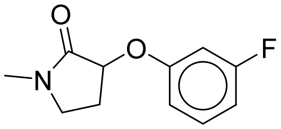
**91.**	Fc2ccc(CNC[C@H]1CCCO1)cc2	C12H16FNO	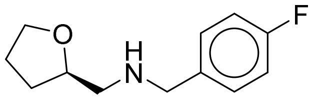
**92.**	Cl.Fc1ccc(cc1)C(C)NC	C9H13ClFN	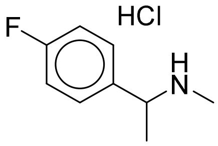
**93.**	Fc1cc(CN(C)S(N)(=O)=O)ccc1	C8H11FN2O2S	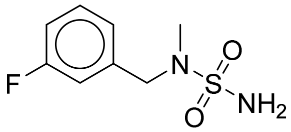
**94.**	O=C(c1c(F)cccc1F)N2CCCC2	C11H11F2NO	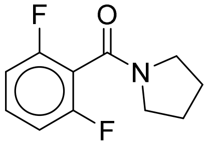
**95.**	O=C(NCc1ccc(F)cc1)c2cn(CC)nc2	C13H14FN3O	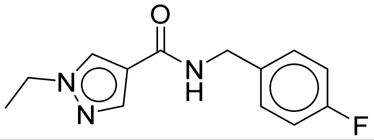

**96.**	Cl.Cl.CC(c1ccccc1F)N2CCNCC2	C12H19Cl2FN2	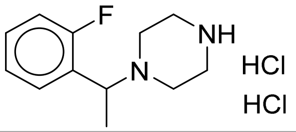
**97.**	Oc2cccc(NC(=O)Nc1ccc(F)cc1)c2	C13H11FN2O2	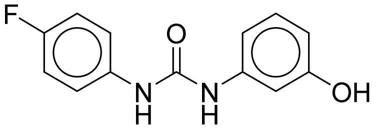
**98.**	O=C(C)Nc1cc(ccc1F)C(=O)O	C9H8FNO3	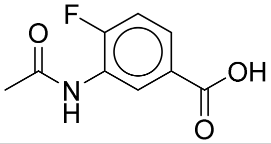
**99.**	COc1cc(ccc1)C(=O)Nc2ccccc2F	C14H12FNO2	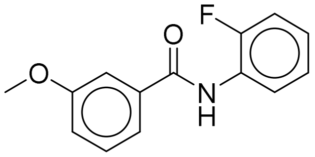
**100.**	CN1CCN(CC1=O)c2ncccc2F	C10H12FN3O	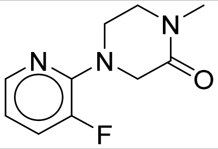
**101.**	CC(Oc1ccccc1F)C(=O)O	C9H9FO3	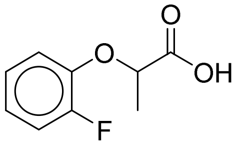

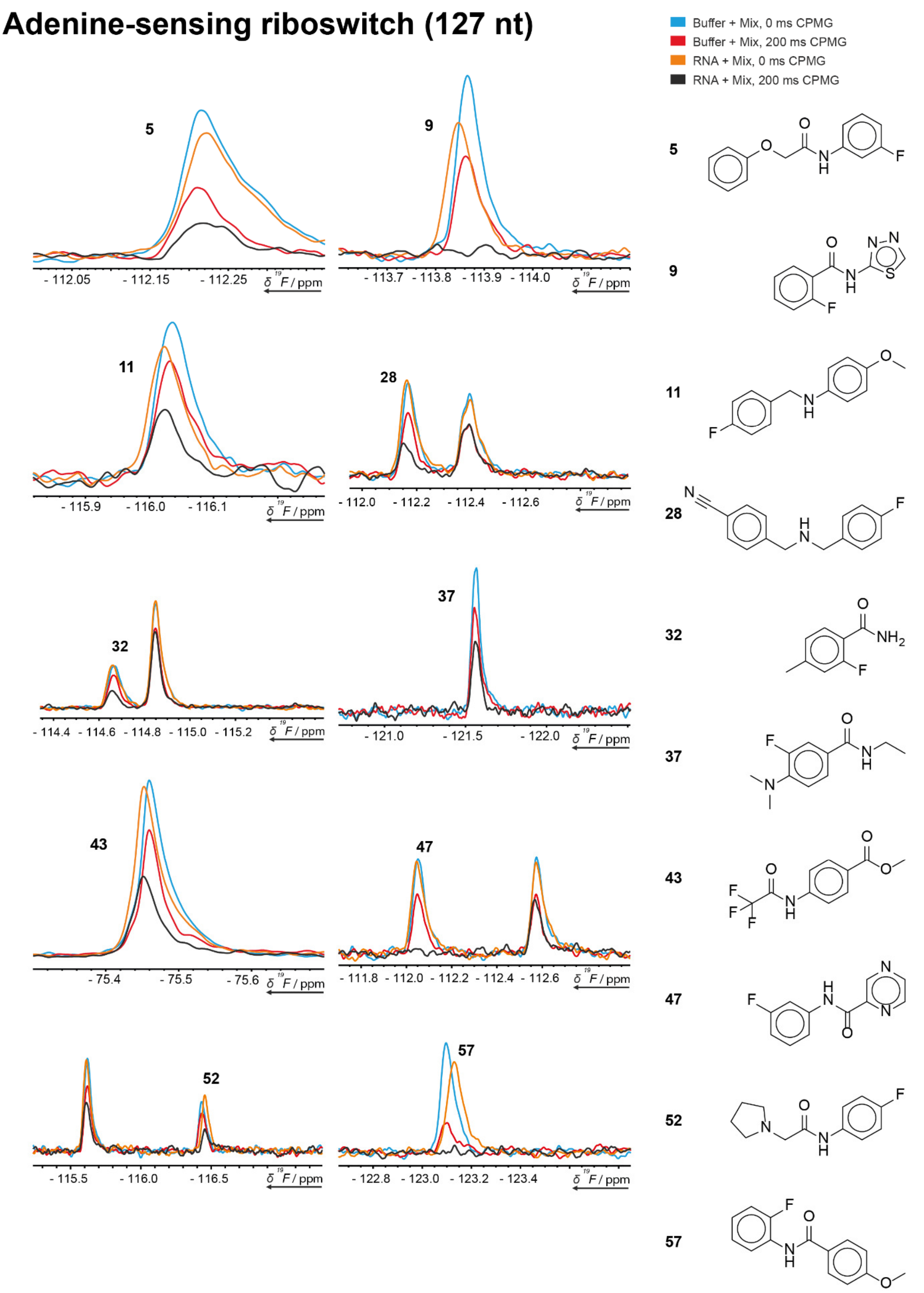

Supplementary Figure 5: Spectral excerpts displaying the intensity modulation obtained in the 200 ms CPMG experiment against 0 ms CPMG identifying the respective fragments as target hits for the adenine-sensing riboswitch (127 nt) from Vibrio vulnificus.

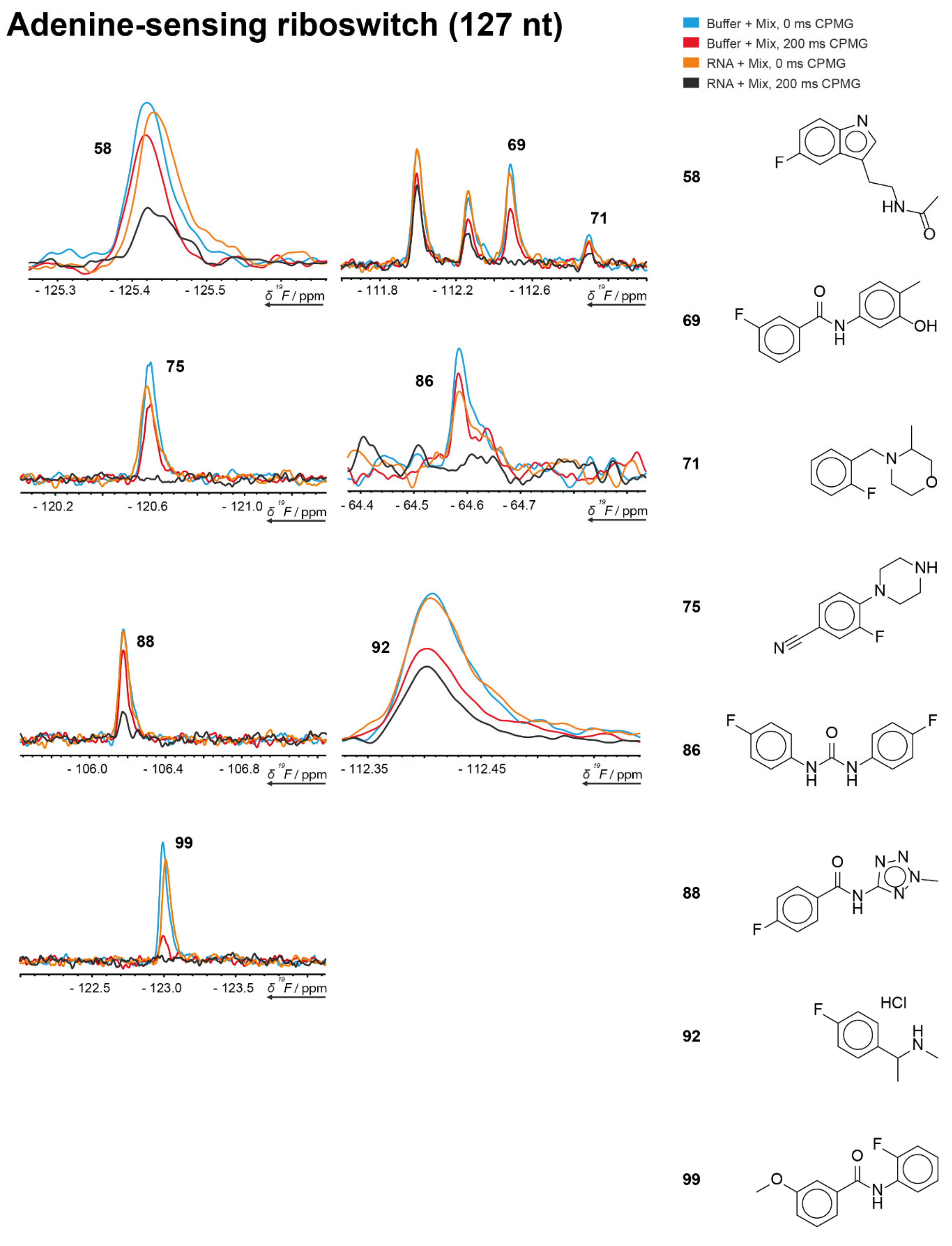

Supplementary Figure 6: Spectral excerpts displaying the intensity modulation obtained in the 200 ms CPMG experiment against 0 ms CPMG identifying the respective fragments as target hits for the adenine-sensing riboswitch (127 nt) from Vibrio vulnificus.

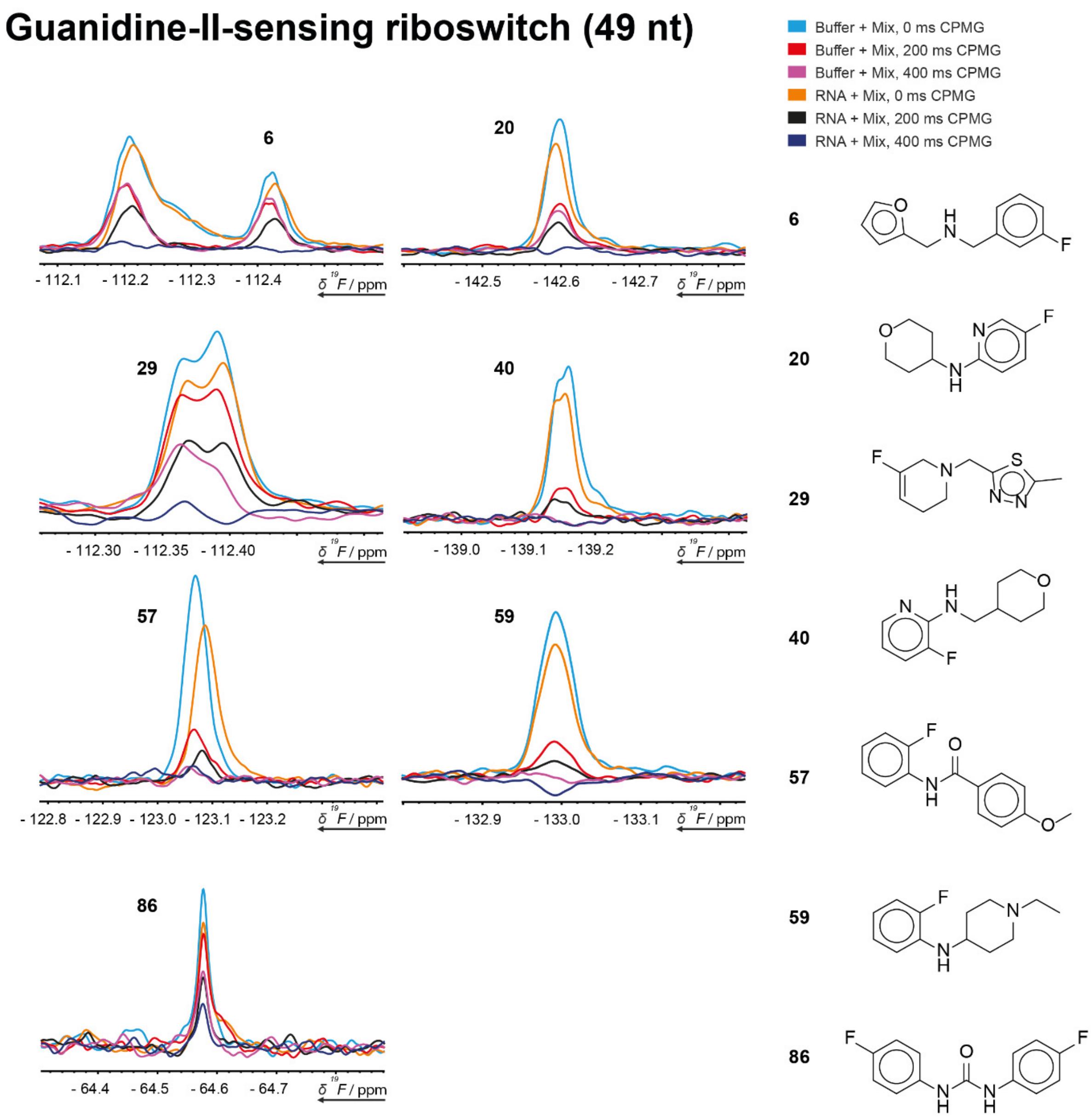

Supplementary Figure 7: Spectral excerpts displaying the intensity modulation obtained in the 200 ms CPMG experiment against 0 ms CPMG identifying the respective fragments as target hits for the Guanidine-II riboswitch (49 nt) from E. coli. For further hit validation, the CPMG experiment at 400 ms is shown.

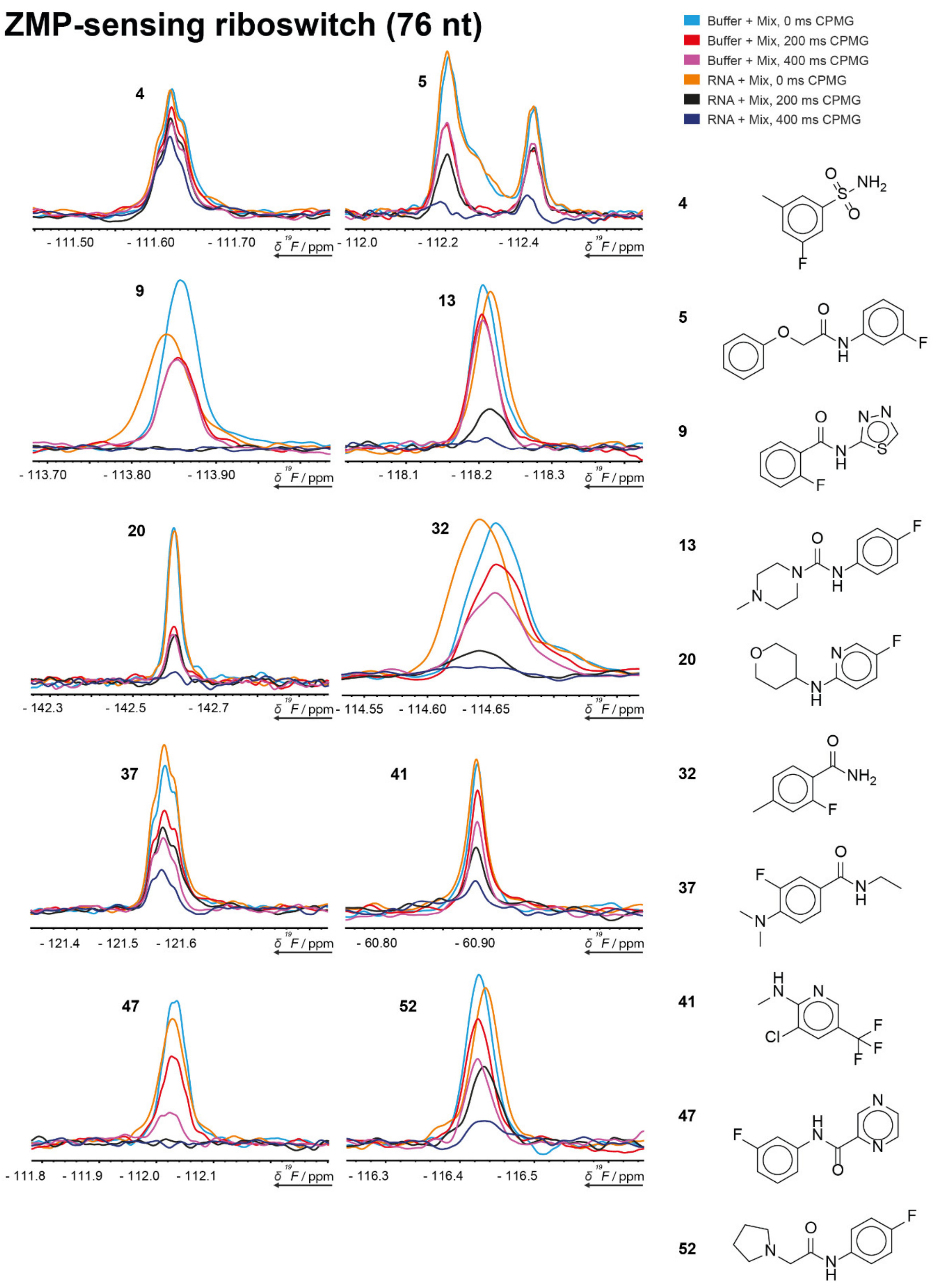

Supplementary Figure 8: Spectral excerpts displaying the intensity modulation obtained in the 200 ms CPMG experiment against 0 ms CPMG identifying the respective fragments as target hits for the ZMP-sensing riboswitch (76 nt) from Thermosinus carboxydivorans. For further hit validation, the CPMG experiment at 400 ms is shown.

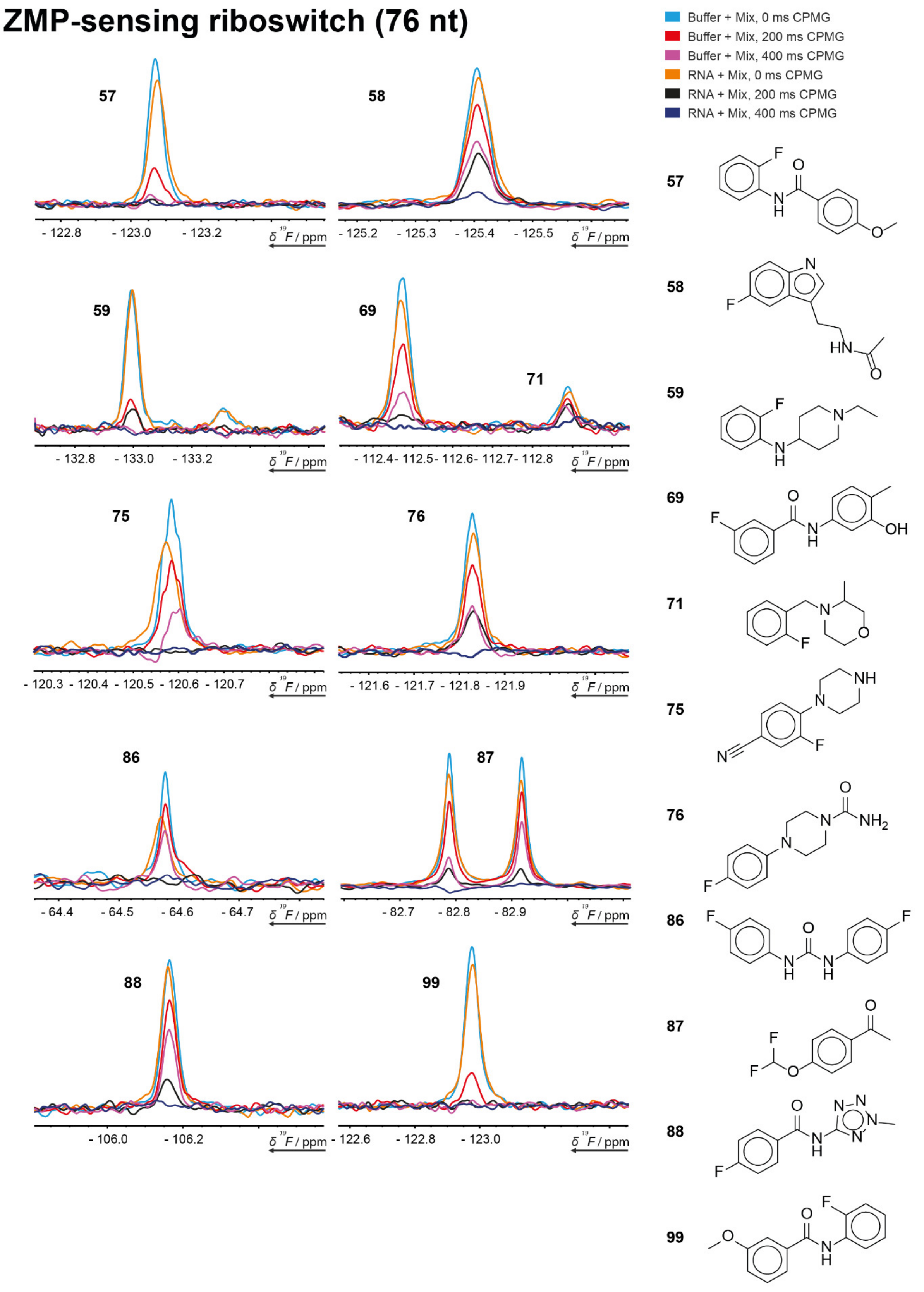

Supplementary Figure 9: Spectral excerpts displaying the intensity modulation obtained in the 200 ms CPMG experiment against 0 ms CPMG identifying the respective fragments as target hits for the ZMP-sensing riboswitch (76 nt) from Thermosinus carboxydivorans. For further hit validation, the CPMG experiment at 400 ms is shown.

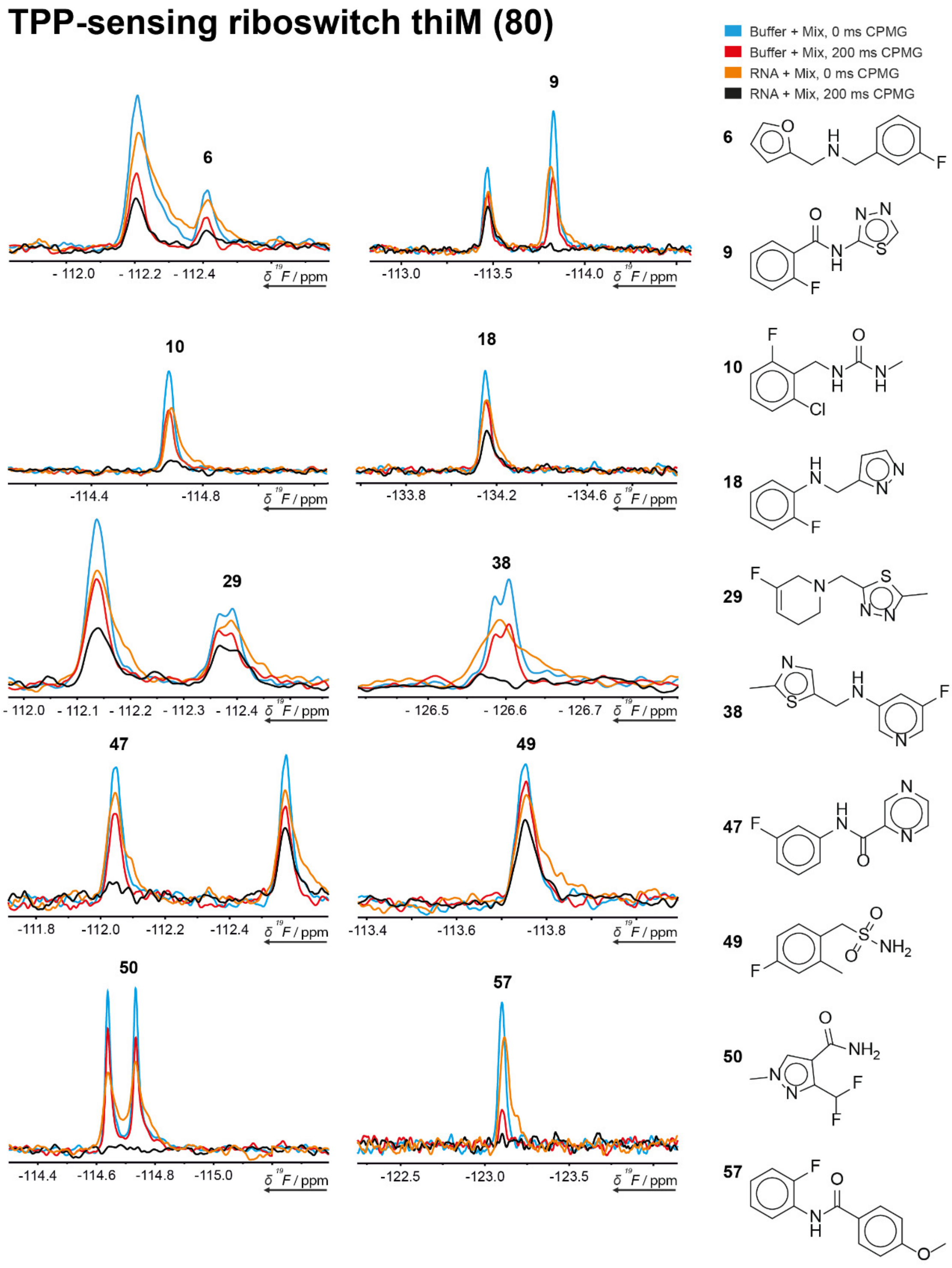

Supplementary Figure 10: Spectral excerpts displaying the intensity modulation obtained in the 200 ms CPMG experiment against 0 ms CPMG identifying the respective fragments as target hits for the thiM TPP-sensing riboswitch (80 nt).

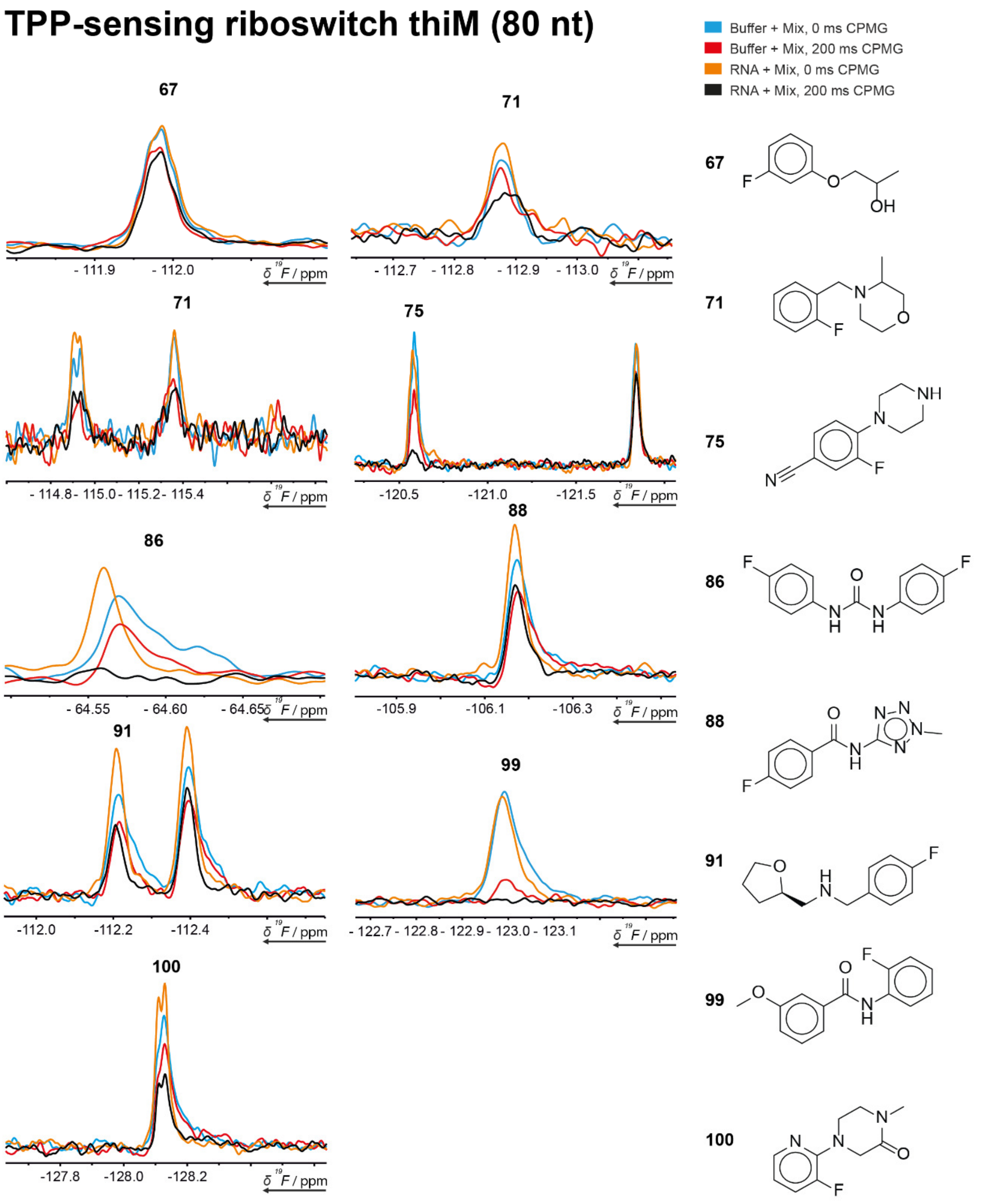

Supplementary Figure 11: Spectral excerpts displaying the intensity modulation obtained in the 200 ms CPMG experiment against 0 ms CPMG identifying the respective fragments as target hits for the thiM TPP-sensing riboswitch (80 nt).

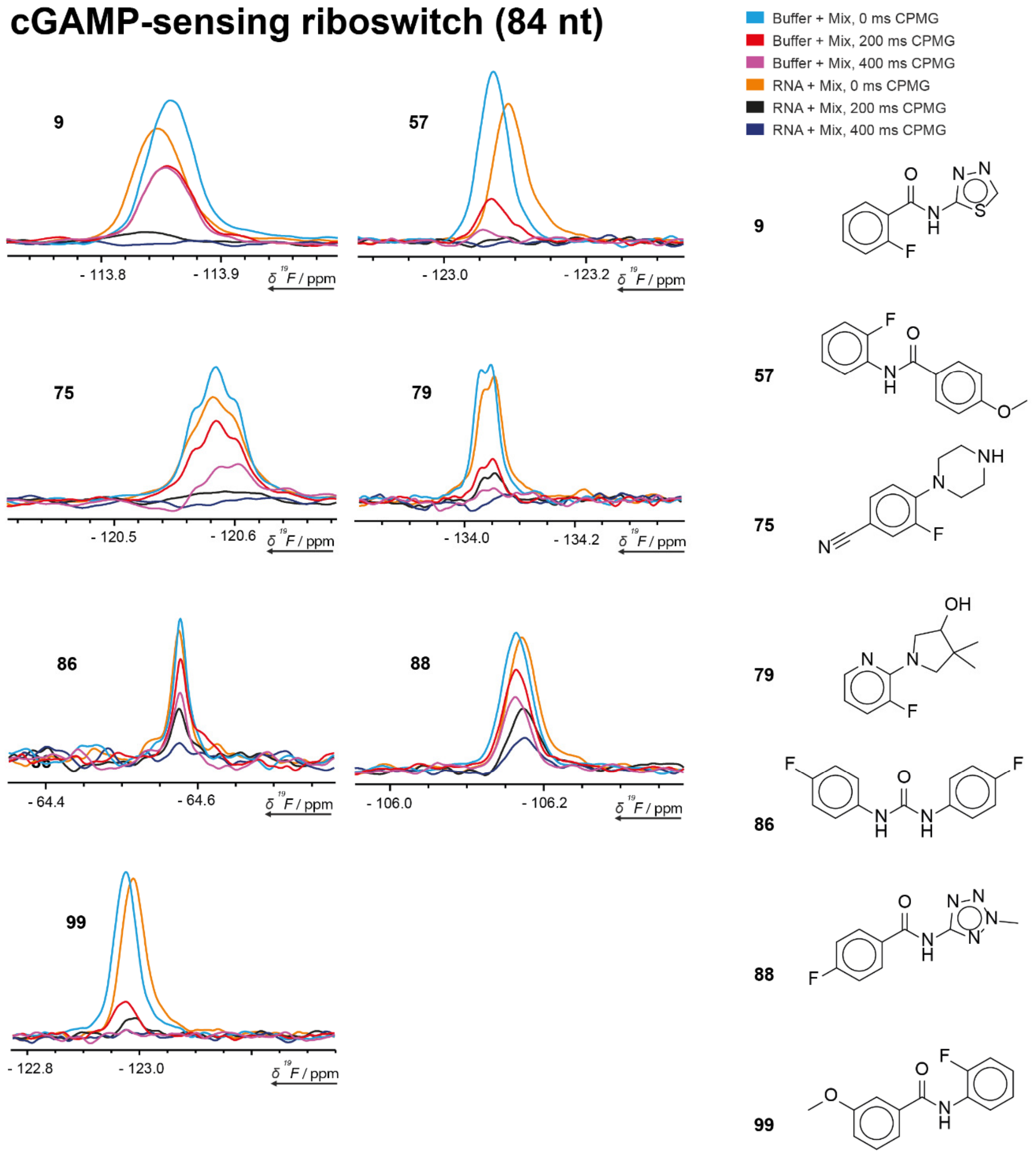

Supplementary Figure 12: Spectral excerpts displaying the intensity modulation obtained in the 200 ms CPMG experiment against 0 ms CPMG identifying the respective fragments as target hits for the pilM 3',3'-cGAMP-sensing riboswitch (84 nt). For further hit validation, the CPMG experiment at 400 ms is shown.

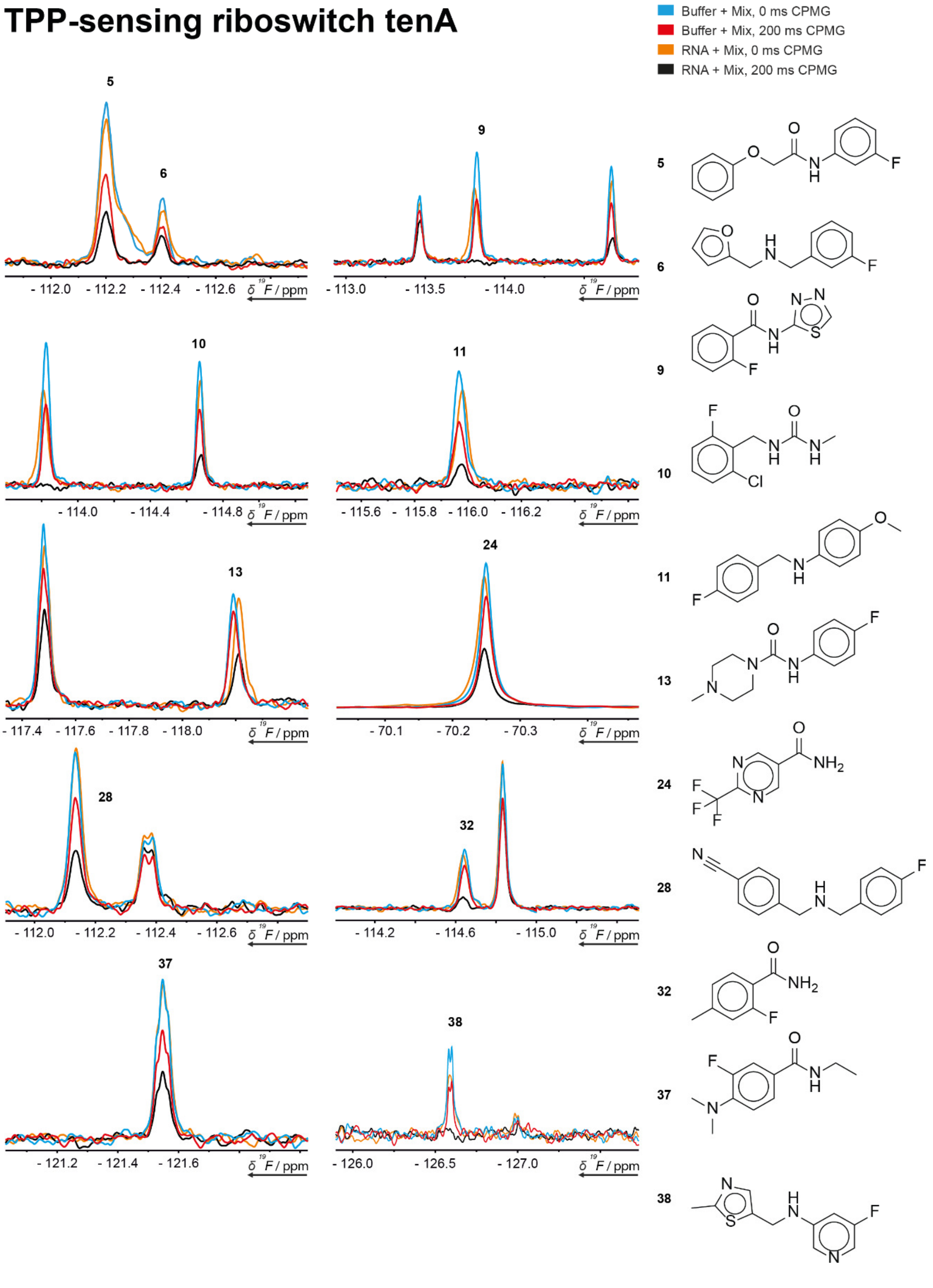

Supplementary Figure 13: Spectral excerpts displaying the intensity modulation obtained in the 200 ms CPMG experiment against 0 ms CPMG identifying the respective fragments as target hits for the tenA TTP- sensing riboswitch (94 nt).

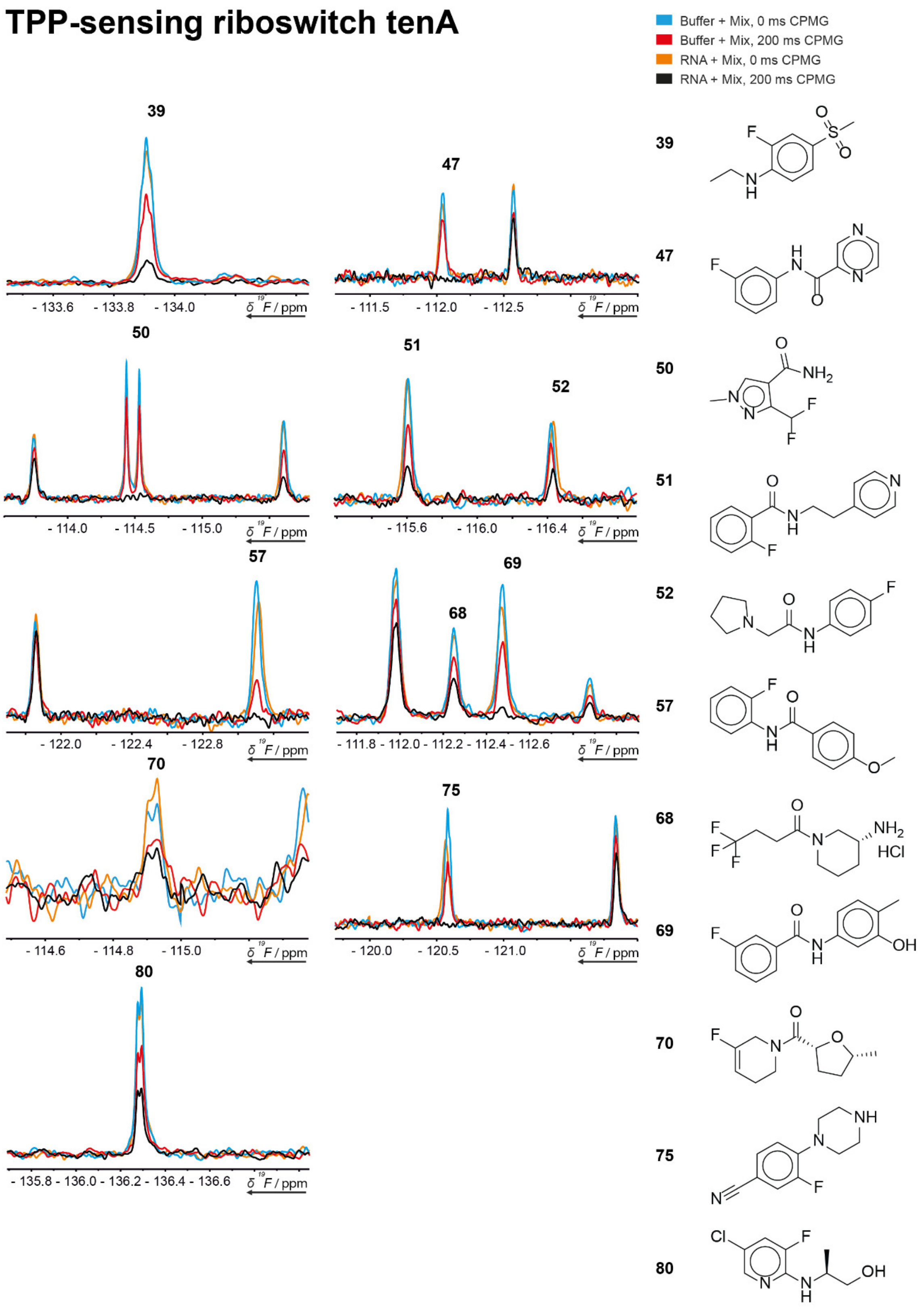

Supplementary Figure 14: Spectral excerpts displaying the intensity modulation obtained in the 200 ms CPMG experiment against 0 ms CPMG identifying the respective fragments as target hits for the tenA TTP- sensing riboswitch (94 nt).

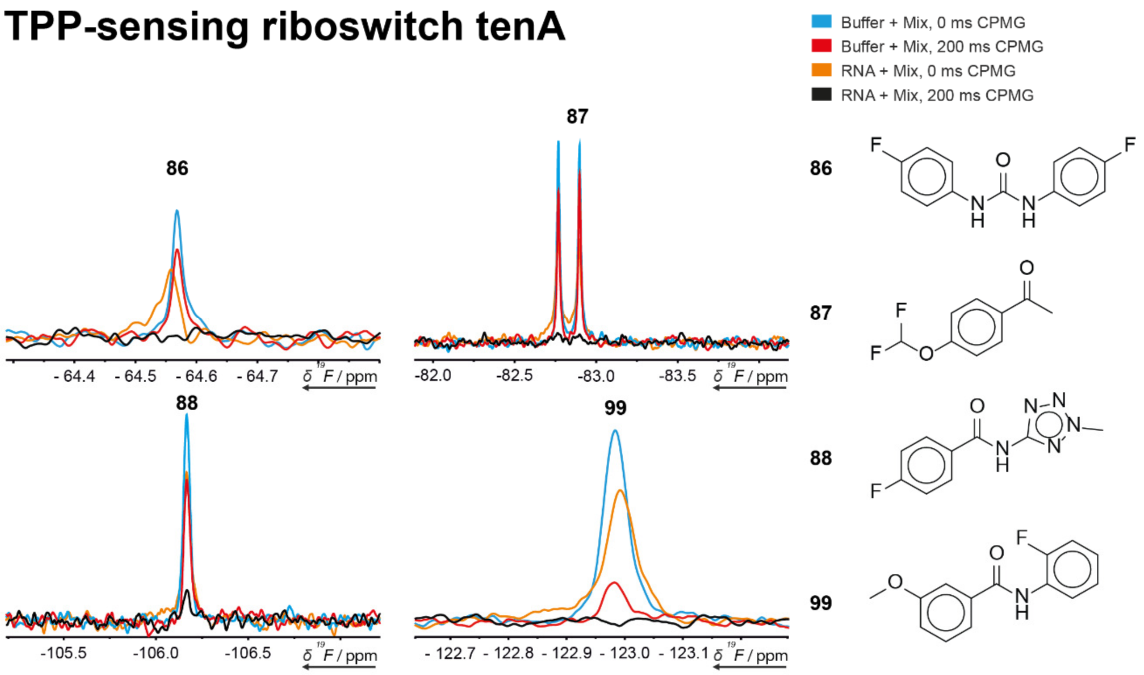

Supplementary Figure 15: Spectral excerpts displaying the intensity modulation obtained in the 200 ms CPMG experiment against 0 ms CPMG identifying the respective fragments as target hits for the tenA TTP- sensing riboswitch (94 nt).

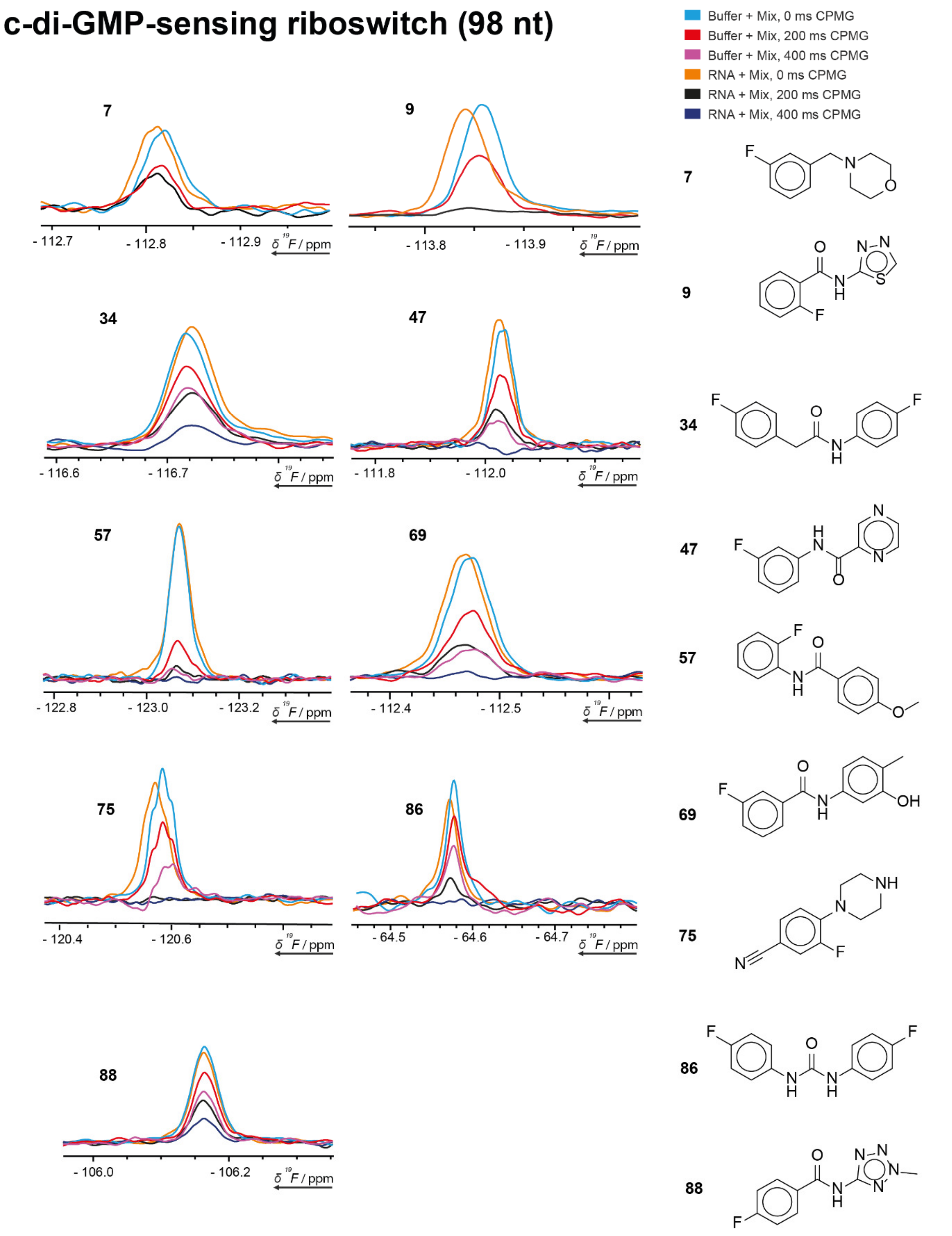

Supplementary Figure 16: Spectral excerpts displaying the intensity modulation obtained in the 200 ms CPMG experiment against 0 ms CPMG identifying the respective fragments as target hits for the cyclic di-GMP-1 riboswitch (98 nt). For further hit validation, the CPMG experiment at 400 ms is shown.

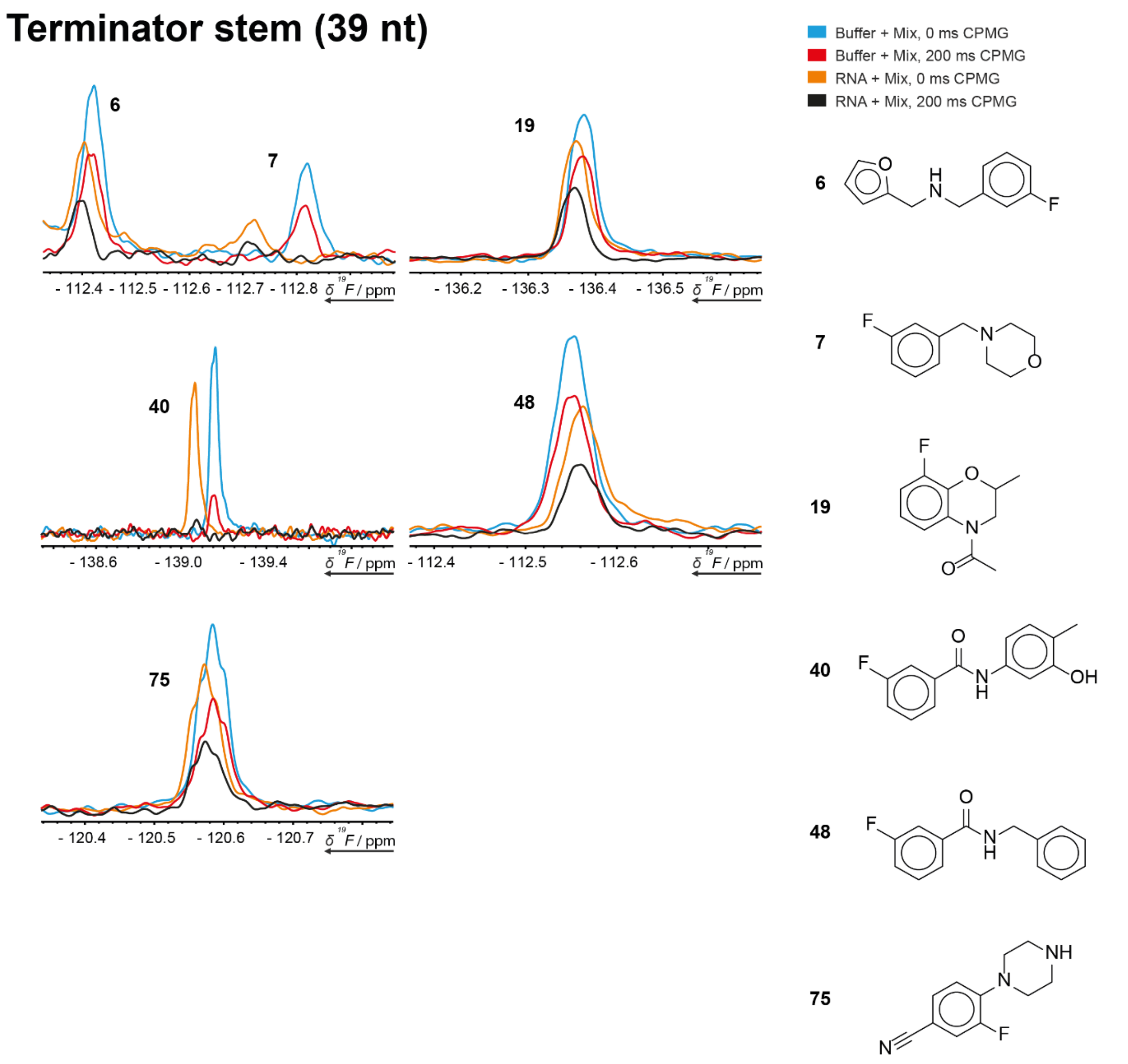

Supplementary Figure 17: Spectral excerpts displaying the intensity modulation obtained in the 200 ms CPMG experiment against 0 ms CPMG identifying the respective fragments as target hits for the 2'-deoxyguanosine-sensing-riboswitch terminator (39 nt).

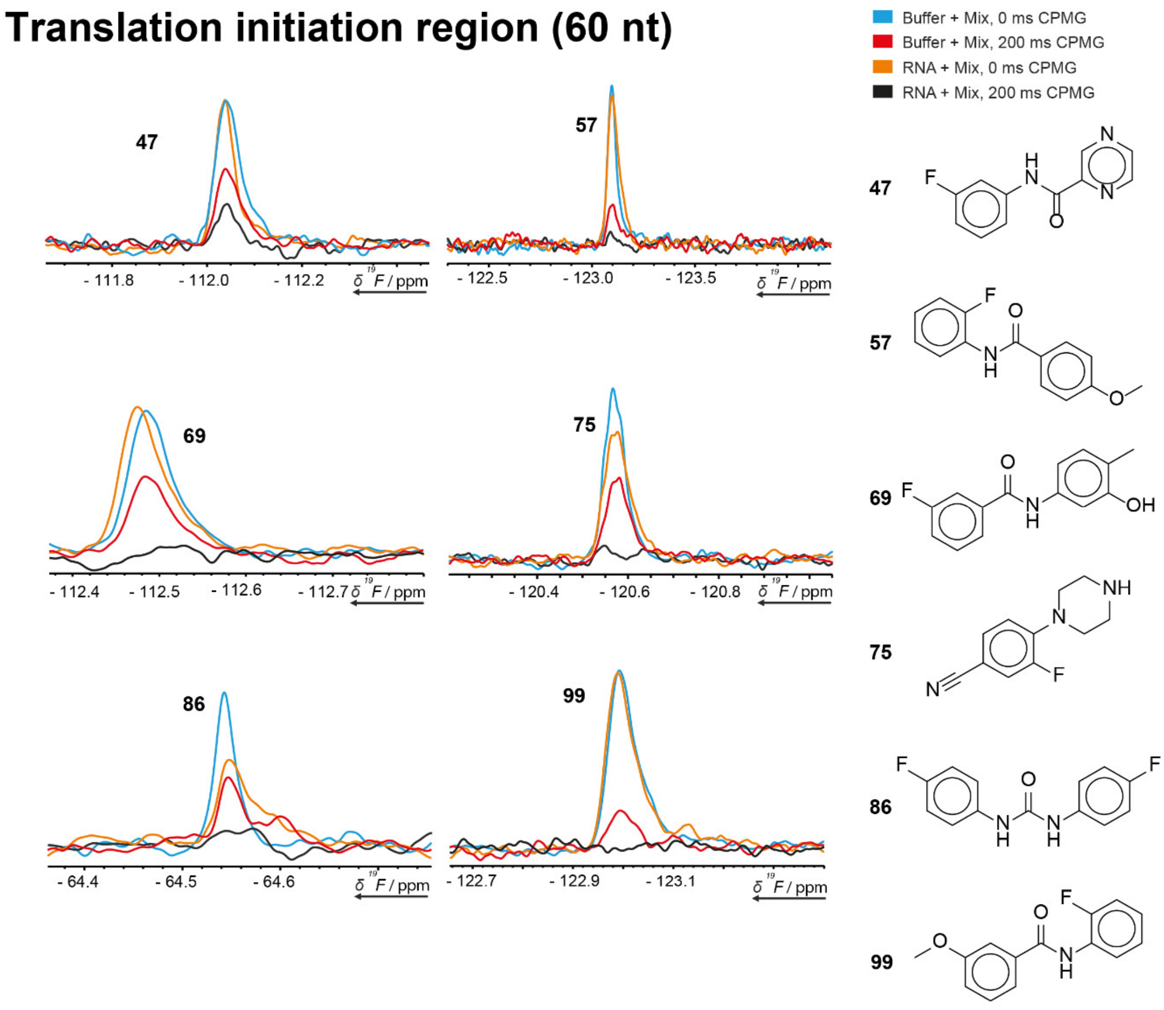

Supplementary Figure 18: Spectral excerpts displaying the intensity modulation obtained in the 200 ms CPMG experiment against 0 ms CPMG identifying the respective fragments as target hits for the Adenine-sensing riboswitch expression platform (60 nt).

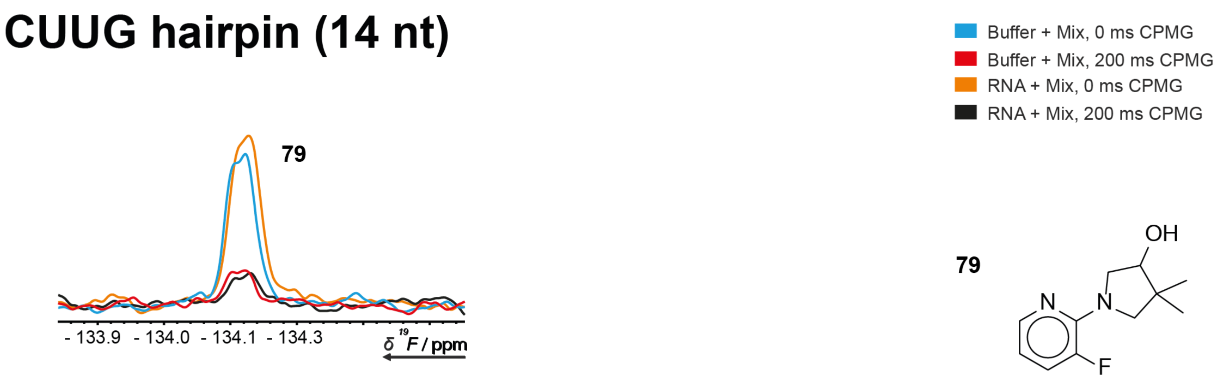

Supplementary Figure 19: Spectral excerpts displaying the intensity modulation obtained in the 200 ms CPMG experiment against 0 ms CPMG identifying the respective fragments as target hits for the RNA with CUUG tetraloop (14 nt).

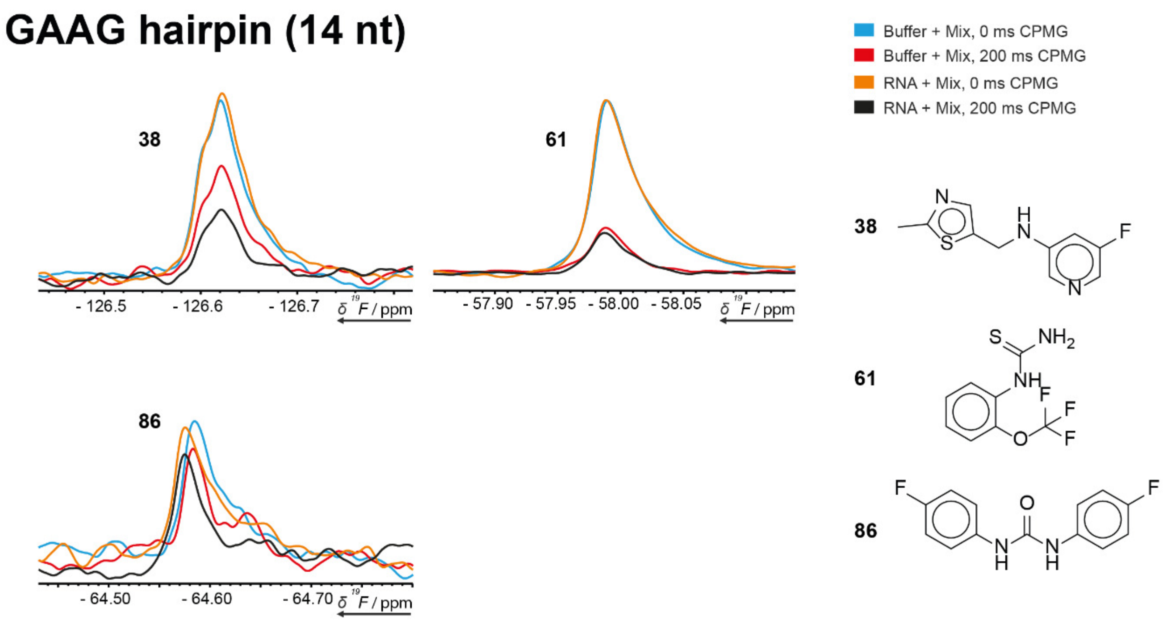

Supplementary Figure 20: Spectral excerpts displaying the intensity modulation obtained in the 200 ms CPMG experiment against 0 ms CPMG identifying the respective fragments as target hits for the RNA with GAAG tetraloop (14 nt).

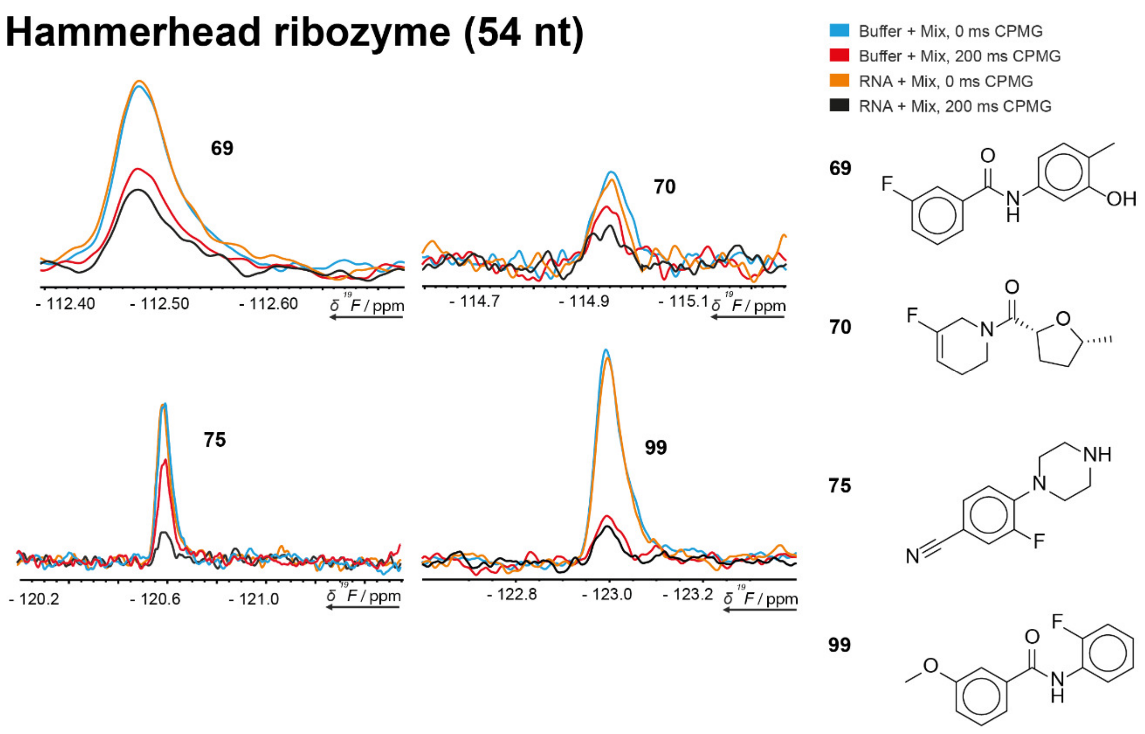

Supplementary Figure 21: Spectral excerpts displaying the intensity modulation obtained in the 200 ms CPMG experiment against 0 ms CPMG identifying the respective fragments as target hits for the Hammerhead ribozyme (54 nt).

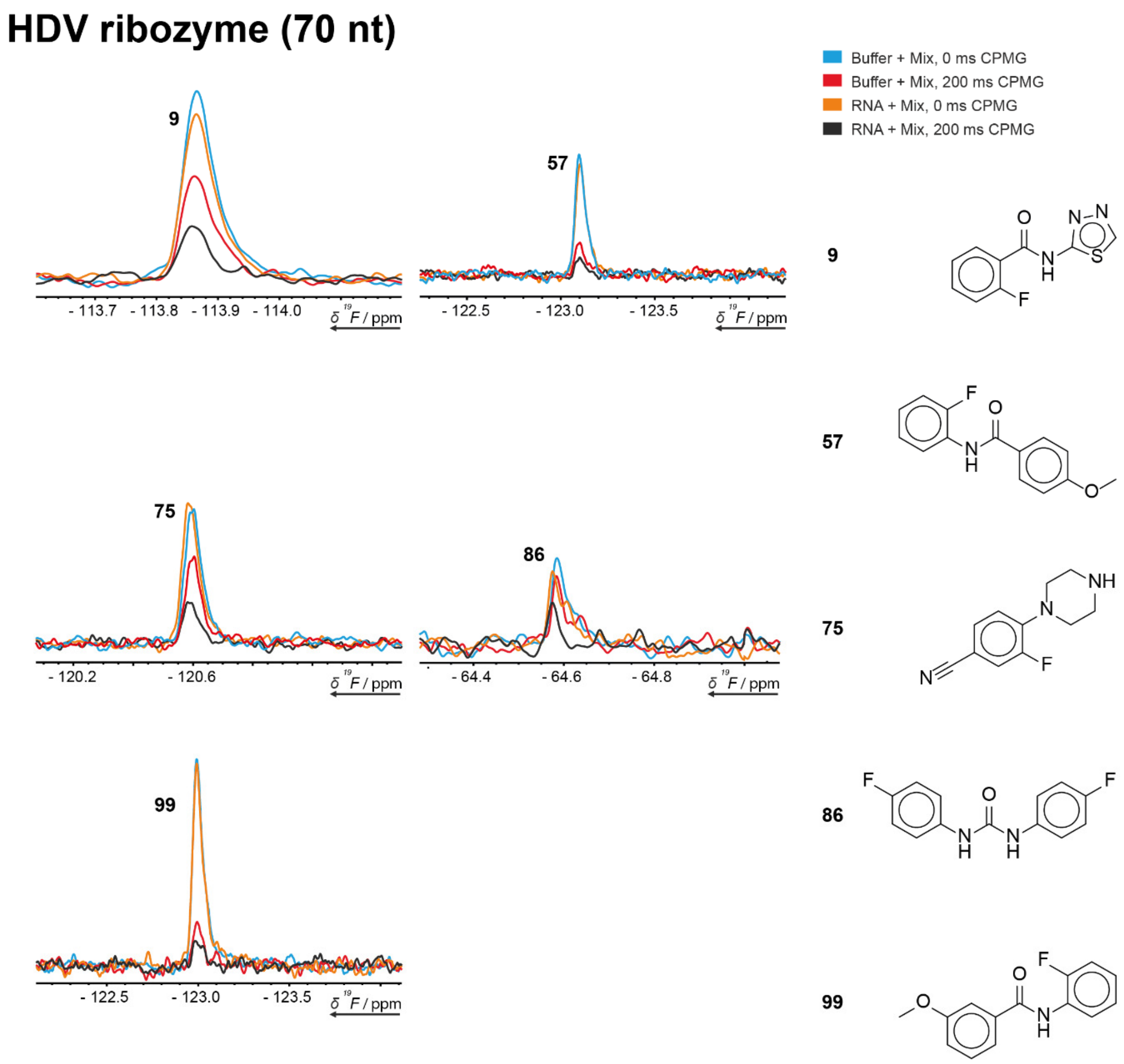

Supplementary Figure 22: Spectral excerpts displaying the intensity modulation obtained in the 200 ms CPMG experiment against 0 ms CPMG identifying the respective fragments as target hits for the Hepatitis delta virus ribozyme (70 nt).

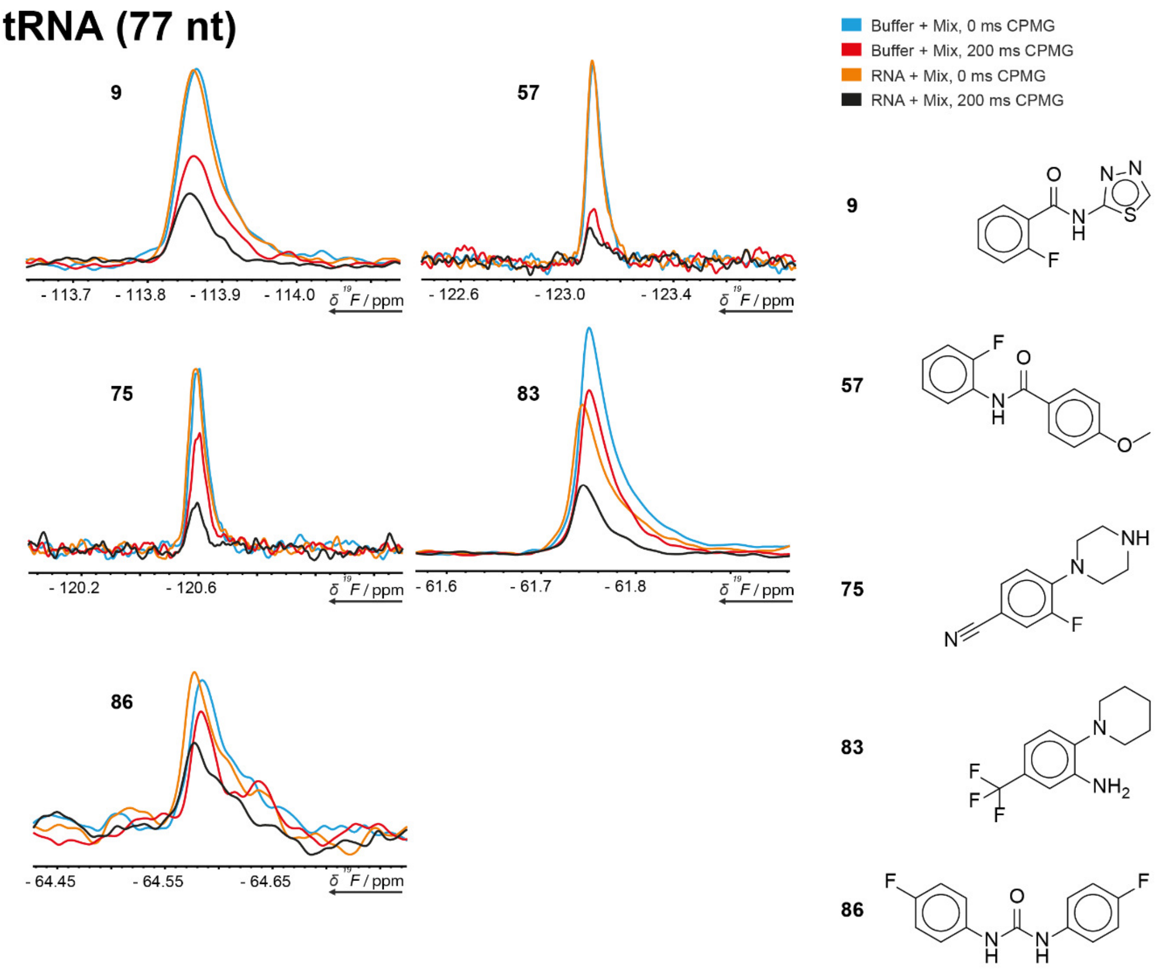

Supplementary Figure 23: Spectral excerpts displaying the intensity modulation obtained in the 200 ms CPMG experiment against 0 ms CPMG identifying the respective fragments as target hits for the initiator tRNA^{fMet} (77 nt).

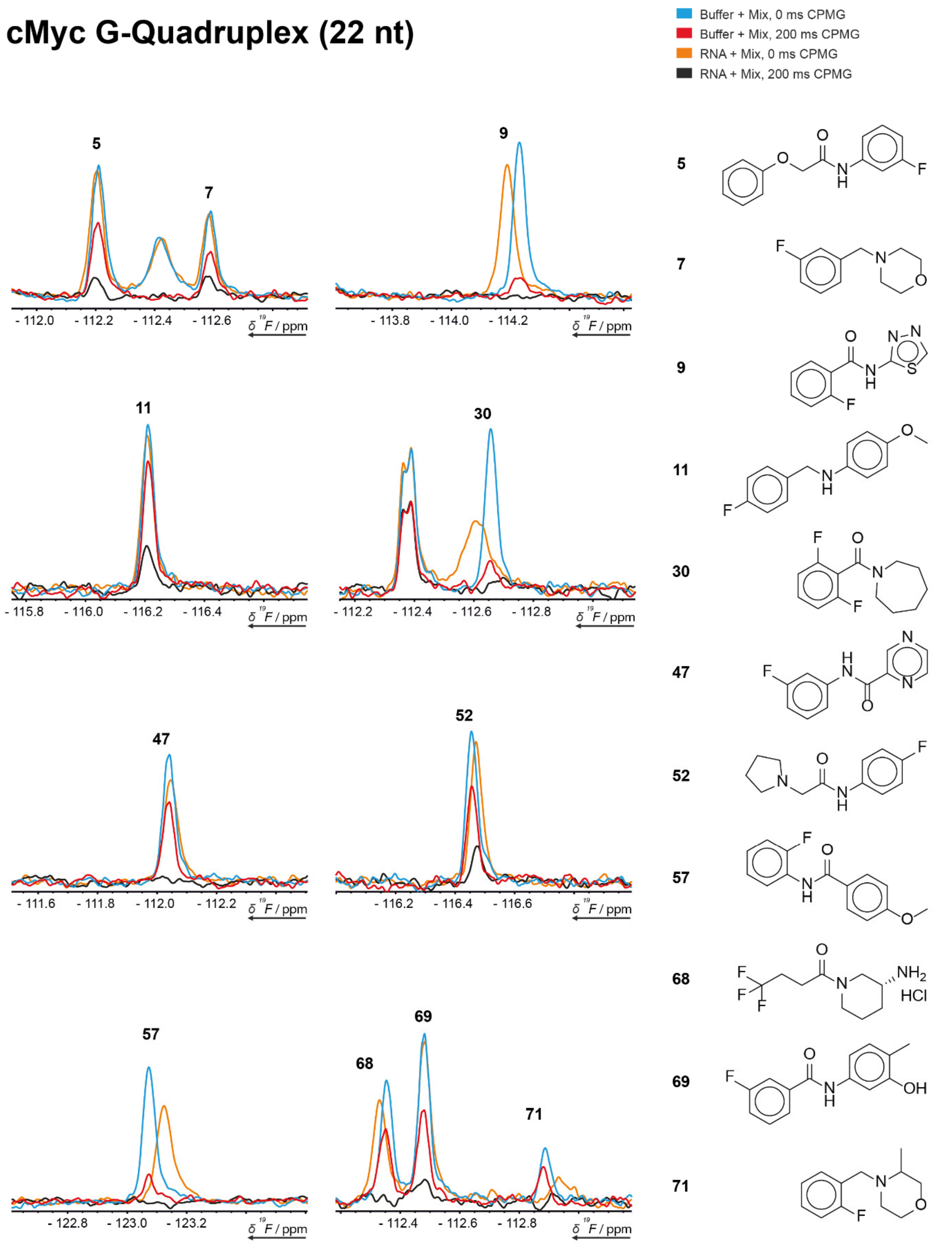

Supplementary Figure 24: Spectral excerpts displaying the intensity modulation obtained in the 200 ms CPMG experiment against 0 ms CPMG identifying the respective fragments as target hits for the cMyc G-Quadruplex (22 nt).

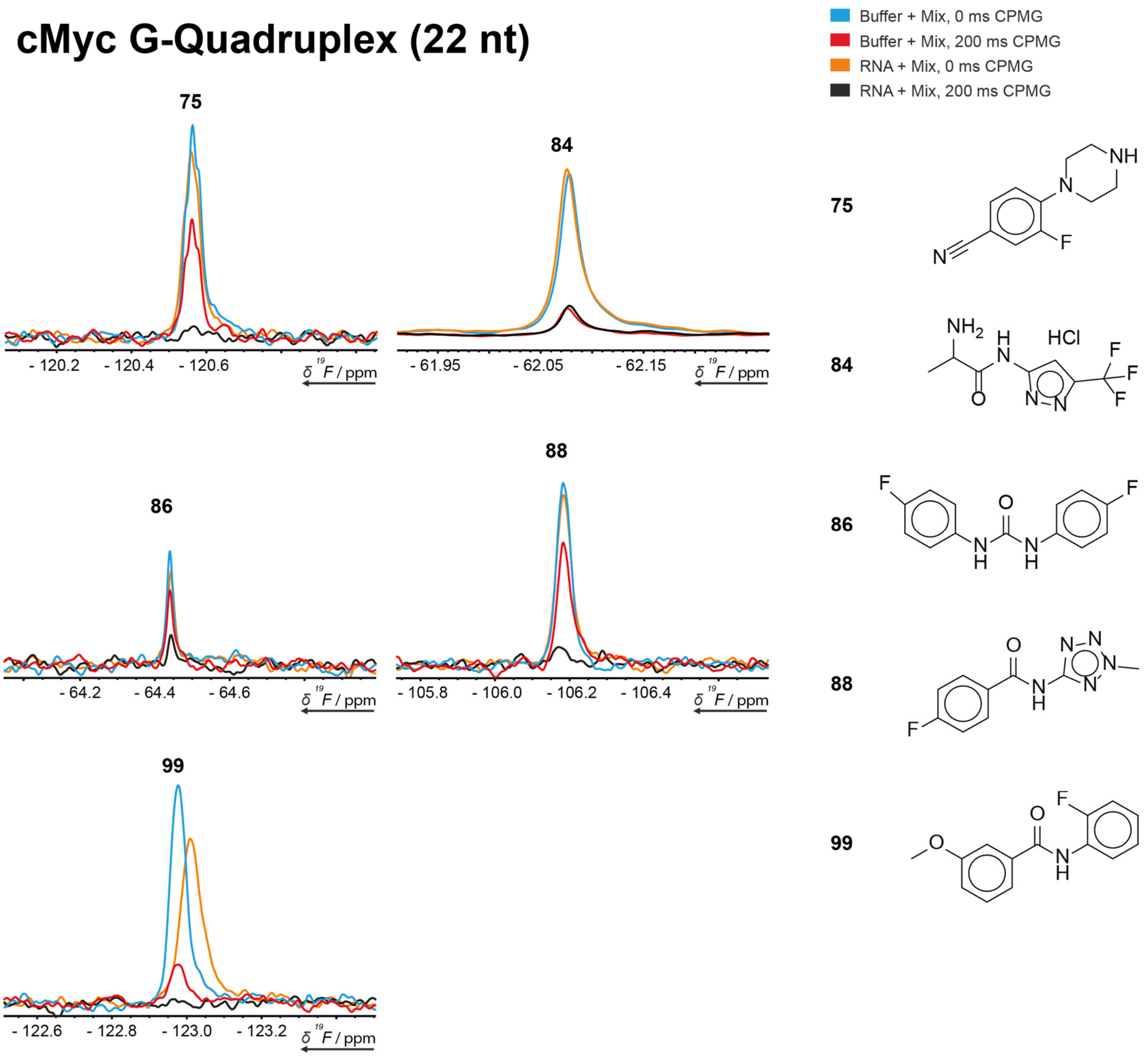

Supplementary Figure 25: Spectral excerpts displaying the intensity modulation obtained in the 200 ms CPMG experiment against 0 ms CPMG identifying the respective fragments as target hits for the cMyc G-Quadruplex (22 nt).

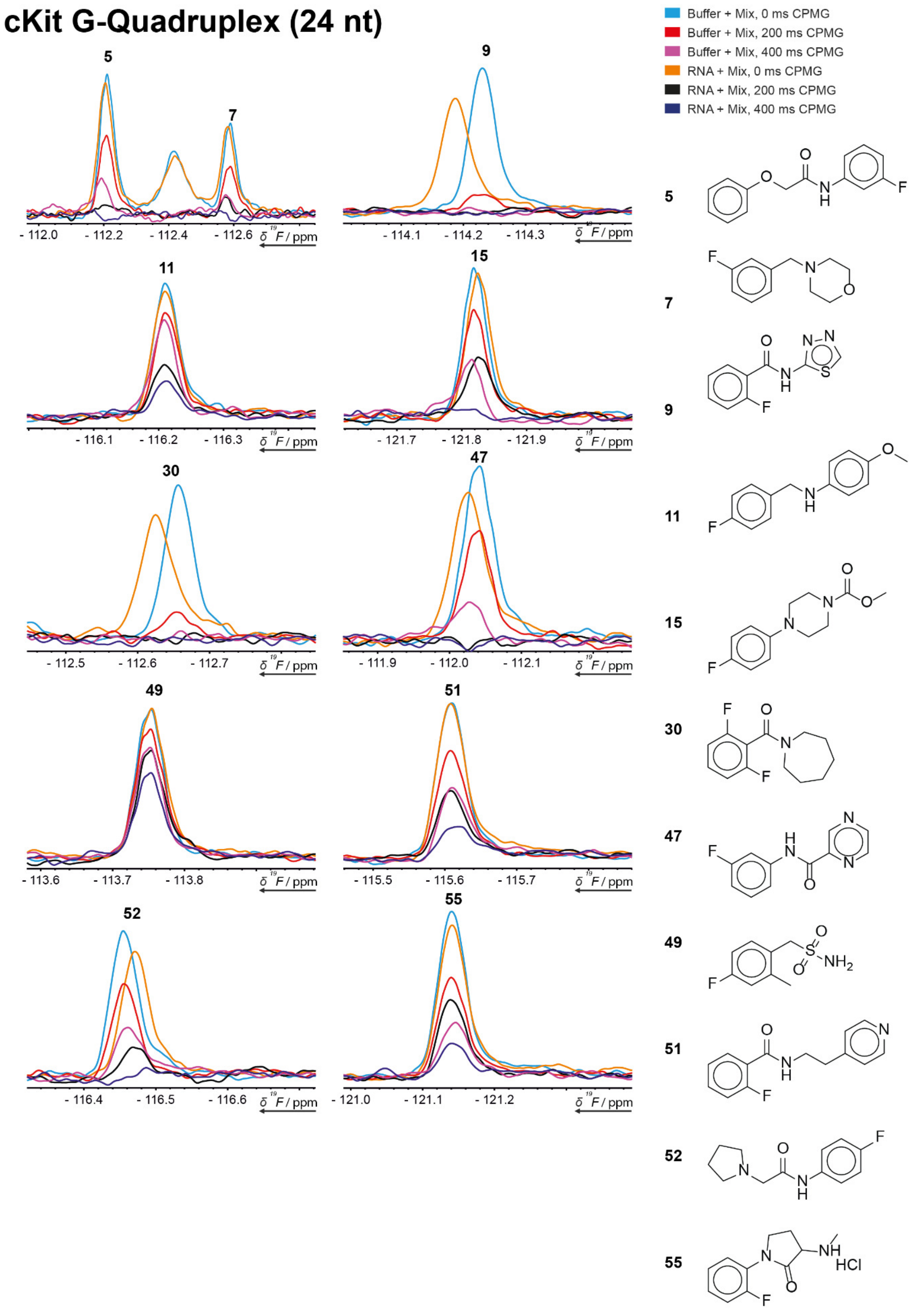

Supplementary Figure 26: Spectral excerpts displaying the intensity modulation obtained in the 200 ms CPMG experiment against 0 ms CPMG identifying the respective fragments as target hits for the cKit G-Quadruplex (24 nt). For further hit validation, the CPMG experiment at 400 ms is shown.

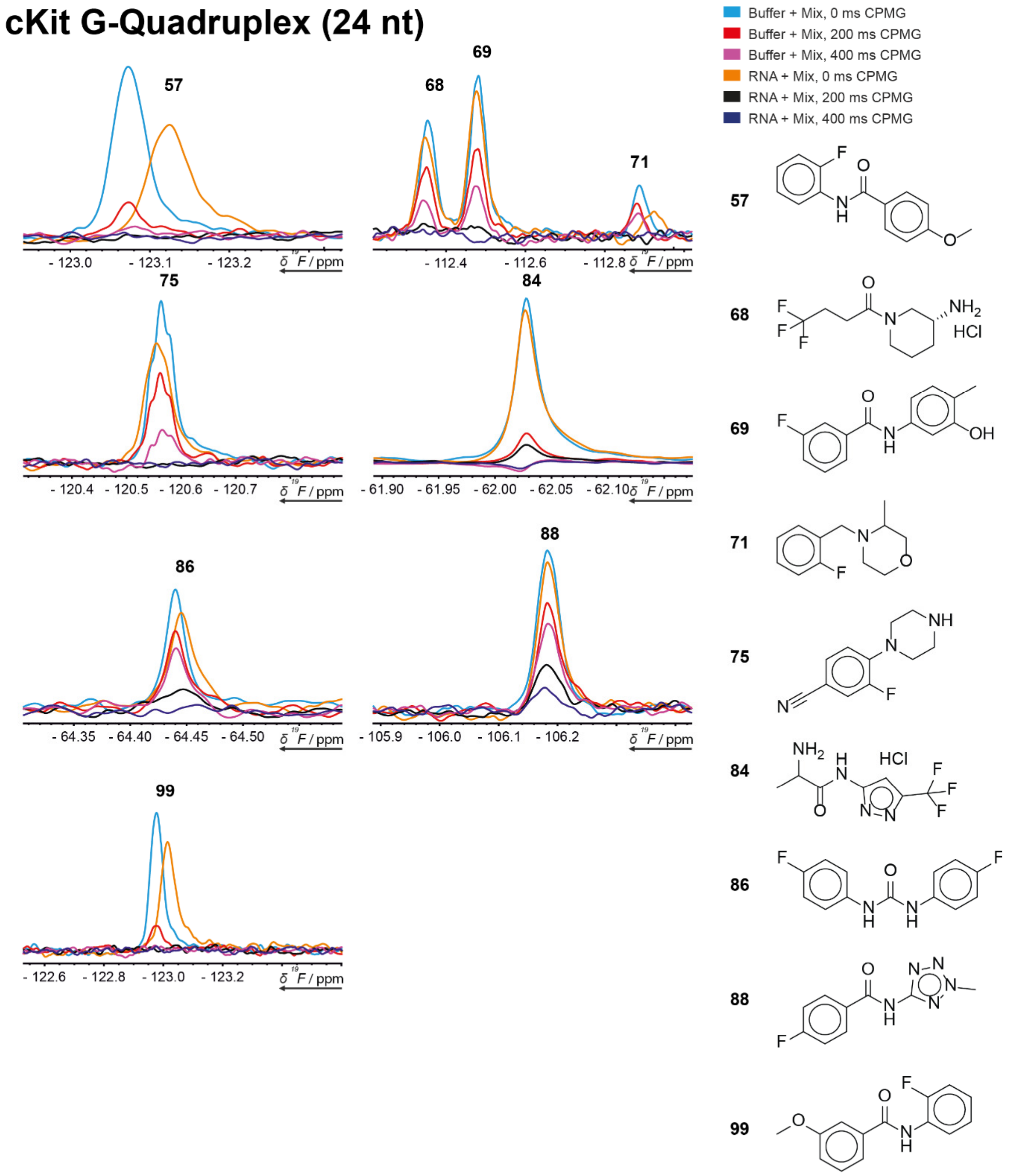

Supplementary Figure 27: Spectral excerpts displaying the intensity modulation obtained in the 200 ms CPMG experiment against 0 ms CPMG identifying the respective fragments as target hits for the cKit G-Quadruplex (24 nt). For further hit validation, the CPMG experiment at 400 ms is shown.

DNA duplex (24 nt)

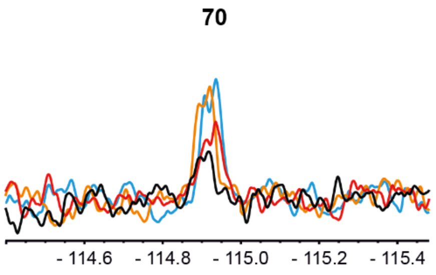

70

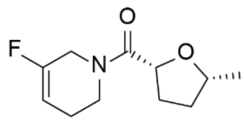

Supplementary Figure 28: Spectral excerpts displaying the intensity modulation obtained in the 200 ms CPMG experiment against 0 ms CPMG identifying the respective fragments as target hits for the DNA Duplex (24 nt).

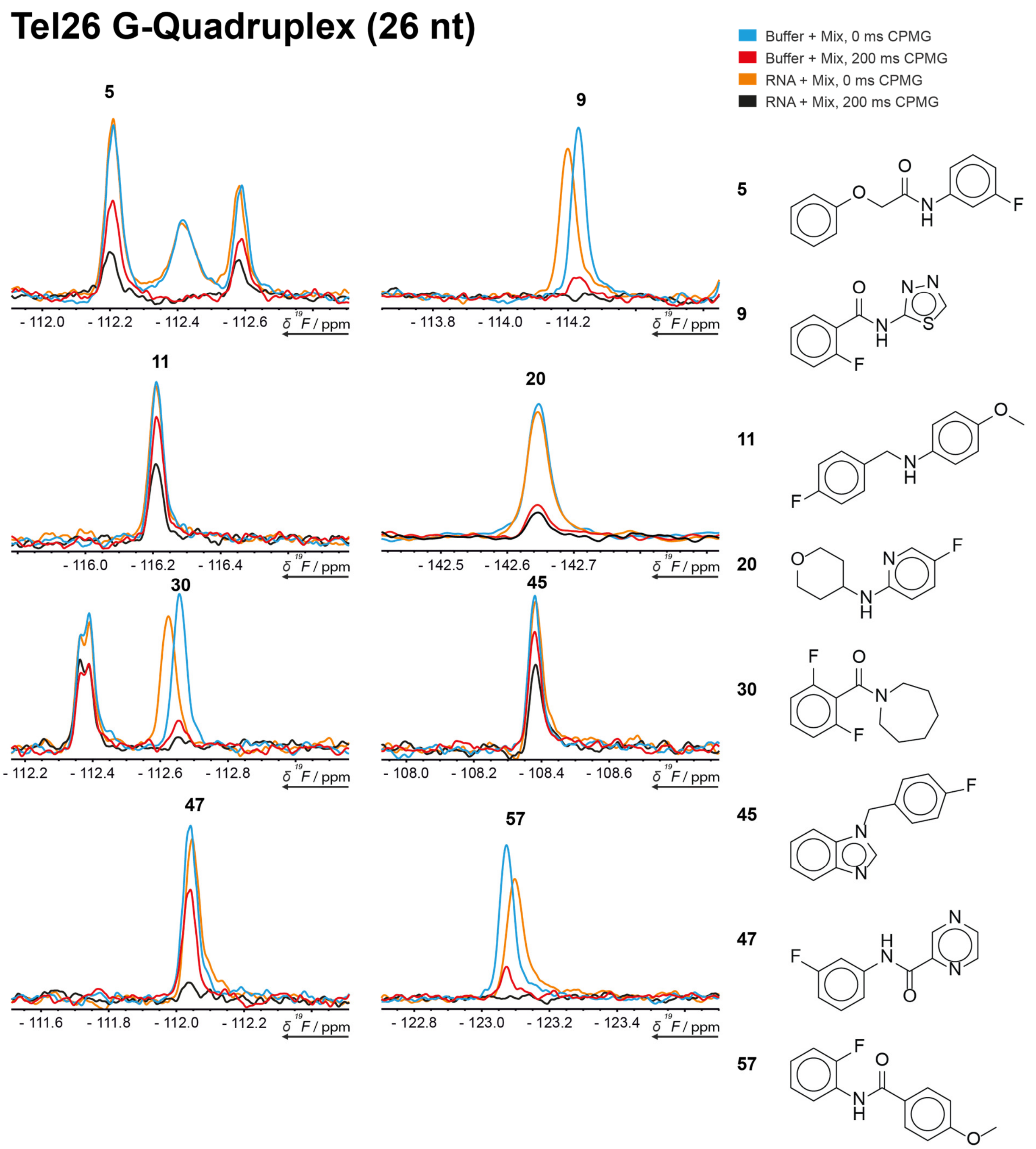

Supplementary Figure 29: Spectral excerpts displaying the intensity modulation obtained in the 200 ms CPMG experiment against 0 ms CPMG identifying the respective fragments as target hits for the Tel26 G-Quadruplex (26 nt).

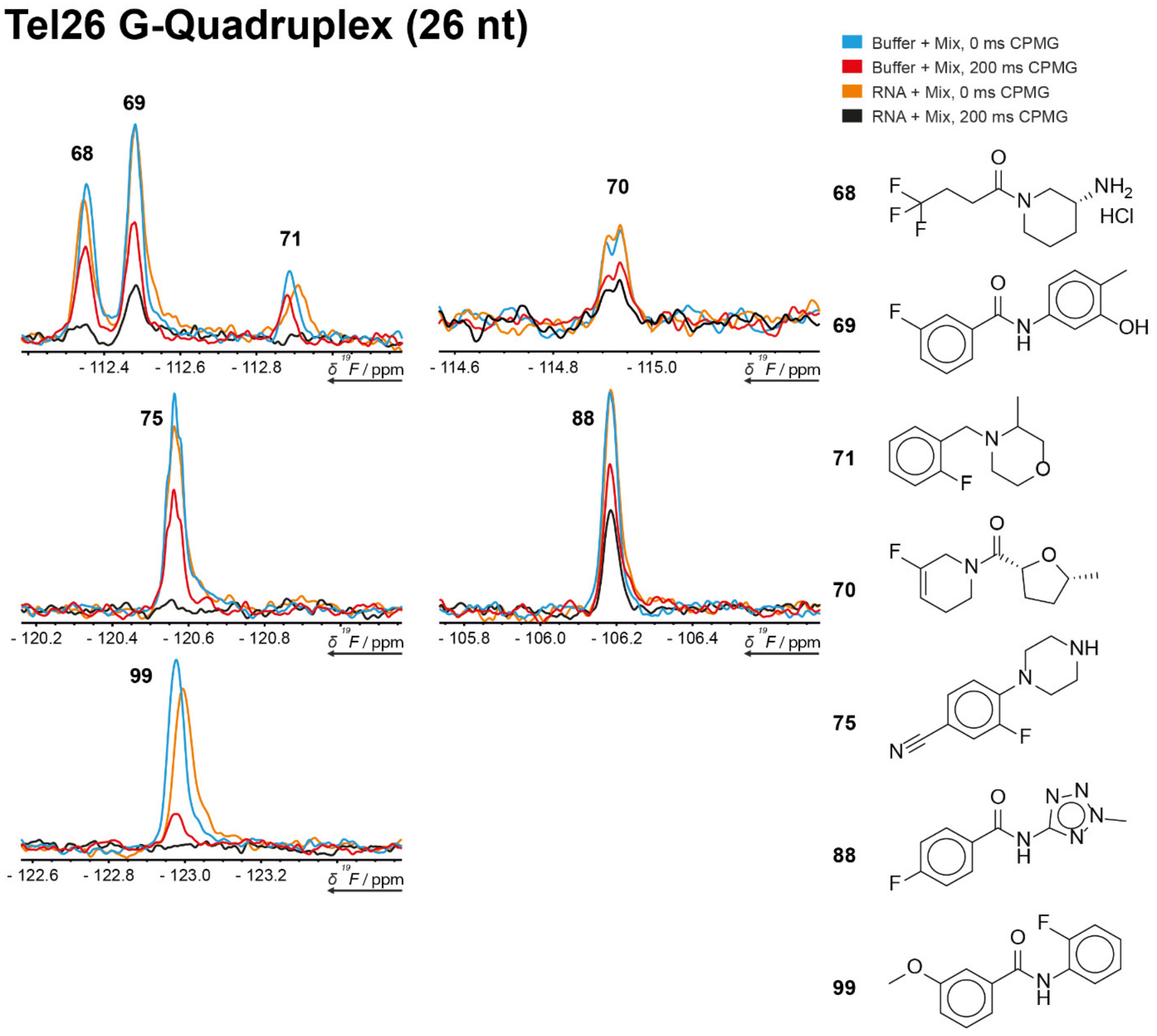

Supplementary Figure 30: Spectral excerpts displaying the intensity modulation obtained in the 200 ms CPMG experiment against 0 ms CPMG identifying the respective fragments as target hits for the Tel26 G-Quadruplex (26 nt).

wtTel26 G-Quadruplex (26 nt)

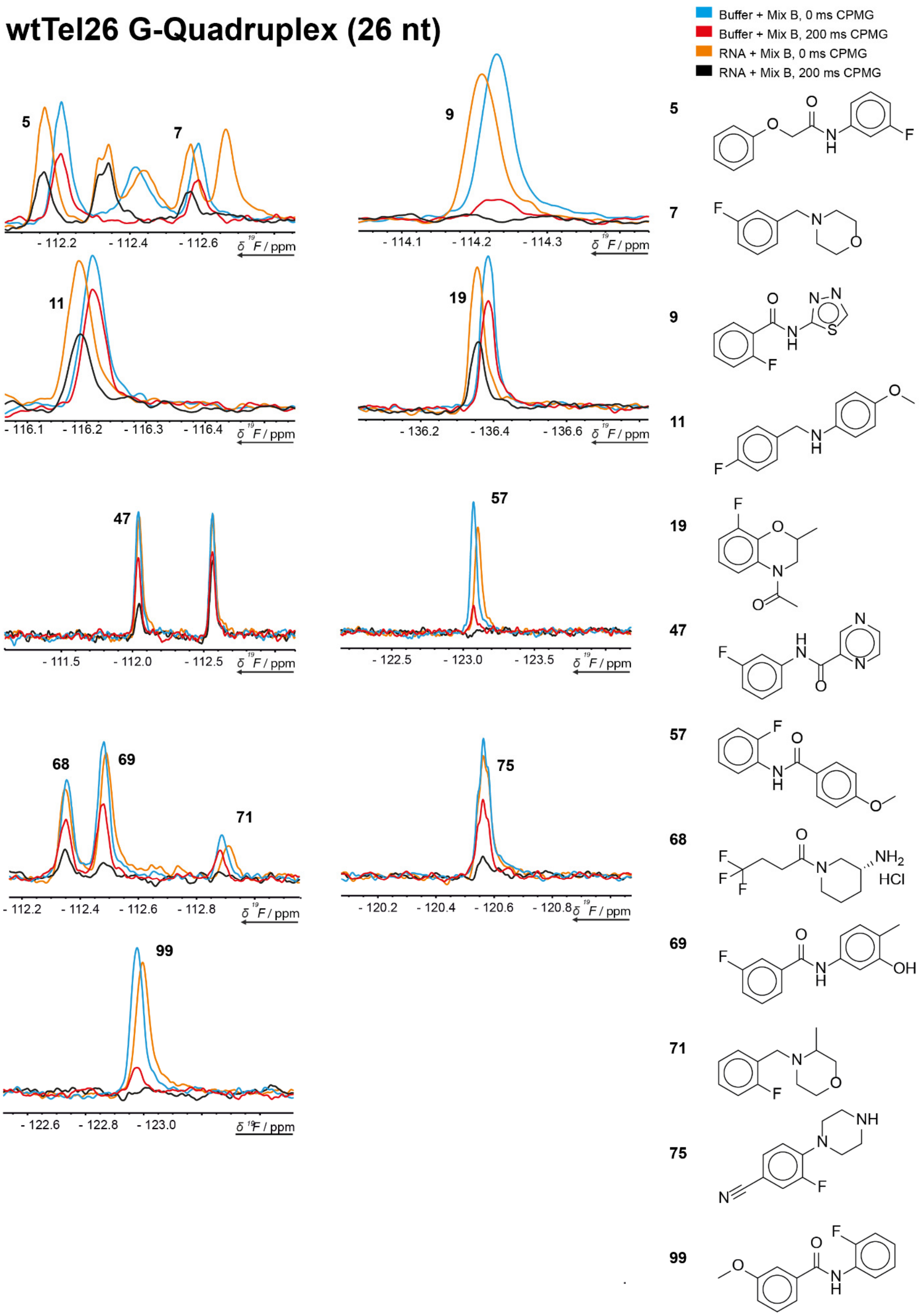

Supplementary Figure 31: Spectral excerpts displaying the intensity modulation obtained in the 200 ms CPMG experiment against 0 ms CPMG identifying the respective fragments as target hits for the wtTel26 G-Quadruplex (26 nt).

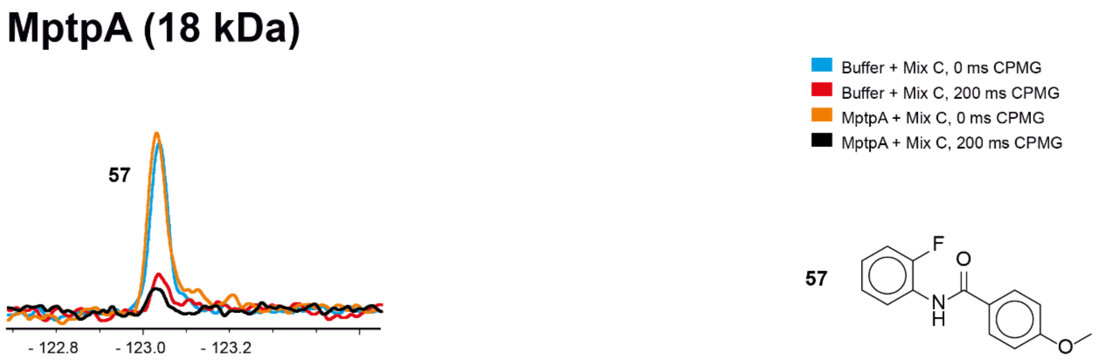

Supplementary Figure 32: Spectral excerpts displaying the intensity modulation obtained in the 200 ms CPMG experiment against 0 ms CPMG identifying the respective fragments as target hits for the Mycobacterium tuberculosis Protein Tyrosine Phosphatase A (MptpA, 18 kDa).

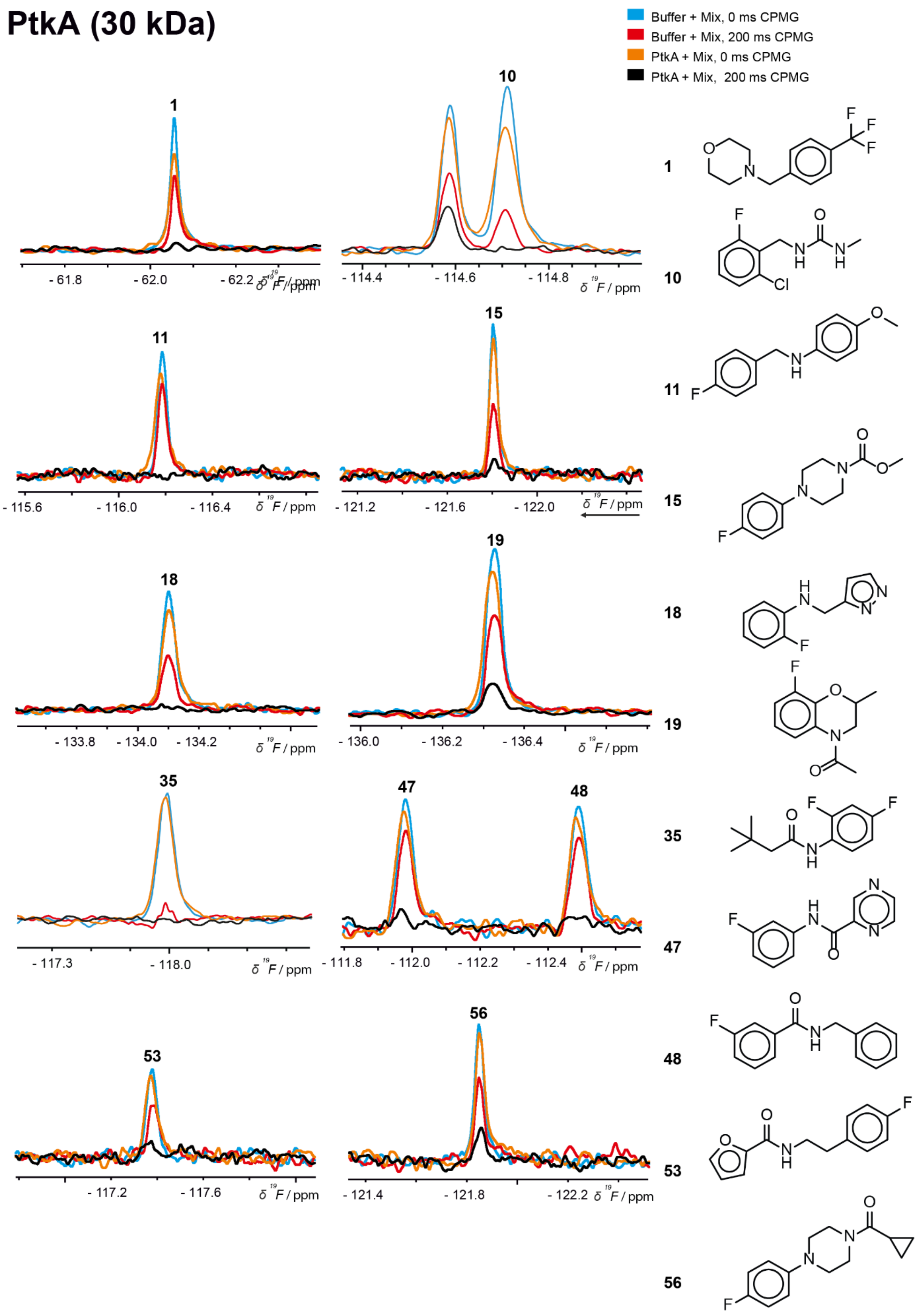

Supplementary Figure 33: Spectral excerpts displaying the intensity modulation obtained in the 200 ms CPMG experiment against 0 ms CPMG identifying the respective fragments as target hits for the Protein tyrosine Kinase A (PtkA, 30 kDa).

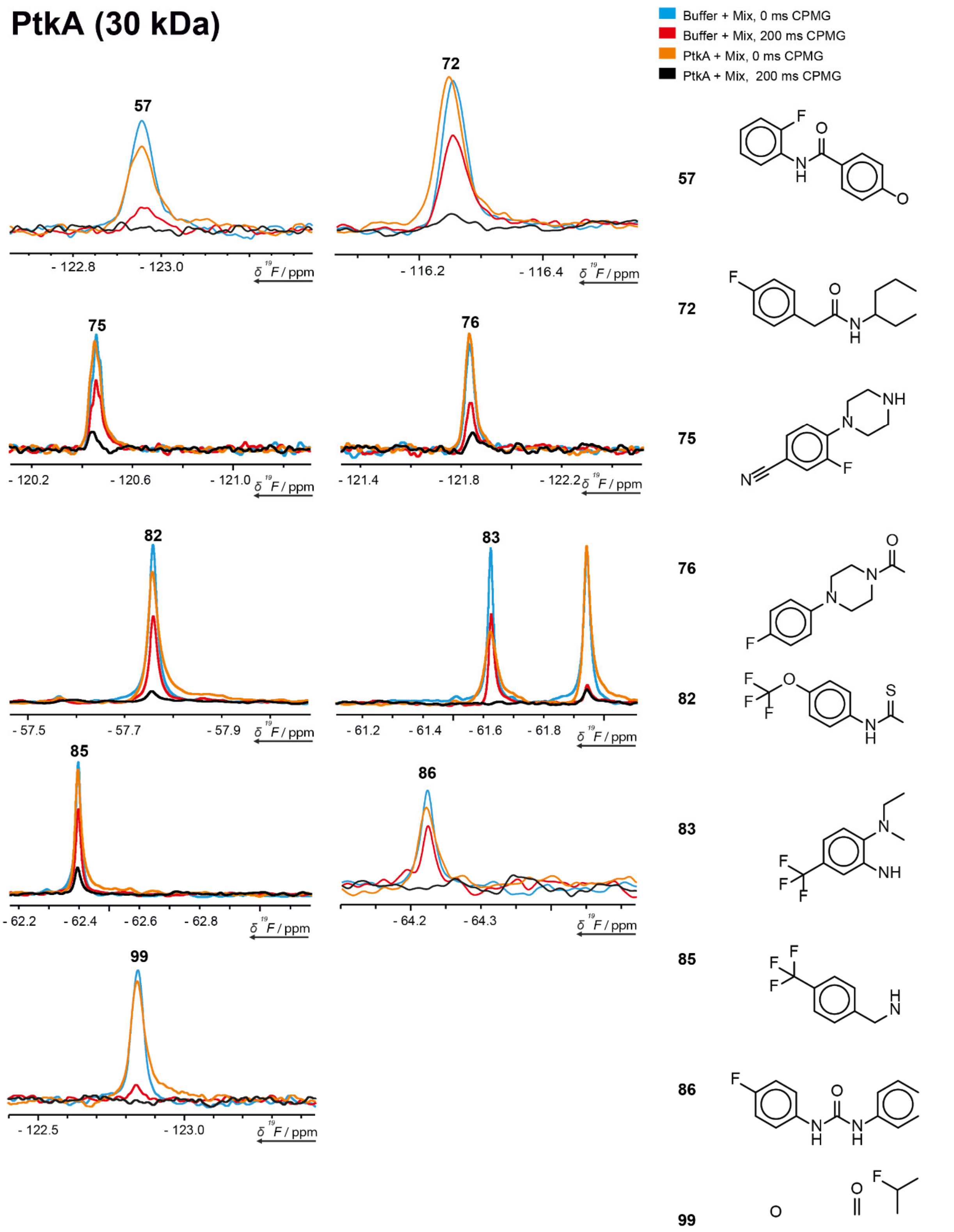

Supplementary Figure 34: Spectral excerpts displaying the intensity modulation obtained in the 200 ms CPMG experiment against 0 ms CPMG identifying the respective fragments as target hits for the Mycobacterium tuberculosis Protein Tyrosine Phosphatase A (MptpA, 18 kDa).

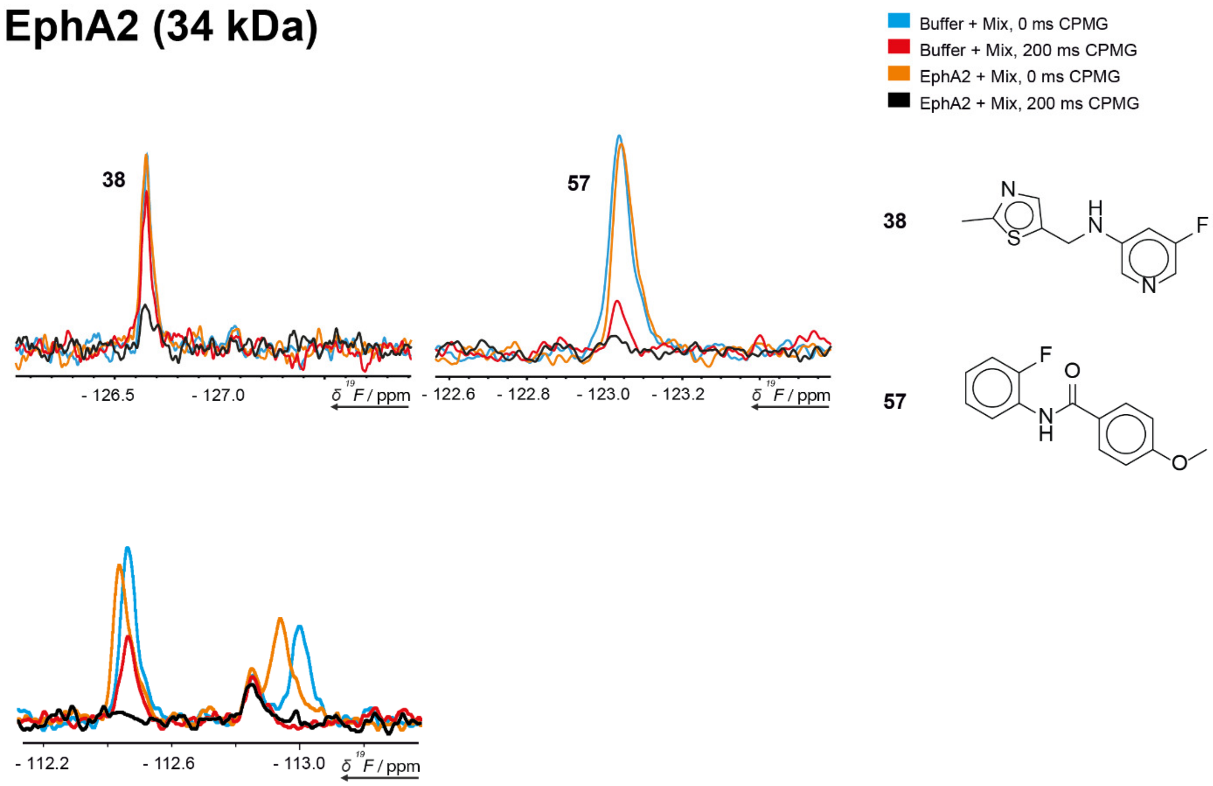

Supplementary Figure 35: Spectral excerpts displaying the intensity modulation obtained in the 200 ms CPMG experiment against 0 ms CPMG identifying the respective fragments as target hits for the Receptor tyrosine kinase EphA2 (61 kDa).

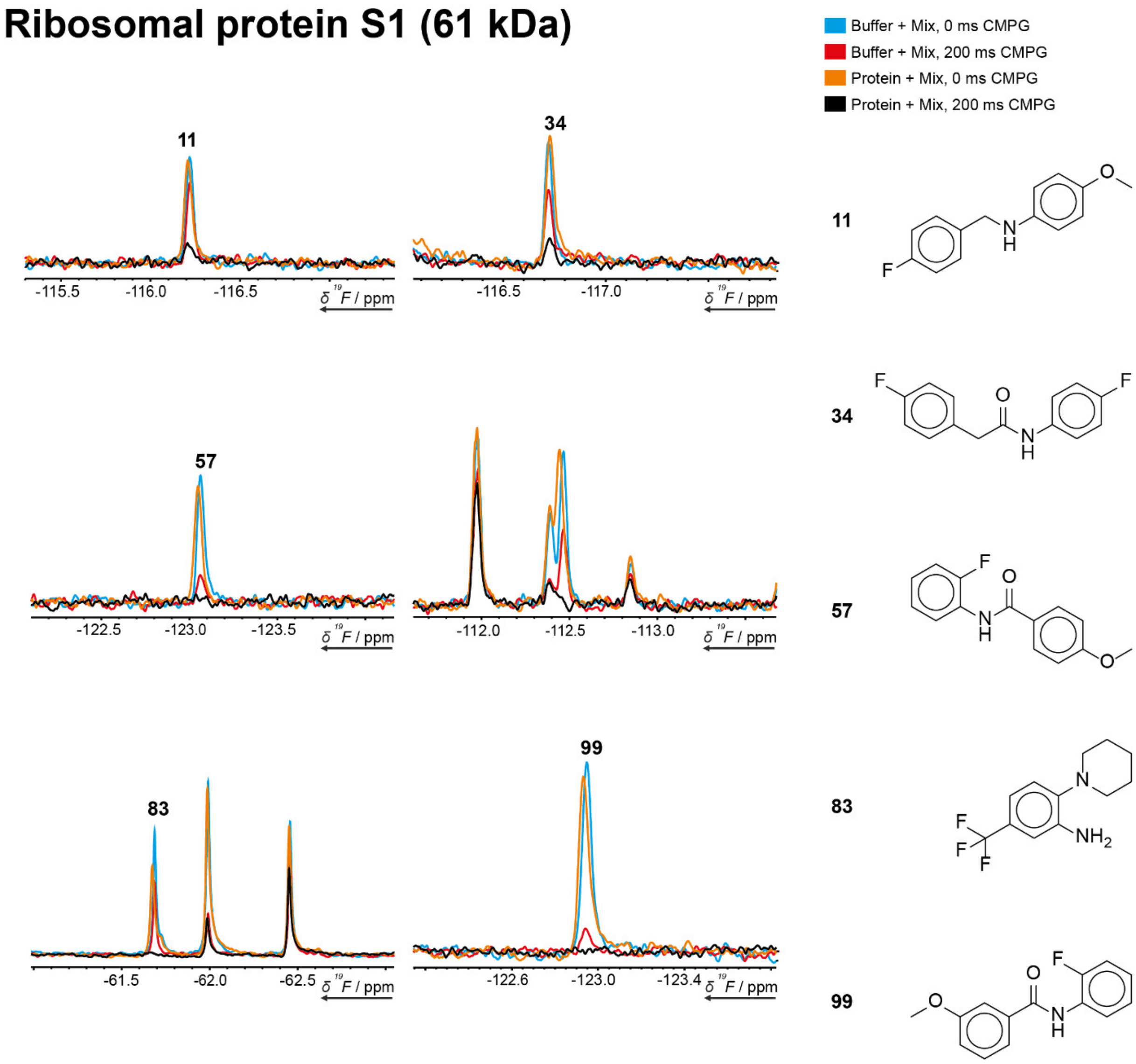

Supplementary Figure 36: Spectral excerpts displaying the intensity modulation obtained in the 200 ms CPMG experiment against 0 ms CPMG identifying the respective fragments as target hits for the Ribosomal protein S1 (61 kDa).

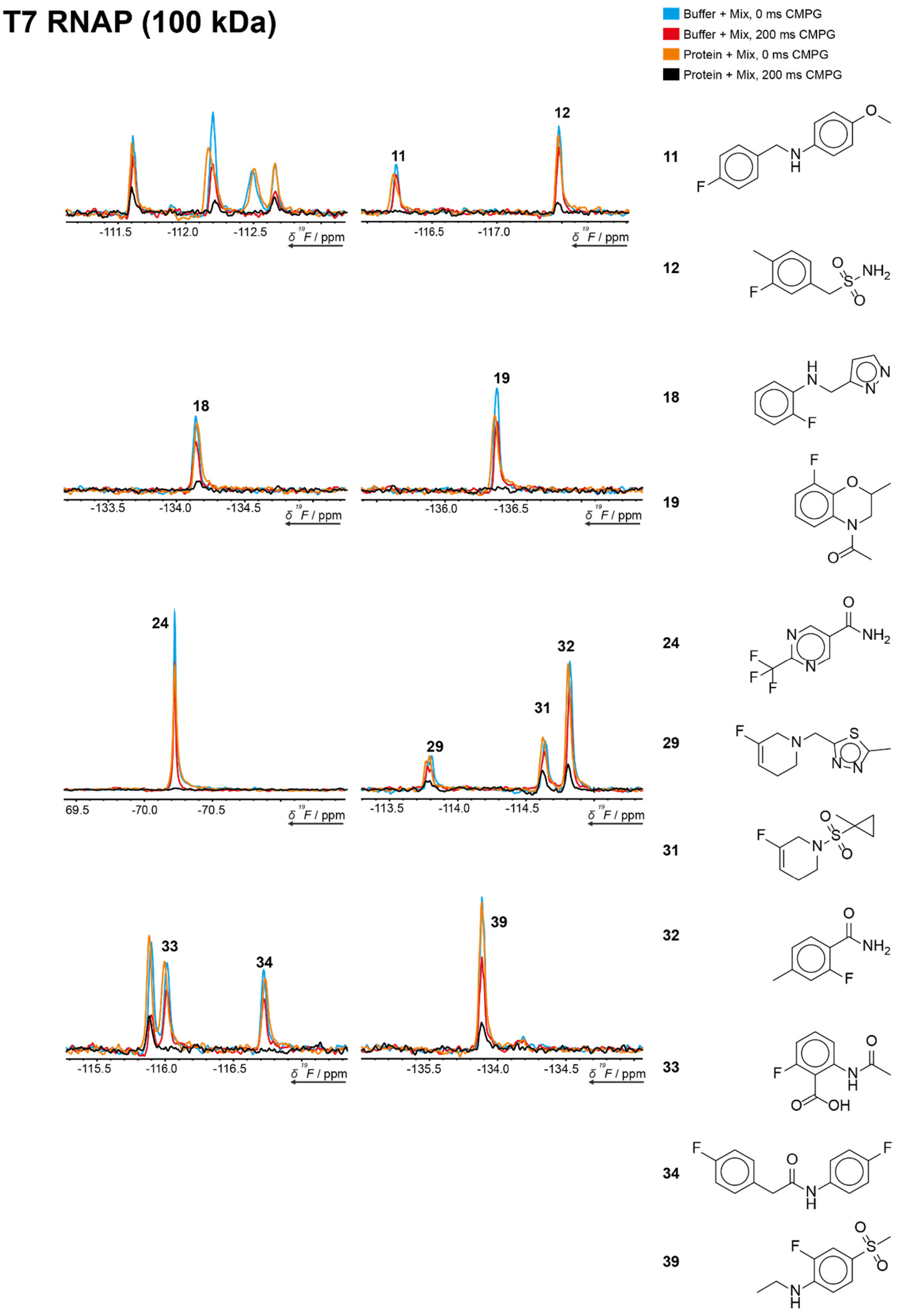

Supplementary Figure 37: Spectral excerpts displaying the intensity modulation obtained in the 200 ms CPMG experiment against 0 ms CPMG identifying the respective fragments as target hits for the T7 RNA polymerase (100 kDa).

T7 RNAP (100 kDa)

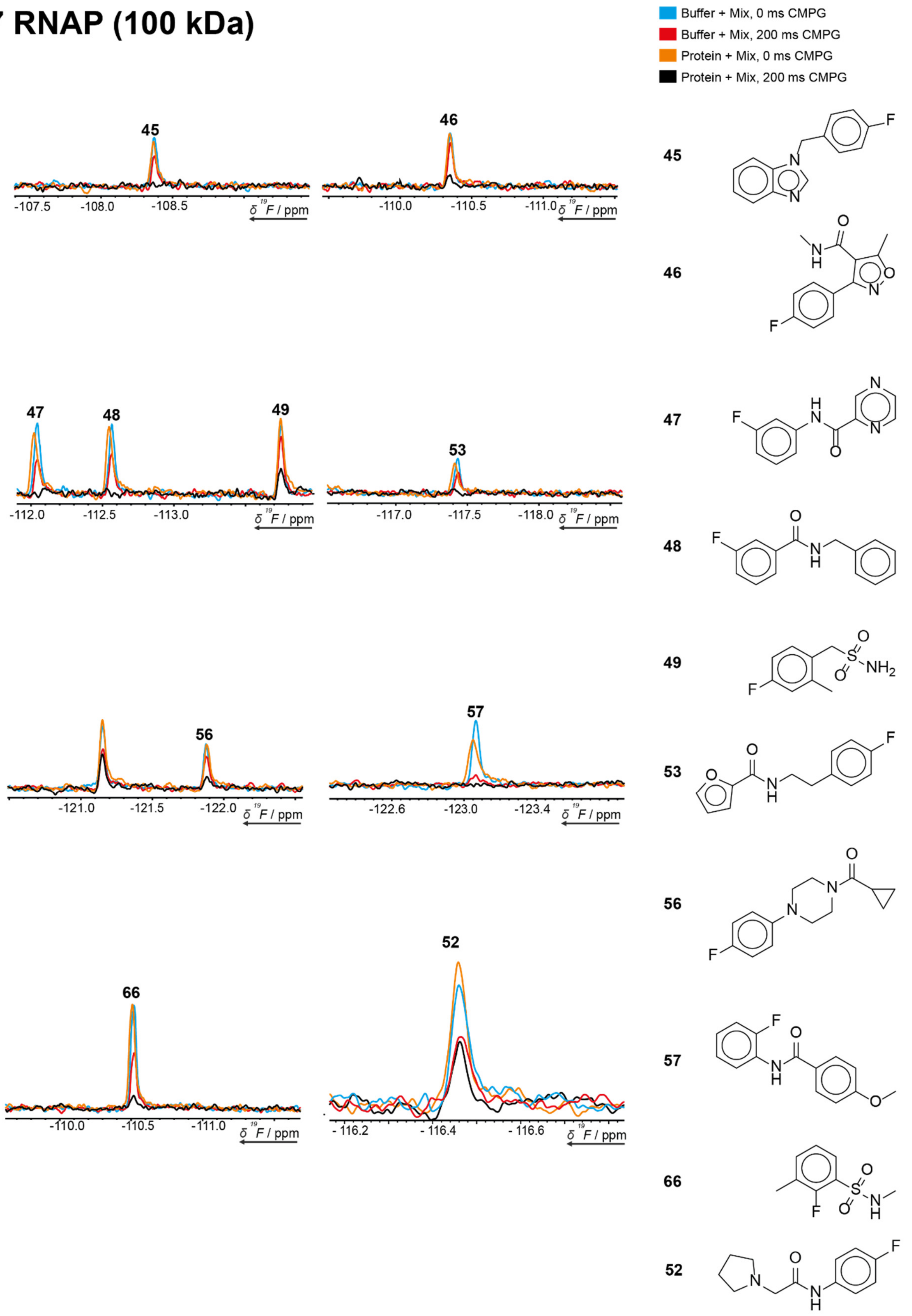

Supplementary Figure 38: Spectral excerpts displaying the intensity modulation obtained in the 200 ms CPMG experiment against 0 ms CPMG identifying the respective fragments as target hits for the T7 RNA polymerase (100 kDa).

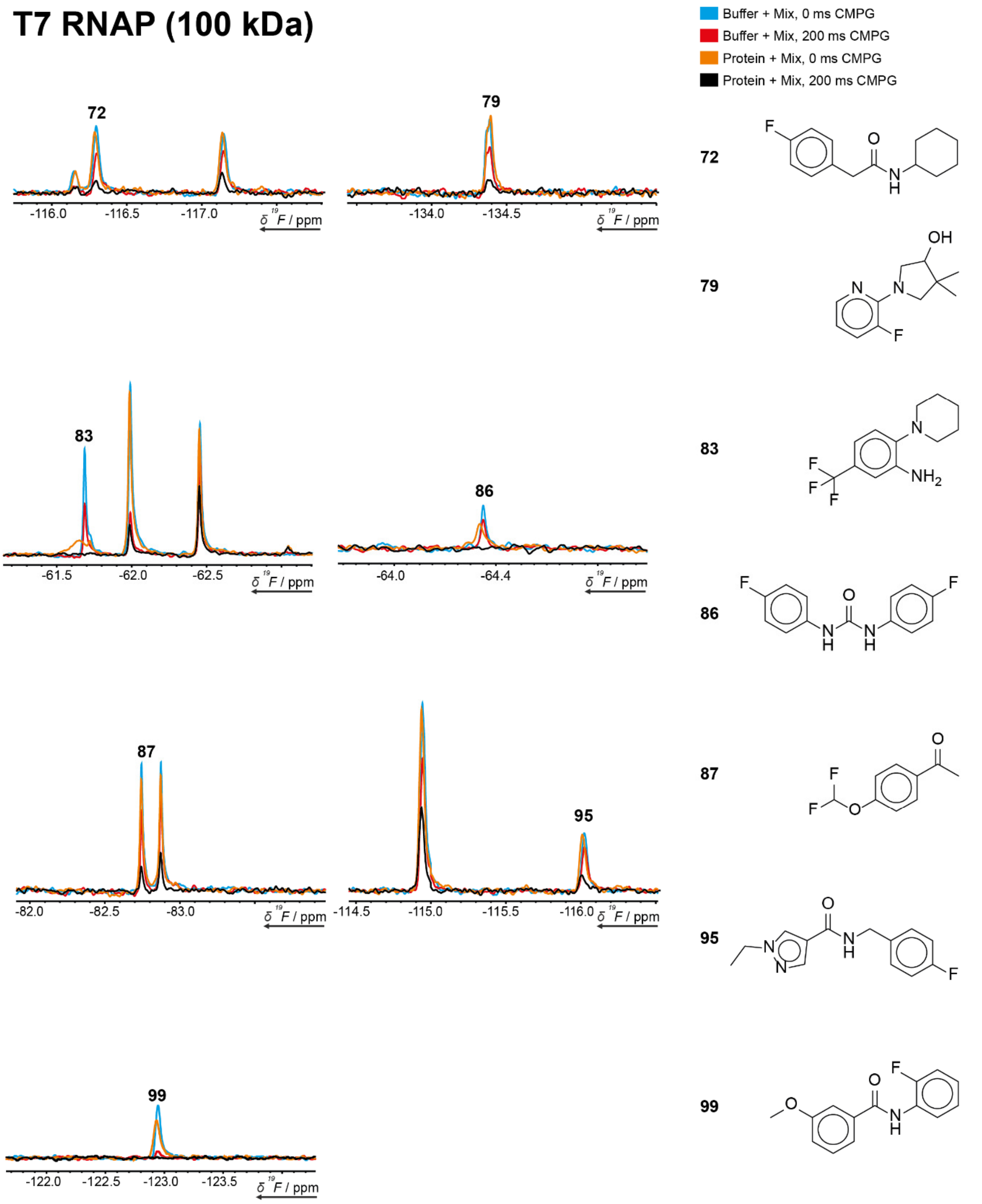

Supplementary Figure 39: Spectral excerpts displaying the intensity modulation obtained in the 200 ms CPMG experiment against 0 ms CPMG identifying the respective fragments as target hits for the T7 RNA polymerase (100 kDa).

Follow up Chemistry

We report here the general strategy for targeting the terminator and antiterminator structural elements found in riboswitches acting at the level of transcription. As a proof of concept, we chose the 39 nt terminator stem as drug target, since it represents the smallest RNA structure containing a druggable bulge motif. For technical reasons, we complemented the screening of the ^{19}F-library with a ^{1}H-library covering 768 analyzed fragments. Aside from the 6 weak and 2 strong hit found in ^{19}F screening, we found 27 additional hits via ^{1}H screening. For the ease of chemistry and commercial availability and affordability of precursors, we focused on a fragment hit containing a benzamide (P2D11) for further modification, which is a close homologue of fragment 48, which showed effect during the ^{19}F-screening. This fragment was linked to an acridine moiety to enhance to binding affinity. Furthermore, the fluorescence of acridine enables fluorescence-based binding assays (Supplementary Figure 56 - Supplementary Figure 61). We performed fluorescence titration assays of the coupled derivative with the herein investigated 39 nt terminator stem. In order to assess selectivity variations towards different RNA structures, we further included a 38 nt antiterminator stem with a large loop as putative drug binding site and a 51 nt terminator stem containing an internal loop. The coupled fragment exhibited a K_D of 15.3 µM towards the 39 nt terminator stem, illustrating that low µM affinity can be established by initial hit modification. Moreover, the coupled fragment showed up to a ~15-fold selectivity for different RNA structures (K_D (38 nt) = 1.1 µM; K_D (51 nt) = 1.5 µM).

Synthesis

General experimental procedures

Reactions were conducted under inert conditions if necessary, glassware was dried beforehand. Starting materials were commercially available and used without further purification. $POCl_3$ und $SOCl_2$ were distilled before usage. Anhydrous solvents were purchased in crown-capped bottles under argon atmosphere, 4 Å molecular sieves were used for storage when needed.

Alugram® Xtra Sil G UV254 silica gel plates from *Macherey-Nagel* were used for performing thin layer chromatography (TLC). For column chromatography silica gel (Silica 60, 0.04-0.063 mm) from *Macherey-Nagel* was used.

Mass spectra were recorded on a *Thermo Fisher* Surveyor MSQ spectrometer.

All shown NMR-spectra were recorded at room temperature using on an AV500HD or DRX-600 spectrometer from *Bruker*. Calibration of the NMR spectra were done by referencing the chemical shift values to residual solvent signals DMSO-d_6: δ (^{1}H) = 2.50 ppm, δ (^{13}C) = 39.52 ppm. Following abbreviations were used: s = singlet, d = doublet, dd = doublet of doublet, t = triplet, q = quartet, m = multiplet, b = broad.

Synthesis and analytics

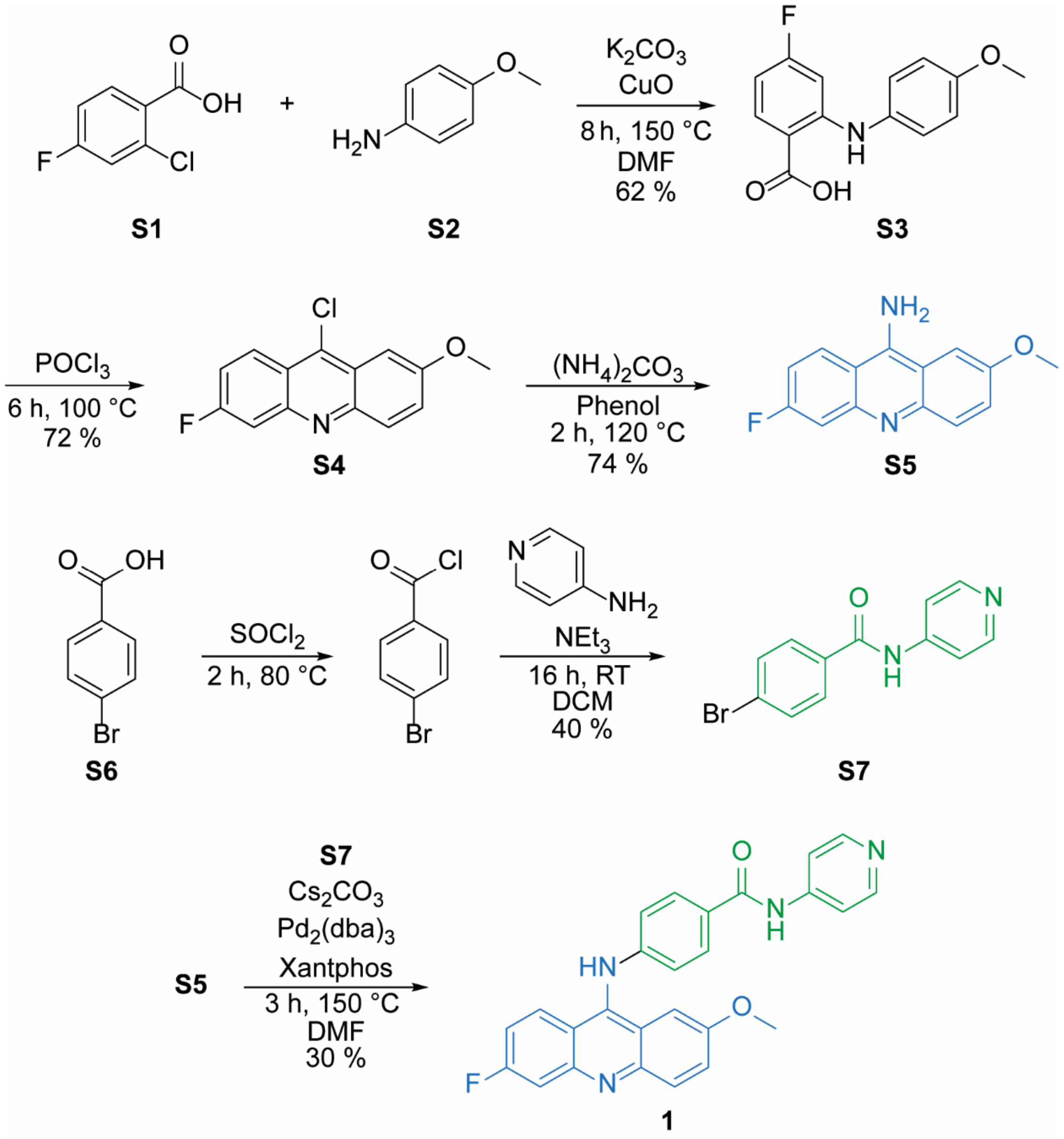

Supplementary Figure 40: Synthesis route of compound 1.

4-Fluoro-2-((4-methoxyphenyl)amino)benzoic acid (S3)

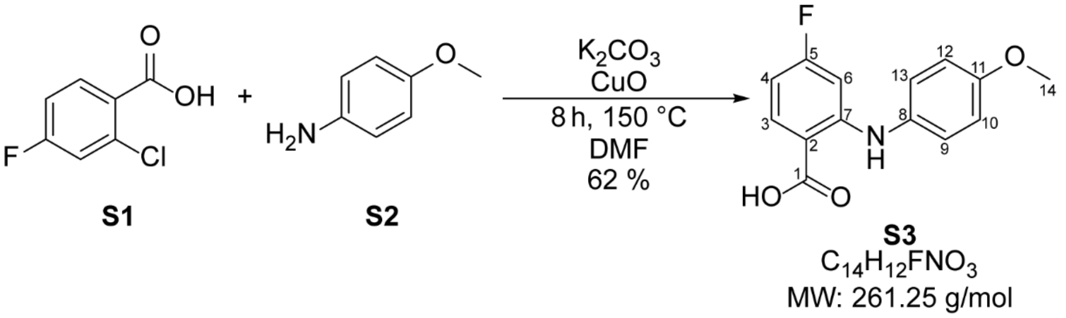

The synthesis of **S3** was carried out according to a procedure from Haider *et al.*[1] for the synthesis of 2-(phenylamino)benzoic acid. Potassium carbonate (6.33 g, 45.8 mmol) was dried in oil-pump vacuum for 30 min, followed by the addition of **S1** (4.00 g, 22.9 mmol), **S2** (3.10 g, 25.2 mmol) and copper(II) oxid (0.73 g, 9.2 mmol). All solid starting materials were dried for additional 10 min in oil-pump vacuum and were suspended in dry. DMF (10 mL). The reaction mixture was heated to reflux for 8 h. After stirring at room temperature overnight the mixture was diluted with H_2O and filtered over celite. The filtrate was acidified with hydrochloric acid. The formed precipitate was collected by filtration and washed with H_2O. The solid was dissolved in EtOAc and the acidic filtrate was extracted three times with EtOAc. The organic layers were combined and dried over $MgSO_4$, filtered and the solvent was removed under reduced pressure. The crude product was purified by column chromatography (silica gel, Cy:EtOAc = 1:3). The product was obtained as a brown solid (3.75 g, 62%).

R_f = 0.85 (silica gel, Cy:EtOAc = 1:3).

MS (ESI-): m/z calculated: [M] = 261.25; measured: $[M-H]^-$ = 259.99.

^{1}H-NMR: (500.18 MHz, DMSO-d_6) δ [ppm] = 13.06 (bs, 1H, COOH); 9.64 (bs, 1H, NH); 7.93 (dd, 1 H, J = 8.8 Hz, J = 7.1 Hz, Pos. 3); 7.23 – 7.19 (m, 2H, Pos. 9 & 13); 7.00 – 6.97 (m, 2H, Pos. 10 & 12); 6.53 – 6.47 (m, 2H, Pos. 4 & 6); 3.77 (s, 3H, Pos. 14).

^{13}C-NMR: (125.77 MHz, DMSO-d_6) δ [ppm] = 169.3 (Pos. 1); 165.9 (d, $^1J_{CF}$ = 248.8 Hz, Pos. 5); 156.6 (Pos. 11); 151.2 (d, $^3J_{CF}$ = 12.2 Hz, Pos. 7); 134.8 (d, $^3J_{CF}$ = 11.6 Hz, Pos. 3); 132.0 (Pos. 8); 125.7 (Pos. 9 & 13); 114.8 (Pos. 10 & 12); 108.0 (Pos. 2); 103.6 ($^2J_{CF}$ = 22.6 Hz, Pos. 4 or Pos. 6); 98.3 ($^2J_{CF}$ = 26.3 Hz, Pos. 4 or Pos. 6); 55.3 (Pos. 14).

^{19}**F-NMR:** (470.64 MHz, DMSO-*d*$_6$) δ [ppm] = -104.25 – -104.31 (m, Pos. 9).

9-Chloro-6-fluoro-2-methoxyacridine (S4)

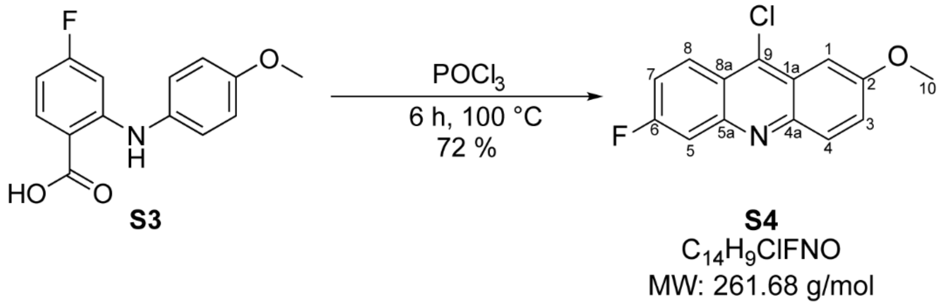

S4 was synthesized according a procedure of Mohammadi-Khanaposhtani *et al.*[2] for the synthesis of 6,9-dichloro-2-methoxyacridine. **S3** (3.75 g, 14.4 mmol) was suspended in freshly distilled $POCl_3$ (15.00 mL, 164.35 mmol) and heated to reflux for 6 h. The mixture was carefully poured under rigorous stirring on ice water. The pH was adjusted to 11 with aqueous NH_3 solution after the ice melted. The precipitate was removed by filtration and the filtrate was extracted five times with DCM. The combined organic layers were dried over $MgSO_4$, filtered and the solvent was removed under reduced pressure. The crude product was purified by column chromatography (silica gel, Cy:EtOAc = 4:1). Desired product was obtained as a yellow solid (2.68 g, 72%).

$\mathbf{R_f}$ = 0.61 (silica gel, Cy:EtOAc = 1:3).

MS (ESI+): m/z calculated: [M] = 261.68; measured: $[M\text{-}H]^+$ = 261.98.

^{1}**H-NMR:** (500.18 MHz, DMSO-*d*$_6$) δ [ppm] = 8.44 (dd, 1H, J = 9.5 Hz, J = 6.2 Hz, Pos. 8); 8.09 (d, 1H, $^3J_{HH}$ = 9.4 Hz, Pos. 4); 7.90 (dd, 1H, $^3J_{HF}$ = 10.3 Hz, $^4J_{HH}$ = 2.5 Hz, Pos.5); 7.74 – 7.70 (m, 1H, Pos. 7); 7.62 (dd, $^3J_{HH}$ = 9.5 Hz, $^4J_{HH}$ = 2.8 Hz, Pos. 3); 7.51 (d, 1H, $^4J_{HH}$ = 2.8 Hz, Pos. 1); 4.02 (s, 3H, Pos. 10).

^{13}**C-NMR:** (125.77 MHz, DMSO-*d*$_6$) δ [ppm] = 162.3 (d, $^1J_{CF}$ = 250.1 Hz, Pos. 6); 158.1 (Pos. 2); 147.1 (d, $^3J_{CF}$ = 13.4 Hz, Pos. 5a); 146.3 (Pos. 4a); 137.5 (Pos. 9); 131.1 (Pos. 4); 126.9 (d, $^3J_{CF}$ = 10.7 Hz, Pos. 8); 126.7 (Pos. 3); 124.2 (Pos. 1a); 121.2 (Pos. 8a); 119.2 ($^2J_{CF}$ = 27.6 Hz, Pos. 7); 111.8 ($^2J_{CF}$ = 20.2 Hz, Pos. 5); 99.7 (Pos. 1); 55.8 (Pos.10).

^{19}**F-NMR:** (470.64 MHz, DMSO-*d*$_6$) δ [ppm] = -108.93 – -109.98 (m, Pos. 6)

9-Amino-6-fluoro-2-methoxyacridine (S5)

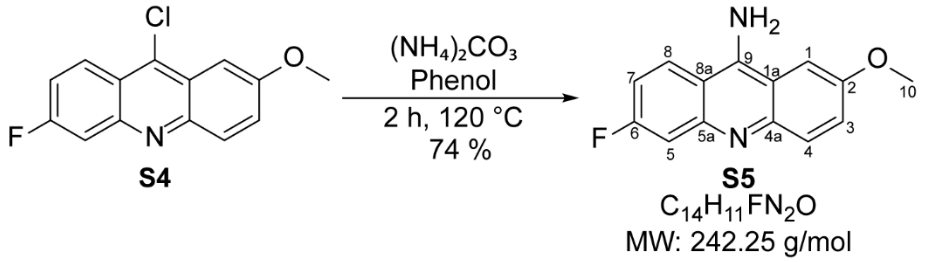

$C_{14}H_{11}FN_2O$
MW: 242.25 g/mol

The following synthesis was performed according to the synthesis von 9-amino-6-chlor-2-methoxyacridine by Bonse *et al.*[3]. **S4** (1.50 g, 5.7 mmol), phenol (6.00 g, 63.7 mmol) and ammonium carbonate (0.94 g, 9.7 mmol) were dried in oil-pump vacuum and then heated to 120 °C for 2 h. The mixture was cooled to room temperature and 5 M aqueous NaOH solution was added until pH 12. After separating the precipitate by filtration, it was washed with 1 M aqueous NaOH and H_2O and then dissolved in EtOAc. The aqueous layer was extracted with EtOAc and the combined organic layers were dried over $MgSO_4$ and filtered. After removing the solvent the crude product was purified by column chromatography (silica gel, Cy:EtOAc = 1:1 to DCM:MeOH = 4:1). The product was obtained as a yellow solid (1.02 g, 72%).

R_f = 0.74 (silica gel, DCM:MeOH = 4:1).

MS (ESI-): m/z calculated: [M] = 242.25; measured: $[M-H]^+$ = 243.04.

^{1}H-NMR: (500.18 MHz, DMSO-d_6) δ [ppm] = 8.44 (dd, 1H, J = 9.4 Hz, J = 6.5 Hz, Pos. 8); 7.74 (d, 1H, $^3J_{HH}$ = 9.0 Hz, Pos. 4); 7.67 – 7.66 (m, 3H, Pos. 1 & NH_2); 7.44 (dd, $^3J_{HF}$ = 11.6 Hz, $^4J_{HH}$ = 2.4 Hz, Pos. 5); 7.36 (dd, $^3J_{HH}$ = 9.3 Hz, $^4J_{HH}$ = 2.3 Hz, Pos. 3); 7.24 – 7.21 (m, 1H, Pos. 7); 3.91 (s, 3H, Pos. 10).

^{13}C-NMR: (125.77 MHz, DMSO-d_6) δ [ppm] = 162.4 (d, $^1J_{CF}$ = 246.6 Hz, Pos. 6); 154.3 (Pos. 2); 148.8 (Pos. 9); 148.4 (d, $^3J_{CF}$ = 13.1 Hz Pos. 5a); 146.0 (Pos. 4a); 130.2 (Pos. 4); 126.1 (d, $^3J_{CF}$ = 11.3 Hz, Pos. 8); 124.0 (Pos. 3); 112.9 (Pos. 1a); 112.4 (d, $^2J_{CF}$ = 25.8 Hz, Pos. 7); 110.5 (d, $^2J_{CF}$ = 19.3 Hz, Pos. 5); 110.2 (Pos. 8a); 100.4 (Pos. 1) 55.7 (Pos. 10).

^{19}F-NMR: (470.64 MHz, DMSO-d_6) δ [ppm] = -111.68 – -111.62 (m, Pos. 6)

4-Bromo-*N*-4-pyridinylbenzamide (S7)

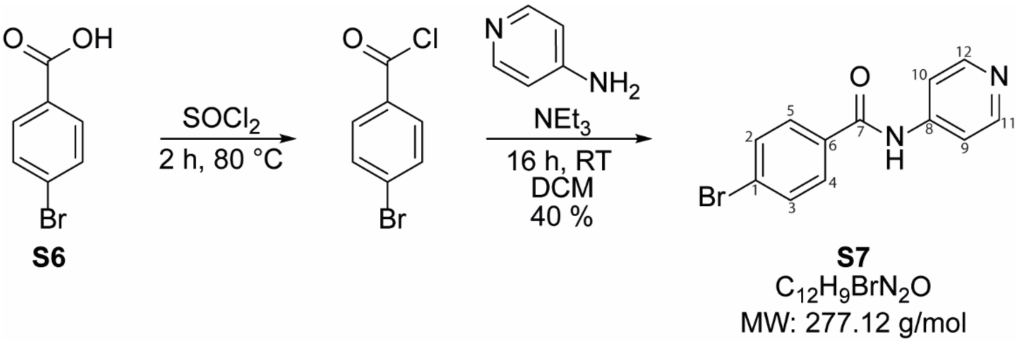

S6 (0.50 g, 2.3 mmol) was dried in oil-pump vacuum for 15 min, suspended in fresh distilled $SOCl_2$ (3.1 mL, 42 mmol) and heated to reflux until the suspension turned to a clear solution. After cooling to room temperature excessive $SOCl_2$ was removed under reduced pressure and the resulting solid was dried for 1 h in oil-pump vacuum. The solid was dissolved in dry DCM (2 mL), following by the addition of NEt_3 (0.65 mL, 4.6 mmol) under cooling. A solution of 4-aminopyridine (0.22 g, 2.28 mmol) in a mixture of dry DCM (15.00 mL) and dry DMF (1.50 mL) was added dropwise under cooling. The reaction mixture was stirred at room temperature overnight. The reaction was stopped by addition of H_2O and subsequently the aqueous layer was extracted with EtOAc. The combined organic layers were dried over $MgSO_4$, filtered and the solvent removed under reduced pressure. The crude product was purified by column chromatography (silica gel, Cy:EtOAc = 1:1). Since residues of **S6** were observed after column chromatography, the obtained solid was dissolved in 1 M NaOH and the aqueous layer extracted with EtOAc. After removing the solvent under reduced pressure, the product was obtained as a white solid (251 mg, 40%).

R_f = 0.61 (silica gel, Cy:EtOAc = 1:3).

MS (ESI-): m/z calculated: [M] = 277.12; measured: $[M\text{-}H]^-$ = 276.93.

^{1}H-NMR: (500.18 MHz, DMSO-*d*$_6$) δ [ppm] = 10.64 (bs, 1H, NH); 8,49 – 8.47(m, 2H, Pos. 11 & 12); 7.93 - 7.90 (m, 2H, Pos. 4 & 5), 7.79 – 7.76 (m, 4H, Pos. 2, 3, 9 & 10).

^{13}C-NMR: (125.77 MHz, DMSO-*d*$_6$) δ [ppm] = 165.5 (Pos. 7); 150.3 (Pos. 11 &12); 145.7 (Pos. 8); 133.3 (Pos. 4); 132.5 (Pos. 2 & 3); 129.9 (Pos. 4 & 5); 126.0 (Pos. 1); 114.0 (Pos. 9 & 10).

4-[6-Fluoro-2-methoxy-9-acridinyl)amino]-*N*-4-pyridinylbenzamide (1)

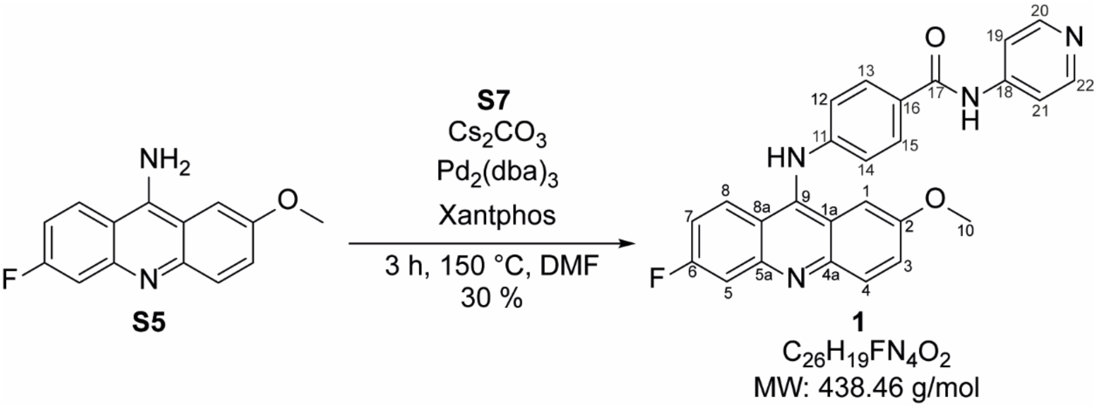

The synthesis of **1** was performed according to a procedure of Gellerman *et al.*[4] for the synthesis of functionalized aminoacridine derivatives. **S5** (110.0 mg, 454.1 µmol), **S7** (125.8 mg, 454.1 µmol) and Cs_2CO_3 (74 mg, 227 µmol) were dried in oil-pump vacuum. The solids were then suspended in dry DMF (4 mL) and heated to 90 °C for 7 h, following by heating for 4 h to reflux. However, no product formation could be observed by TLC. Therefore, Cs_2CO_3 (518 mg, 1.59 mmol), $Pd_2(dba)_3$ (37.1 mg, 40.9 µmol) and Xantphos (39.3 mg, 68.1 µmol) were added to the reaction mixture. The mixture was heated to reflux for 3 h. The solvent was removed by warming to 40 °C under oil-pump vacuum. The crude product was suspended in MeOH and filtered over celite. After the removal of MeOH, the crude product was purified by column chromatography (silica gel, DCM:MeOH = 9:1). The desired product was obtained as an orange solid (58 mg, 30%).

For analytics via NMR spectroscopy 2 vol.-% of either TFA or HCl was added to the NMR sample, since analyzing the normal sample was not possible due to an aminoacridine-acridanimine tautomerism as described in literature[5].

The isolated product contained two impurities after applying oil-pump vacuum. One of those is DCM and the other could not be identified. ^{1}H and ^{13}C spectra show the presence of these impurities.

R_f = 0.57 (silica gel, DCM/MeOH 9:1).

MS (ESI-): m/z calculated for [M] = 438.46; measured: $[M-H]^-$ = 438.08.

^{1}H-NMR: (500.18 MHz, DMSO-*d_6*) δ [ppm] = 11.94 (s, 1H, Amid-NH); 8,76 (d. 2H, $^{3}J_{HH}$ = 7.3 Hz, Pos. 20 & 22); 8.49 (d, 2H, $^{3}J_{HH}$ = 7.4 Hz, Pos. 19 & 21); 8.27 – 8,21 (m, 4H, Pos. 4, 8, 13 & 15); 8.00 (dd, 1H, $^{3}J_{HF}$ = 9.6 Hz, $^{4}J_{HH}$ = 2.5 Hz, Pos. 5); 7.92

(d, 1H, $^{4}J_{HH}$ = 2.7 Hz, Pos. 1); 7.77 (dd, 1H, $^{3}J_{HH}$ = 9.5 Hz, $^{4}J_{HH}$ = 2.6 Hz, Pos. 3); 7.55 (d, 2H, $^{3}J_{HH}$ = 8.7 Hz, Pos. 12 & 14); 7.44 – 7.39 (m, 1H, Pos. 7); 3.79 (s, 3H, Pos. 10);

^{13}C-NMR: (125.77 MHz, DMSO-*d₆*) δ [ppm] = 166.4 (Pos. 17); 164.7 (d, $^{1}J_{CF}$ = 256.3 Hz, Pos. 6); 156.2 (Pos. 2); 154.8 (Pos. 18); 152.8 (Pos. 9); 146.3 (Pos. 11); 141.9 (Pos. 20 & 22); 140.9 ($^{3}J_{CF}$ = 13.8 Hz, Pos. 5a); 136.6 (Pos. 4a); 130.3 (Pos. 4); 129.8 ($^{3}J_{CF}$ = 11.5 Hz, Pos. 8); 129.2 (Pos. 3 & Pos. 16); 122.4 (Pos. 12 & 14); 121.1 (Pos.13 & 15); 117.1 (Pos. 1a); 115.4 (Pos. 19 & 21); 115.1 ($^{2}J_{CF}$ = 25.9 Hz, Pos. 7); 112,6 (Pos. 8a); 103.8 (Pos. 1); 103.6 ($^{2}J_{CF}$ = 25.1 Hz, Pos. 5); 56.2 (Pos. 10).

^{19}F-NMR: (470.64 MHz, DMSO-*d₆*) δ [ppm] = -100.79 – -100.83 (m, Pos. 6)

NMR-spectra

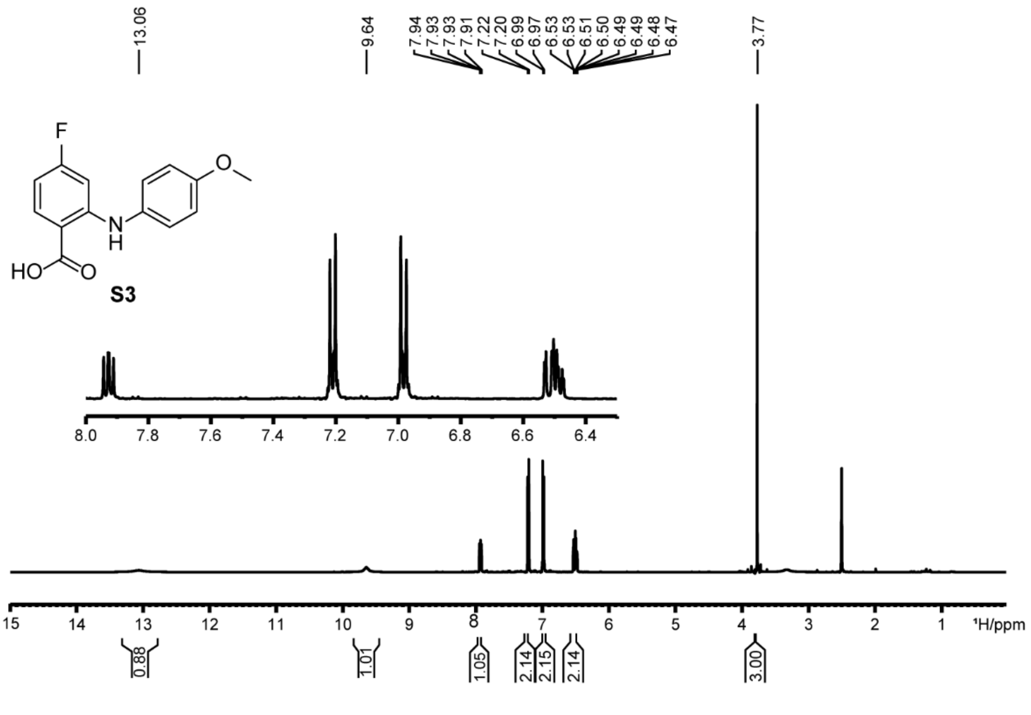

*Supplementary Figure 41: ^{1}H-NMR spectrum of **S3** (DMSO-d₆, 298 K, 500.18 MHz).*

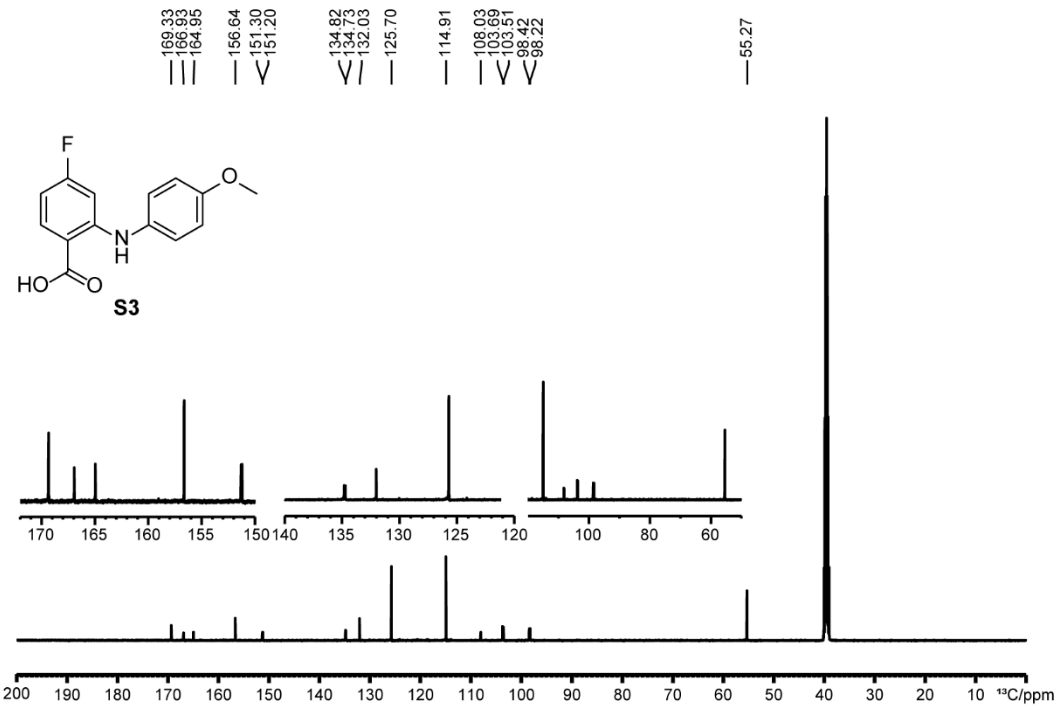

Supplementary Figure 42: ^{13}C-NMR spectrum of S3 (DMSO-d6, 298 K, 157.77 MHz).

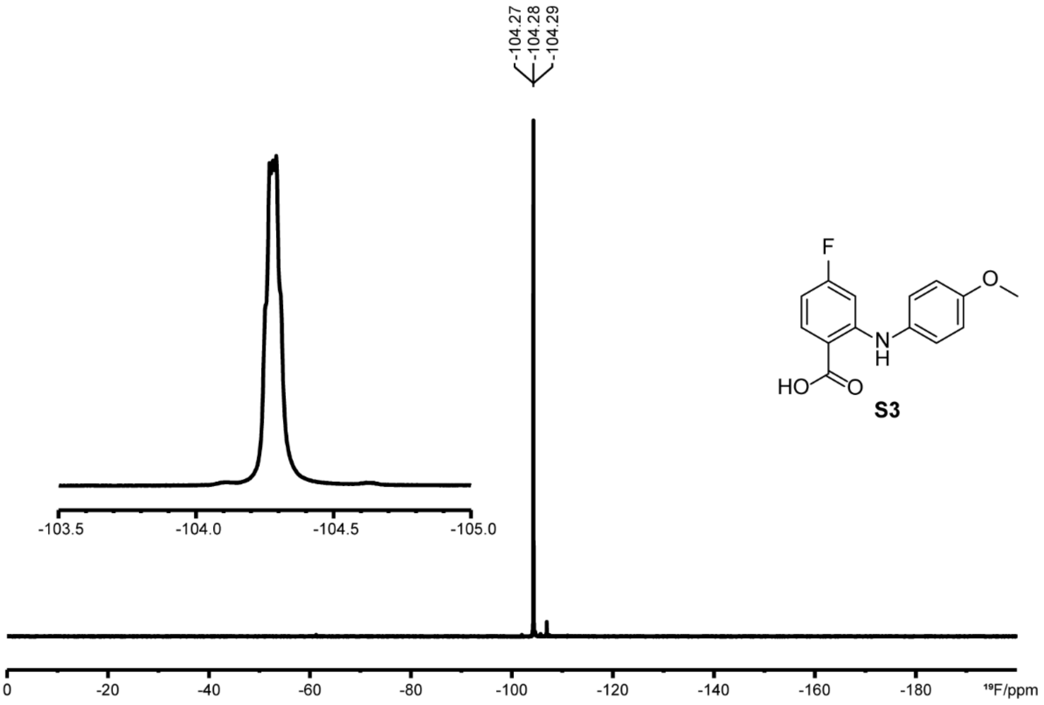

Supplementary Figure 43: ^{19}F-NMR spectrum of S3 (DMSO-d6, 298 K, 470.64 MHz).

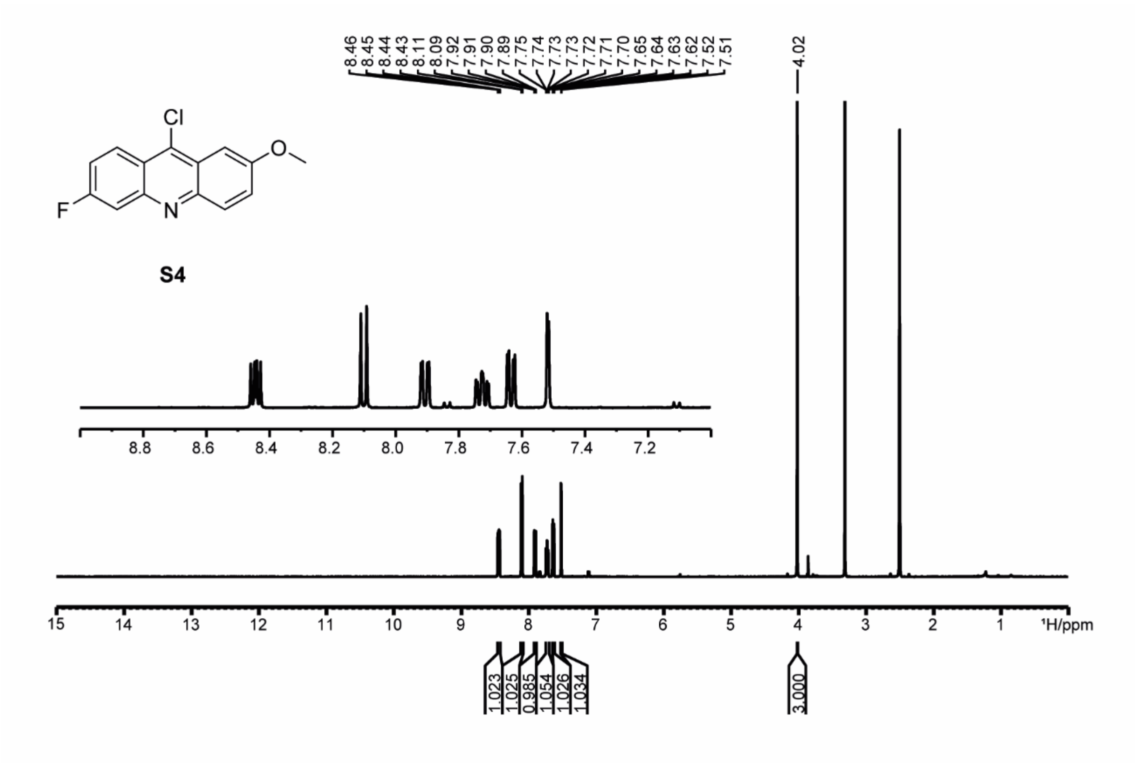

Supplementary Figure 44: ^{1}H-NMR spectrum of S4 (DMSO-d6, 298 K, 500.18 MHz).

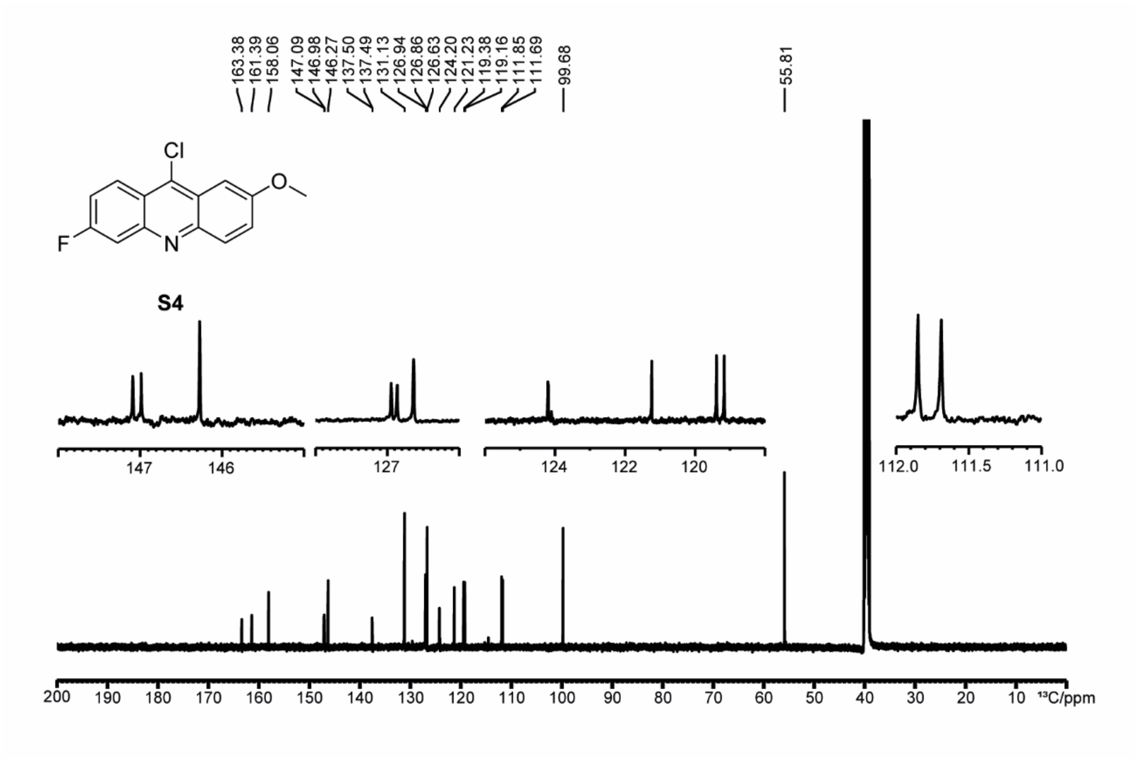

Supplementary Figure 45: ^{13}C-NMR spectrum of S3 (DMSO-d6, 298 K, 157.77 MHz).

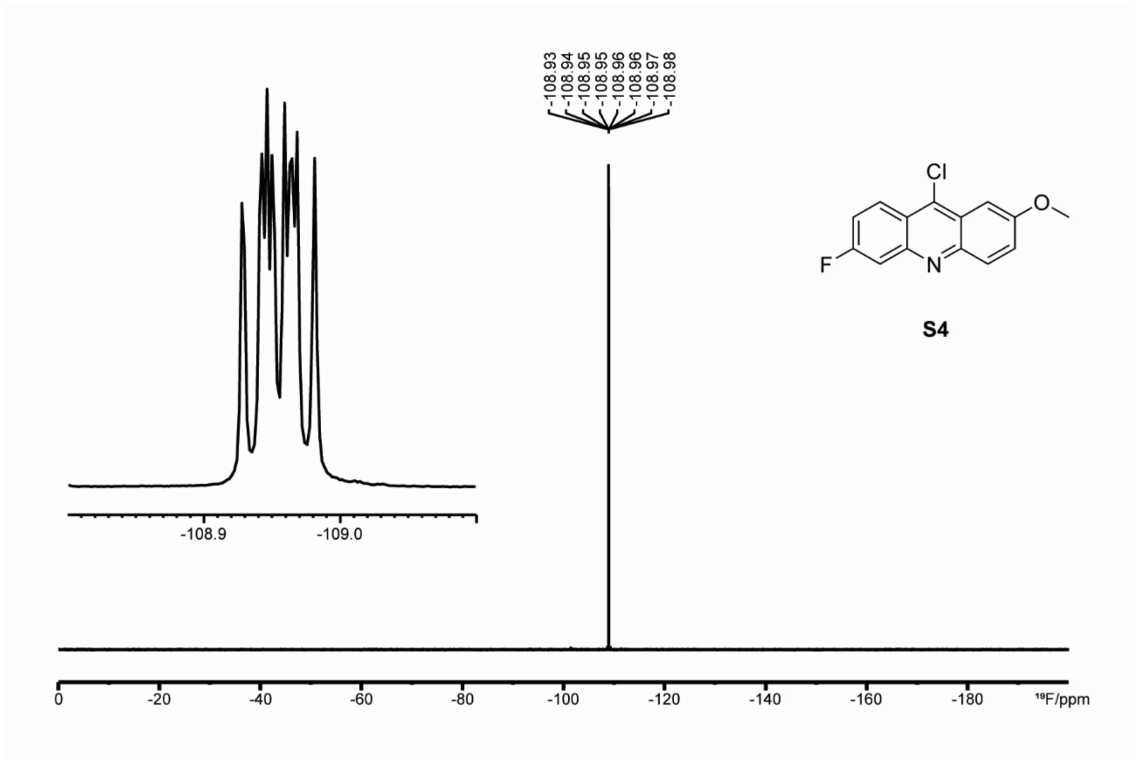

Supplementary Figure 46: ^{19}F-NMR spectrum of S4 (DMSO-d6, 298 K, 470.64 MHz).

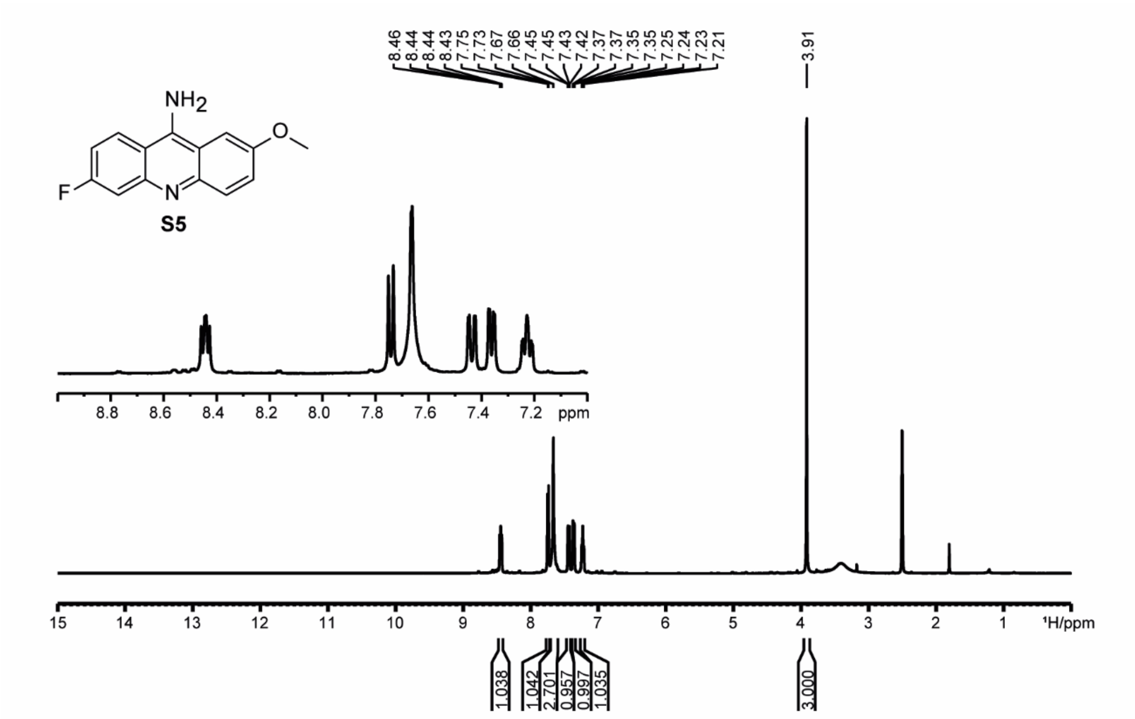

Supplementary Figure 47: ^{1}H-NMR spectrum of S5 (DMSO-d6, 298 K, 500.18 MHz).

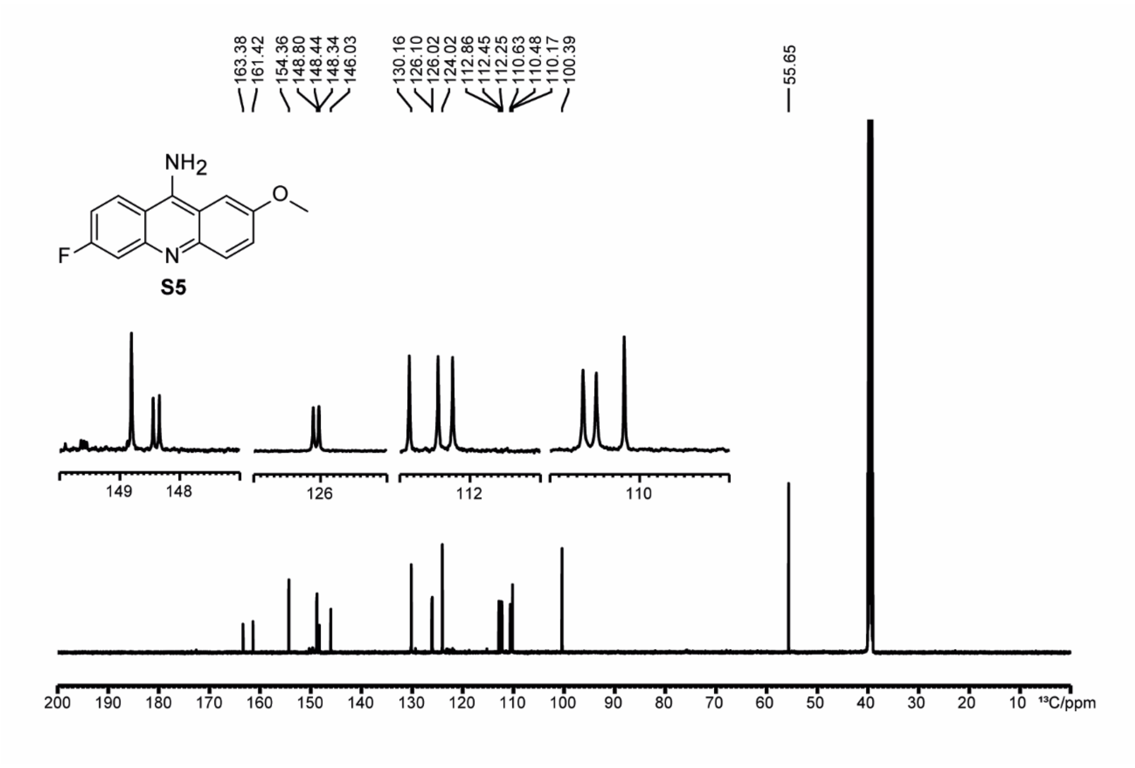

Supplementary Figure 48: ^{13}C-NMR spectrum of S5 (DMSO-d6, 298 K, 157.77 MHz).

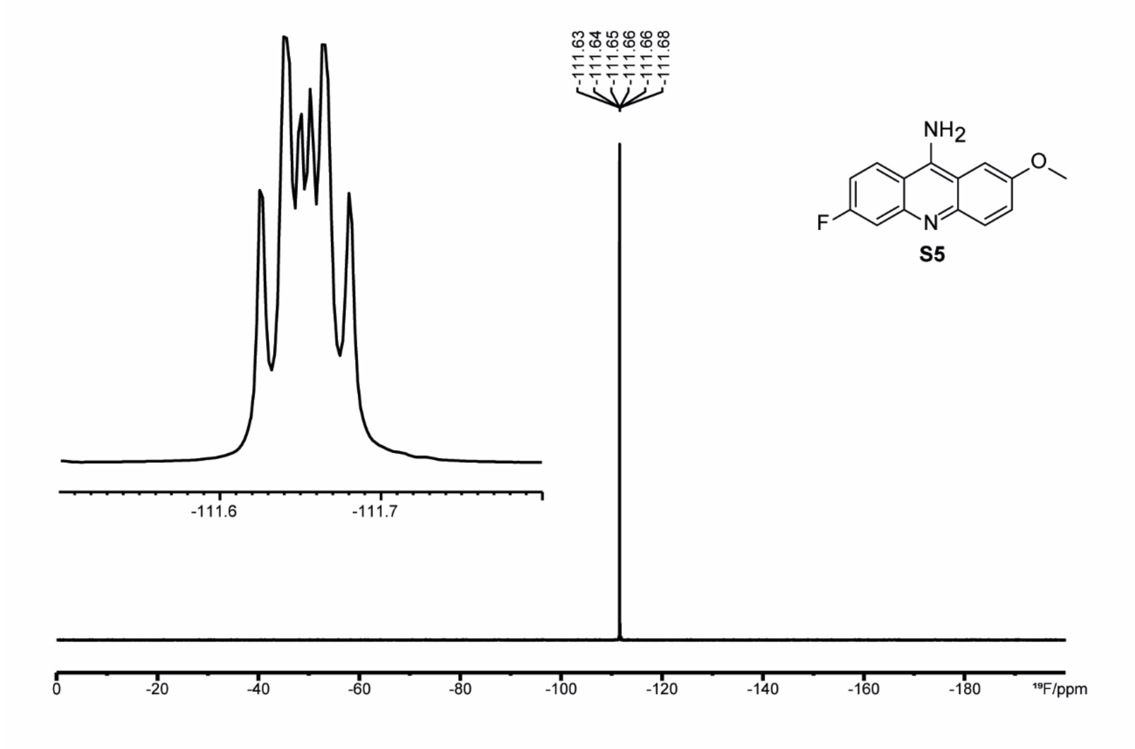

Supplementary Figure 49: ^{19}F-NMR spectrum of S5 (DMSO-d6, 298 K, 470.64 MHz).

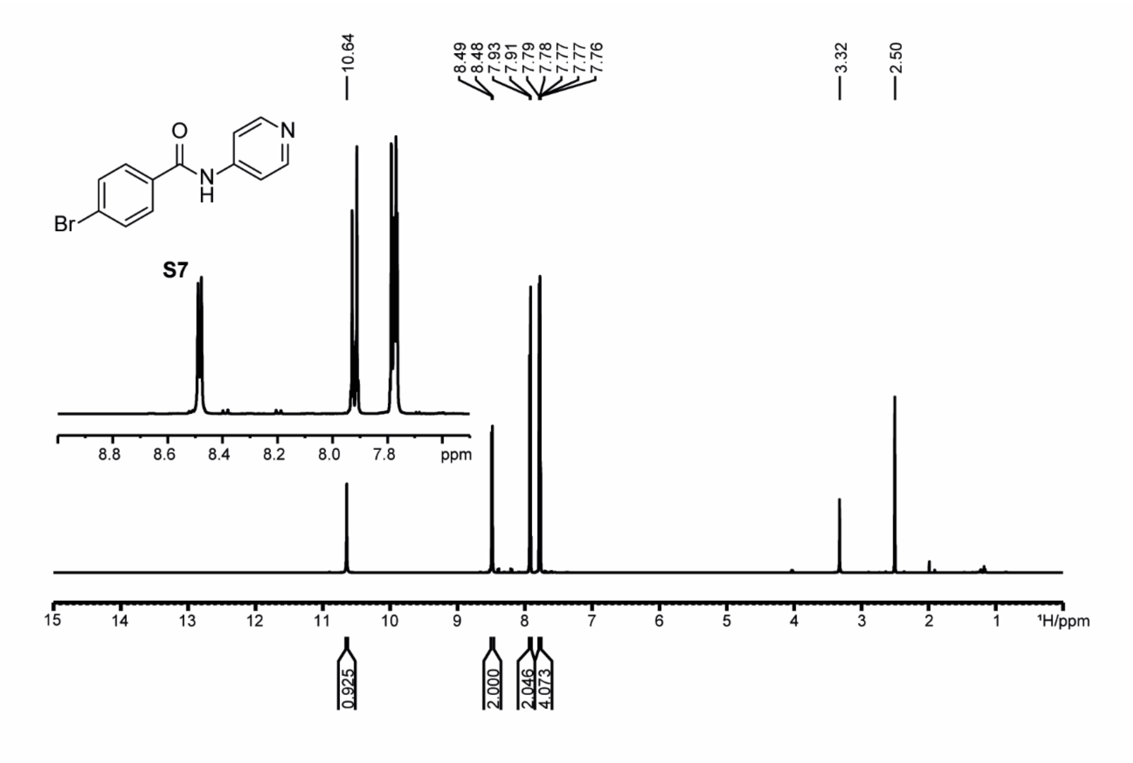

Supplementary Figure 50: ¹H-NMR spectrum of S7 (DMSO-d6, 298 K, 500.18 MHz).

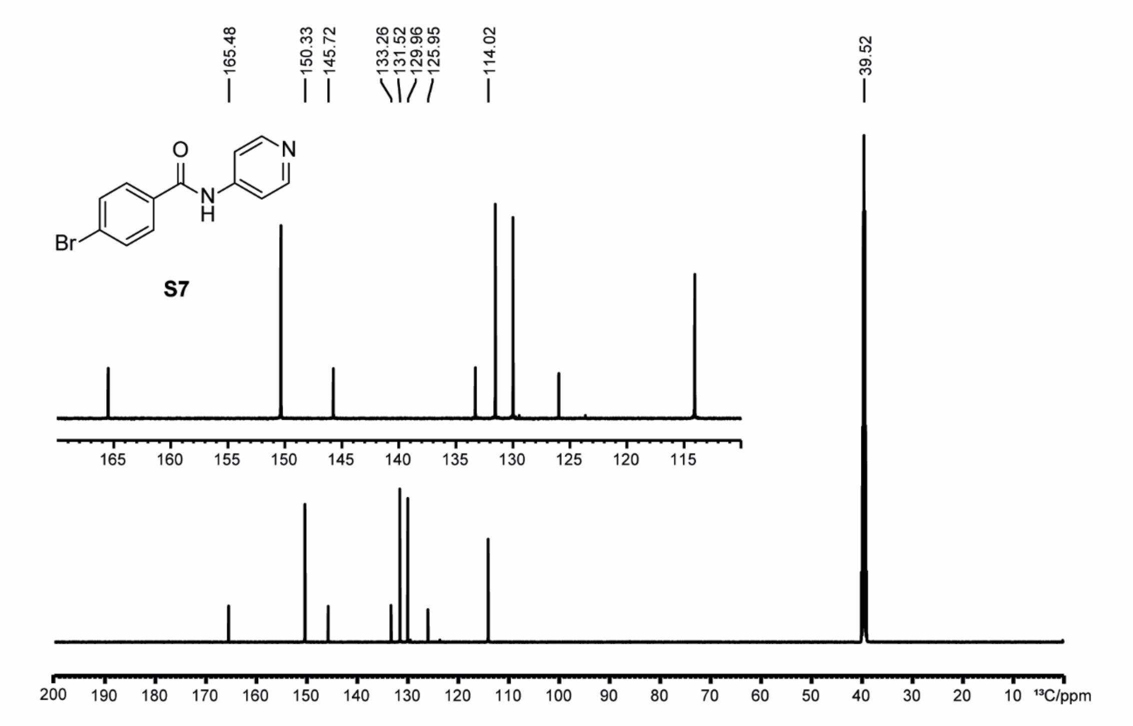

Supplementary Figure 51: ¹³C-NMR spectrum of S7 (DMSO-d6, 298 K, 157.77 MHz).

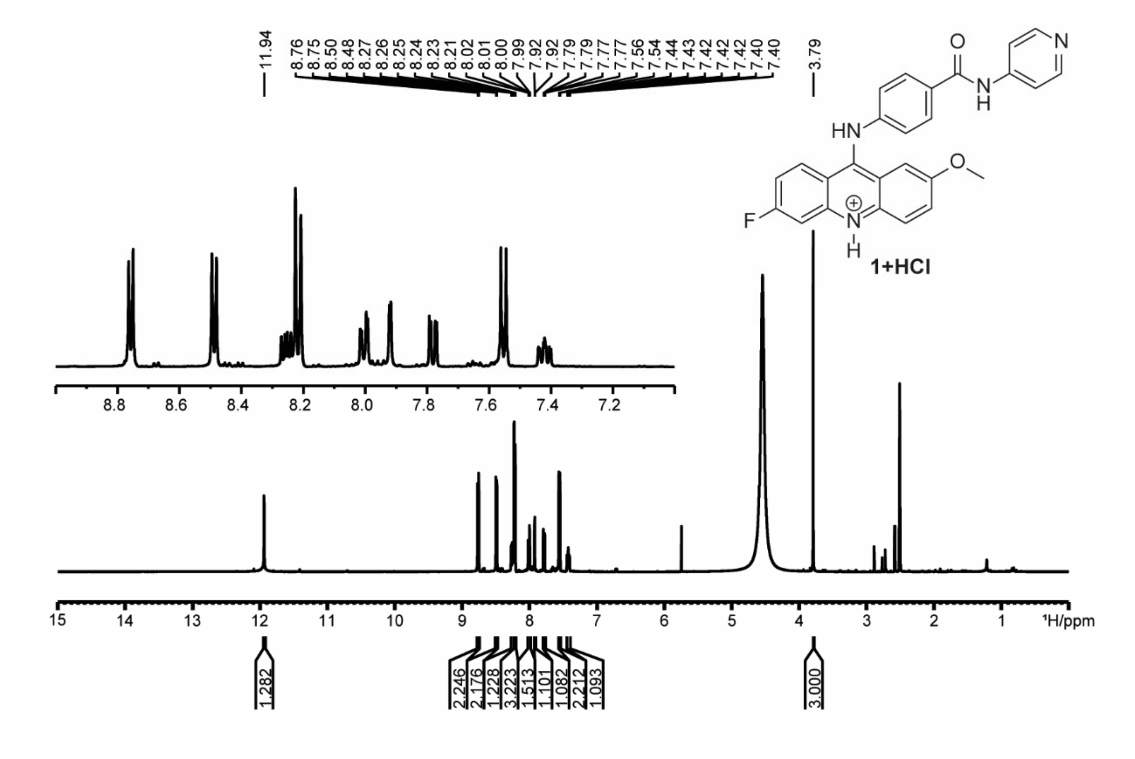

Supplementary Figure 52: ^{1}H-NMR spectrum of 1 with 2 vol.-% HCl added (DMSO-d6, 298 K, 500.18 MHz).

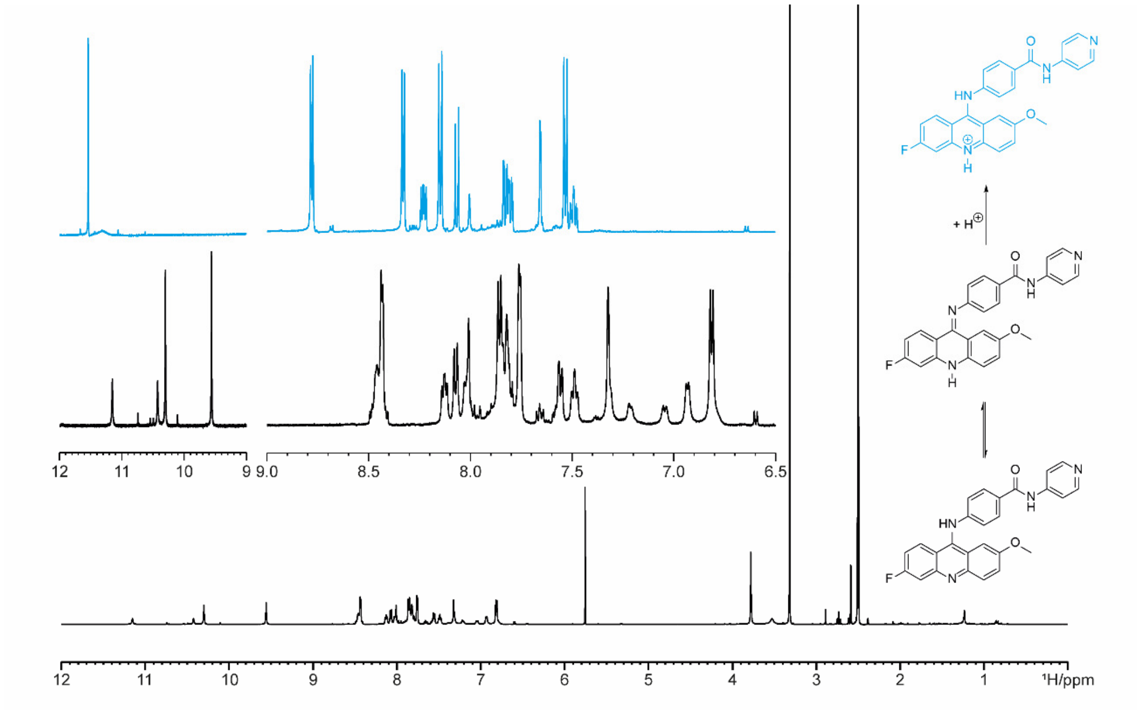

Supplementary Figure 53: NMR spectra of compound 1 in addition (blue) and without acid (black). The spectra were recorded in DMSO-d6 at 298 K (600.31 MHz). The isolated product contained two impurities after drying in oil-pump vacuum. One of those is DCM and the other could not be identified. ^{1}H and ^{13}C spectra show the presence of these impurities.

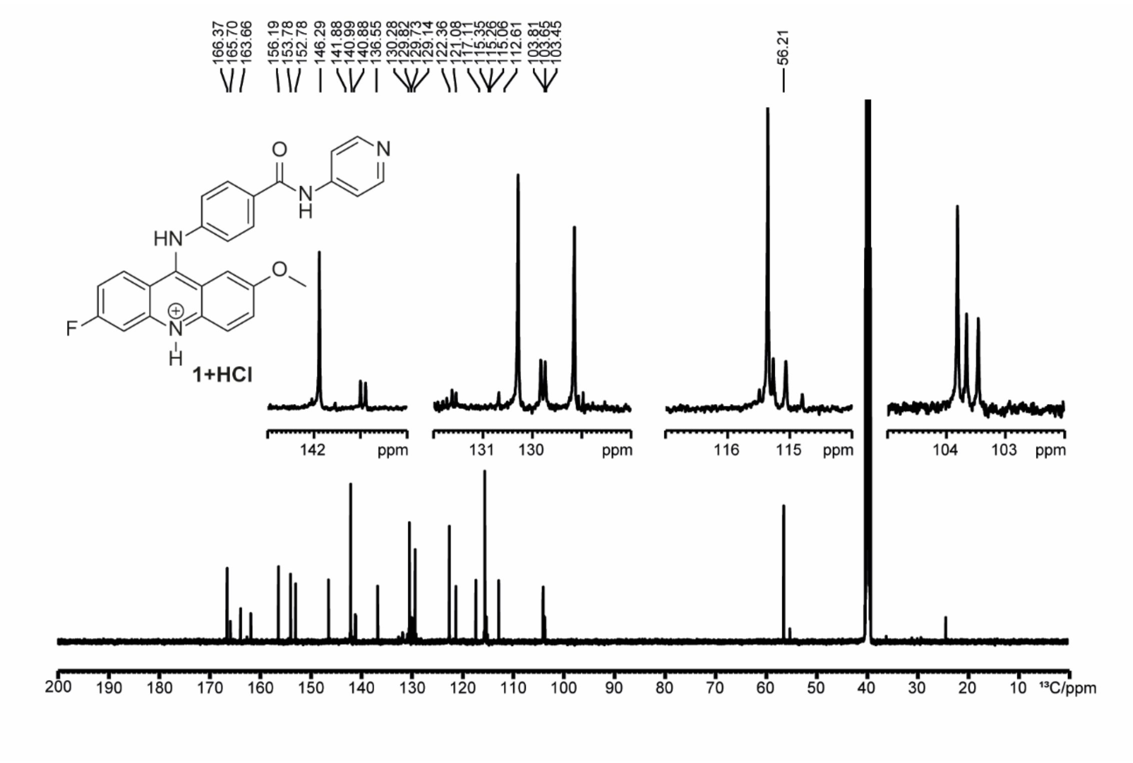

Supplementary Figure 54: ^{13}C-NMR spectrum of 1 with 2 vol.-% HCl added (DMSO-d6, 298 K, 157.77 MHz).

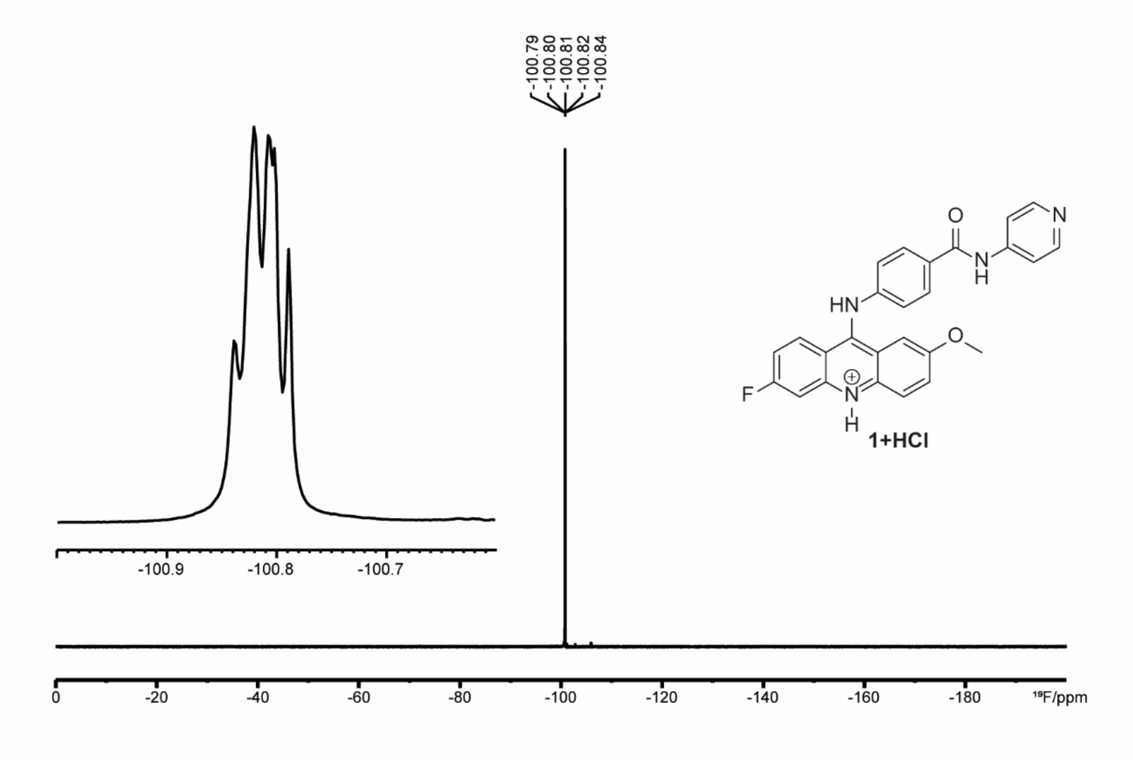

Supplementary Figure 55: ^{19}F-NMR spectrum of 1 with 2 vol.-% HCl added (DMSO-d6, 298 K, 470.64 MHz).

Fluorescence binding assay

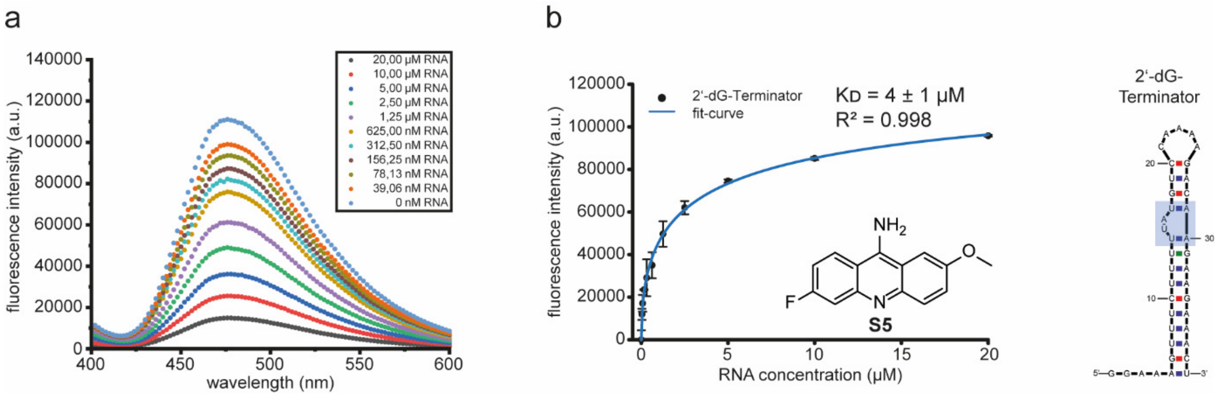

Supplementary Figure 56: *Determination of the dissociation constant. (a) Fluorescence titration and (b) binding curve of compound S5 to the 2'-dG-Terminator.*

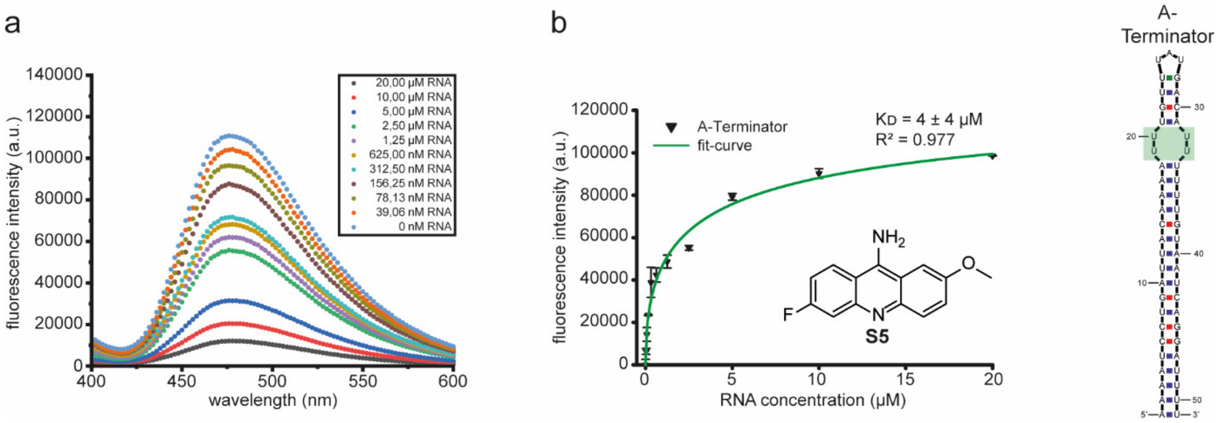

Supplementary Figure 57: Determination of the dissociation constant. (a) Fluorescence titration and (b) binding curve of compound S5 to the A-Terminator.

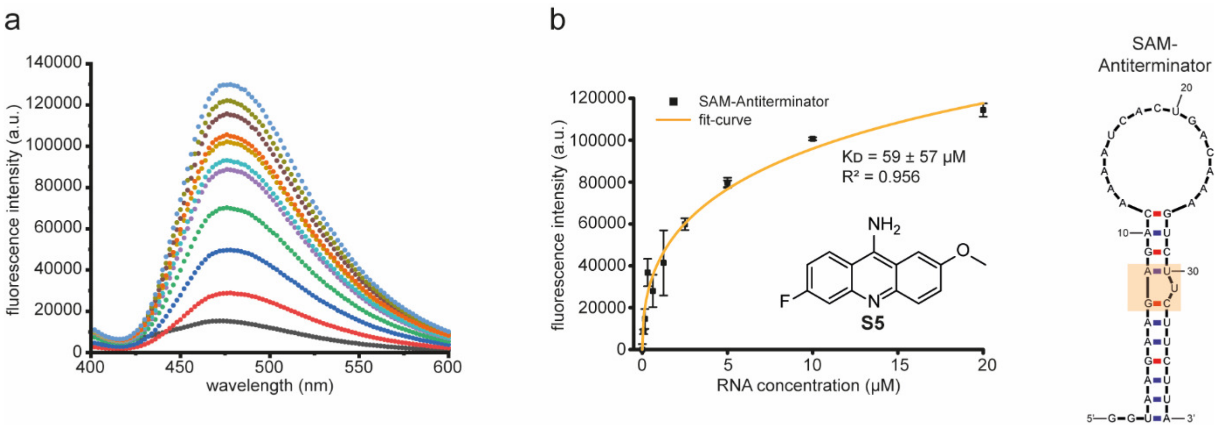

Supplementary Figure 58: Determination of the dissociation constant. (a) Fluorescence titration and (b) binding curve of compound S5 to the SAM-Terminator.

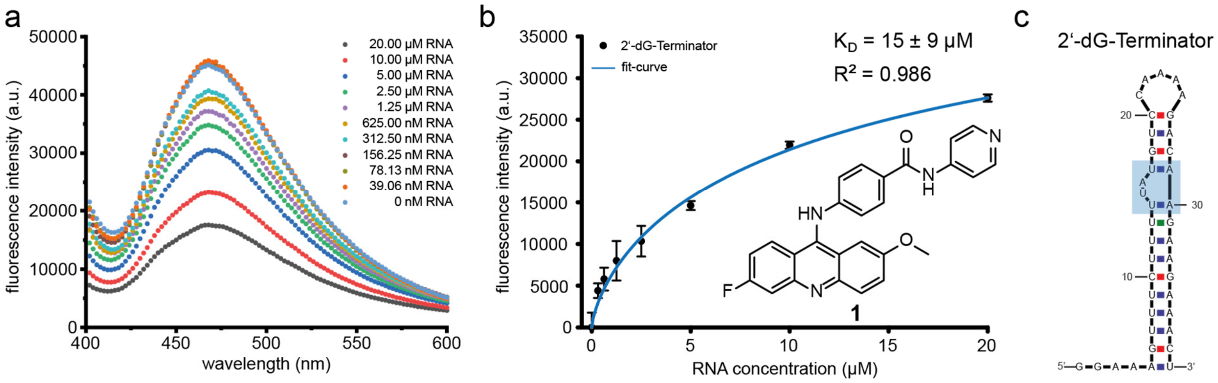

Supplementary Figure 59: Fluorescence-based determination of affinity to the 2'dG-Terminator stem of a fragment grown with an acridine moiety. (a) Individual UV-VIS spectra, (b) Determination of affinity of compound 1 to the 2'-dG-Terminator stem (c).

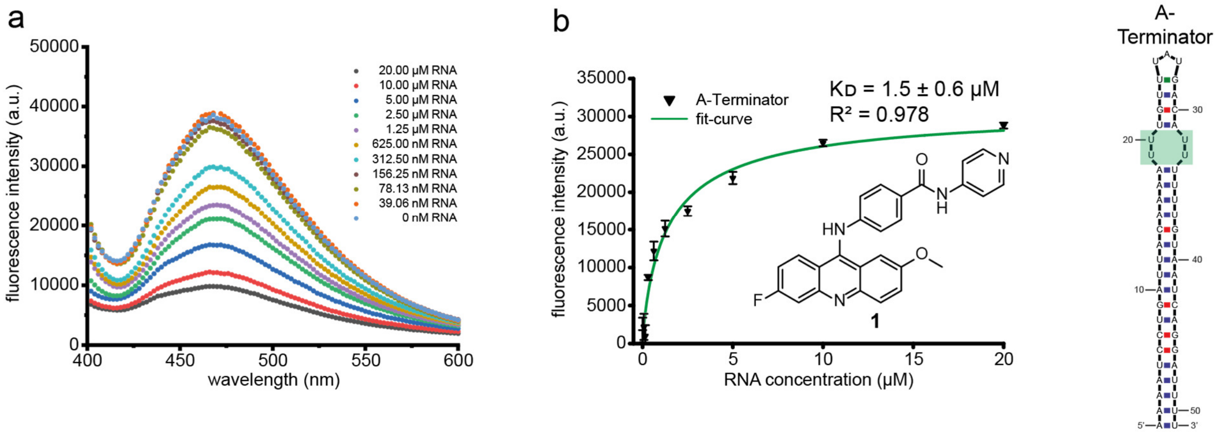

Supplementary Figure 60: Determination of the dissociation constant. (a) Fluorescence titration and (b) binding curve of compound 1 to the A-terminator.

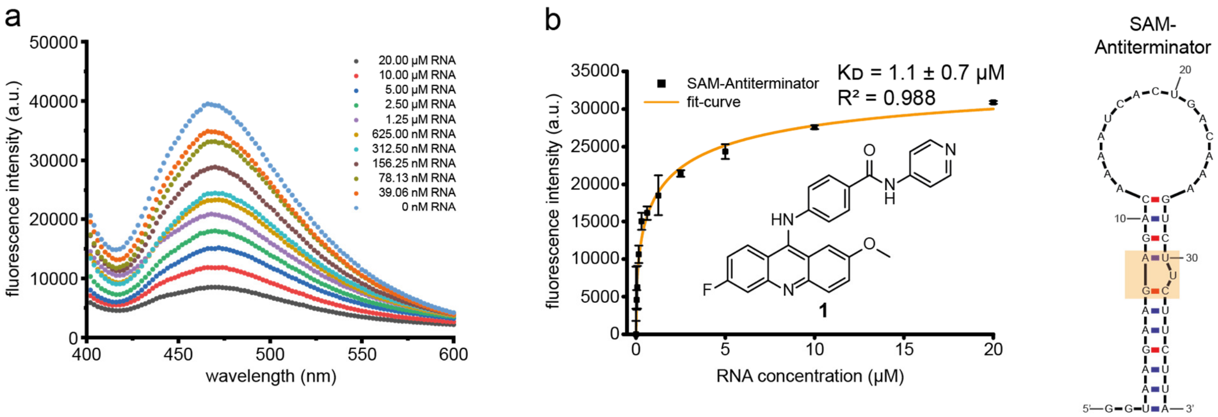

Supplementary Figure 61: Determination of the dissociation constant. (a) Fluorescence titration and (b) binding curve of compound 1 to the SAM-anti-terminator stem.

References

(1) Haider, M. R.; Ahmad, K.; Siddiqui, N.; Ali, Z.; Akhtar, M. J.; Fuloria, N.; Fuloria, S.; Ravichandran, M.; Yar, M. S. Novel 9-(2-(1-Arylethylidene)Hydrazinyl)Acridine Derivatives: Target Topoisomerase 1 and Growth Inhibition of HeLa Cancer Cells. *Bioorg. Chem.* **2019**, *88*, 102962. https://doi.org/10.1016/j.bioorg.2019.102962.

(2) Mohammadi-Khanaposhtani, M.; Rezaei, S.; Khalifeh, R.; Imanparast, S.; Faramarzi, M. A.; Bahadorikhalili, S.; Safavi, M.; Bandarian, F.; Nasli Esfahani, E.; Mahdavi, M.; Larijani, B. Design, Synthesis, Docking Study, α-Glucosidase Inhibition, and Cytotoxic Activities of Acridine Linked to Thioacetamides as Novel Agents in Treatment of Type 2 Diabetes. *Bioorg. Chem.* **2018**, *80* (March), 288–295. https://doi.org/10.1016/j.bioorg.2018.06.035.

(3) Bonse, S.; Santelli-Rouvier, C.; Barbe, J.; Krauth-Siegel, R. L. Inhibition of Trypanosoma Cruzi Trypanothione Reductase by Acridines: Kinetic Studies and Structure−Activity Relationships. *J. Med. Chem.* **1999**, *42* (26), 5448–5454. https://doi.org/10.1021/jm990386s.

(4) Gellerman, G.; Gaisin, V.; Brider, T. One-Pot Derivatization of Medicinally Important 9-Aminoacridines by Reductive Amination and SNAr Reaction. *Tetrahedron Lett.* **2010**, *51* (5), 836–839. https://doi.org/10.1016/j.tetlet.2009.12.020.

(5) Demidov, O. P.; Borovlev, I. V.; Amangasieva, G. A.; Avakyan, E. K. Oxidative SNH Amidation of Acridine and Tautomerism of N-(Acridin-9-Yl)Benzamides. *Chem. Heterocycl. Compd.* **2016**, *52* (2), 104–109. https://doi.org/10.1007/s10593-016-1841-7.
